# S100A14 promotes colorectal cancer progression and anti-PD-1 resistance via UPF1-mediated activation of the non-canonical NF-κB signaling

**DOI:** 10.1038/s41419-026-09032-1

**Published:** 2026-06-25

**Authors:** Li Tan, Xinglong Dai, Yujuan Wu, Zhong Zuo, Yong Cheng

**Affiliations:** 1 Department of Gastrointestinal Surgery, The First Affiliated Hospital of Chongqing Medical University, Chongqing, China; 2 Department of Cardiovascular Medicine, Cardiovascular Research Center, The First Affiliated Hospital of Chongqing Medical University, Chongqing, China

**Keywords:** Colorectal cancer, Oncogenes

## Abstract

Resistance to anti-PD-1 immunotherapy remains a critical challenge in colorectal cancer (CRC). However, the functional role of S100A14 in CRC remains controversial regarding its oncogenic status and impact on immunotherapy response. Here, integrating single-cell RNA sequencing and tissue microarray analysis, we definitively identify S100A14 as an oncogenic driver of immunotherapy resistance. High S100A14 expression correlates with poor prognosis and an immunosuppressive “cold” tumor phenotype. Mechanistically, S100A14 directly binds UPF1, a core nonsense-mediated mRNA decay (NMD) factor, promoting its ubiquitination-mediated degradation. This impairs the decay of non-canonical NF-κB transcripts (MAP3K14, NFKB2, RELB), causing constitutive pathway activation and PD-L1 upregulation. In co-culture assays, S100A14-overexpressing cells directly suppressed CD8^+^ T-cell cytotoxicity by downregulating GZMB and induced exhaustion via PD-1 upregulation. In syngeneic mouse models, S100A14 overexpression accelerated hepatic metastasis and conferred anti-PD-1 resistance by suppressing GZMB^+^ CD8^+^ T-cell infiltration and promoting Treg recruitment. Notably, restoring UPF1 expression reversed malignant phenotypes in vitro and rescued S100A14-mediated immune evasion in co-culture models. Collectively, we define the S100A14-UPF1-non-canonical NF-κB axis as a actionable mechanism driving immune evasion in CRC.

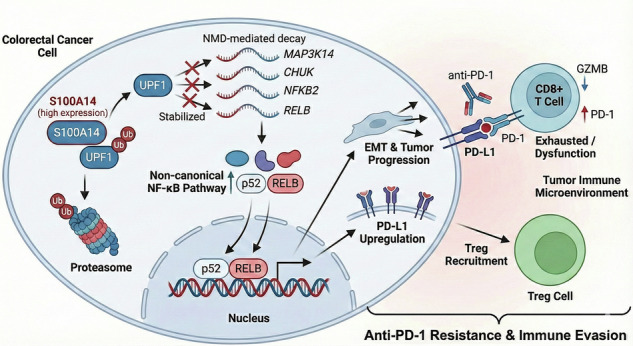

## Introduction

Colorectal cancer (CRC) is among the most life-threatening malignancies globally, ranking third in terms of occurrence rate and second in terms of mortality among various cancer types [1, 2]. Among all CRC patients, 45% develop synchronous or metachronous metastases, with a 5-year survival rate of less than 20% [3]. Immune checkpoint inhibitors (ICIs), particularly anti-PD-1 monoclonal antibodies (mAbs), serve as first-line treatments for metastatic CRC by disrupting immune evasion through the blockade of immunosuppressive signaling between tumor cells and immune cells [4, 5]. The KEYNOTE-177 study revealed that in MSI-H/dMMR (microsatellite instability-high/deficient mismatch repair) metastatic CRC, PD-1 blockade with pembrolizumab achieved an objective response rate of 45.1% and a complete response rate of 13.1%, significantly surpassing the chemotherapy group rates of 33.1% and 3.9%, respectively [6]. However, despite the use of anti-PD-1 mAbs within their approved indications, a considerable proportion of CRC patients develop primary or subsequent resistance. Therefore, investigating the mechanisms underlying anti-PD-1 resistance in CRC is crucial for enhancing immunotherapy efficacy and expanding the application of cancer immunotherapies.

The S100 protein family plays critical intracellular roles in regulating cell proliferation, apoptosis, invasion, and motility [7, 8]. As a member of the S100 protein family, S100A14 is negatively correlated with prognosis in patients with lung cancer, breast cancer, ovarian cancer, and hepatocellular carcinoma [9–12]. However, its role in CRC remains controversial. Wang et al. reported that loss of S100A14 expression is correlated with poor prognosis [13], whereas Diamantopoulou’s study demonstrated that elevated S100A14 expression is associated with shorter disease-specific survival (DSS) and recurrence-free survival (RFS) [14]. In this study, through systematic analysis of scRNA-seq data, we successfully identified an immune-resistant tumor epithelial cell subpopulation and determined that S100A14 serves as a critical molecular mediator of immunotherapy resistance in CRC. Thus, the functional importance of S100A14 in CRC warrants further investigation.

In this study, multiplex immunohistochemical (mIHC) analysis of a tissue microarray (TMA) further confirmed that S100A14 was significantly overexpressed in CRC tissues compared with adjacent normal tissues. Moreover, CRC patients with high S100A14 expression had poor overall survival (OS), and S100A14 levels were significantly negatively correlated with tumor-infiltrating CD3^+^ and CD3^+^ CD8^+^ cells. Through both in vivo and in vitro experiments, it was demonstrated that S100A14 could enhance the proliferative, migratory, and invasive capacities of CRC cells while conferring resistance to anti-PD-1 therapy. Mechanistically, S100A14 directly binds to UPF1 and promotes its ubiquitination-mediated degradation, thereby impairing the UPF1-dependent mRNA decay of noncanonical NF-κB pathway components. This process led to the upregulation of NF-κB signaling and subsequent PD-L1 overexpression. These findings elucidate the role of S100A14 in CRC progression and reveal its critical function in antitumor immune suppression, providing novel insights for overcoming immunotherapy resistance in CRC patients.

## Methods

### Ethics statement

The TMA chip samples in this study were provided by AiFang Biological (Ethics Number: HN20250401). Written informed consent was obtained from all human participants prior to tissue collection. All research conformed to the Helsinki Declaration(https://www.wma.net/policies-post/wma-declaration-of-helsinki-ethical-principles-for-medical-research-involving-human-subjects/). All animal experiments were conducted in accordance with institutional guidelines and approved by the Institutional Animal Care and Use Committee (IACUC) of Chongqing Medical University (Approval No. IACUC-CQMU-2024-0729).

### Quality control and integration of ScRNA-seq sequencing data

scRNA-seq data (GSE205506) obtained from the Gene Expression Omnibus (GEO) database were preprocessed using the Seurat v4 R package. The dataset comprised 27 CRC samples, including 5 non-pCR samples (1 pre-treatment and 4 post-treatment) and 22 pCR samples (9 pre-treatment and 13 post-treatment). Multiple quality control measures were implemented to filter low-quality data: (1) cells expressing fewer than 500 or more than 5000 genes were excluded; (2) cells with mitochondrial gene expression ≥20% or erythrocyte gene expression ≥5% were removed; and (3) only cells containing 400–25,000 unique molecular identifiers (UMIs) were retained for subsequent analysis. To integrate single-cell data from different samples into a shared low-dimensional space, batch effect correction and normalization were performed using Seurat’s reciprocal principal component analysis function “IntegrateData”. The final integrated matrix was used for clustering analysis and cell type classification.

### Unsupervised clustering analysis and broad cell type identification

Following the generation of the integrated matrix, unsupervised graph-based clustering analysis was performed using default parameters in Seurat unless otherwise specified. The workflow was as follows: (1) UMI count matrices were normalized using the “NormalizeData” function (default settings); (2) variable genes were selected via the “FindVariableFeatures” function using the vst method; (3) 3000 variable genes were selected for all cell type clustering analyzes; (4) after regressing out UMI counts, dimensionality reduction was applied to the dataset; (5) clustering was performed using the “FindClusters” function with 20 principal components and a resolution of 0.5; and (6) marker genes for each cell subpopulation were identified using the “FindAllMarkers” function—the top 50 marker genes were used for cell type annotation (annotated via the ACT database: http://xteam.xbio.top/ACT/). Detailed cell type markers are provided in Table S1. The proportions of cell types between the two groups were visualized using the ggplot2 package.

### TMA and patient characteristics

A CRC TMA was constructed, comprising 65 paired samples of primary tumors and matched liver metastases collected between January 2018 and September 2018. Follow-up data were consolidated in September 2022. The primary endpoint was OS, calculated from the date of CRC resection to the date of death or the last follow-up. The TMA was primarily utilized for multiplex IHC analysis.

### mIHC

The collected tissue sections were dewaxed with conventional xylene and hydrated with gradient alcohol. MIHC of TMA was performed using a five-color multiplex immunofluorescence staining kit (AFIHC025, AiFang Biological, China). According to the manufacturer’s instructions, the antigens were exposed to microwave repair. The sections were incubated with a 3% hydrogen peroxide solution at room temperature for 15 min, and then dropped with 10% goat serum for blocking for 15 min. The primary antibody A was added and incubated overnight at 4 °C, and then washed three times with PBST. Polymer-HRP anti-mouse/rabbit universal secondary antibody IgG (AFIHC001, AiFang Biological) was dropped, and the sections were incubated at room temperature for 30 min. Subsequently, the sections were washed with PBST, and the TYR fluorescent dye was dropped and reacted for 8 min, followed by three washes with PBST. Antibody A was removed by microwave treatment, and the sections were washed three times with PBST. After dropping goat serum for blocking, the primary antibody B was added, and the operation was repeated until all the antigens, including CK-Pan (AF20164, 1:4000, AiFang Biological), S100A14 (10489-1-AP, 1:2000, Proteintech), CD3 (ab16669, 1:500, Abcam), CD8 (AF20211, 1:2000, AiFang Biological), were completely visualized. DAPI staining solution was dropped, and the sections were incubated at room temperature in the dark for 10 min, followed by three washes with PBST. The sections were mounted with an anti-fluorescence quenching mounting medium, and the mIHC slides were imaged using an eight-channel fluorescence digital slide scanner (model AF-KL-20-8, AiFang Biological, China).

### mIHC image acquisition and quantification

During the image acquisition stage, an eight-channel fluorescence digital slide scanner (AF-KL-20-8, AiFang Biological, China) was used to collect images of mIHC slides, and the acquired images were saved in the .kfbf format.

In the image data analysis stage, custom algorithms were developed in the VISIOPHARM software (VISIOPHARM, Hoersholm, Denmark) to conduct data analysis on the images. To ensure the consistency of the analysis results, fixed and uniform threshold parameters were set through the VISIOPHARM application to identify and quantitatively analyze S100A14^+^, CD3^+^, CD8^+^ and CD3^+^ CD8^+^ cells. After the analysis was completed, the analysis results were exported. After data collection, GraphPad Prism was used for the statistical analysis of the data.

### Cell culture and viral infection

The human CRC cell line HCT116 (RRID: CVCL_0291) was purchased from the Cell Bank of the Chinese Academy of Sciences (Shanghai, China) in March 2024. The murine CRC cell lines CT26 RRID: CVCL_7256) and MC38 (RRID: CVCL_B288) were purchased from iCell Bioscience Inc (Shanghai, China) in April 2024. All cell lines were confirmed to be free from contamination. HCT116 and MC38 cells were cultured in DMEM (Dulbecco’s Modified Eagle Medium) supplemented with 10% fetal bovine serum (FBS) (Excell, China) and 1% penicillin/streptomycin (Gibco, USA), while CT26 cells were maintained in RPMI-1640 medium. Cells were maintained in a humidified incubator at 37 °C with 5% CO₂.

All cell lines used in this study were confirmed to be free of mycoplasma contamination. The lentiviral packaging plasmids, including the pLKO.1-CMV-copGFP-PURO-based shRNA (short hairpin RNA) targeting human and murine S100A14, as well as the PCDH-CMV-EF1-Puro-based lentiviral shuttle plasmids for S100A14 overexpression and dual overexpression of S100A14 and UPF1, were purchased from Tsingke Biotechnology Co., Ltd (Beijing, China). For lentiviral infection, 2 × 10⁵ cells were plated and infected for 48 h, followed by puromycin selection (2 µg/mL for HCT116, 4 µg/mL for CT26, and 10 µg/mL for MC38) for 2 days to establish stable cell lines. The shRNA/siRNA sequences are provided in Table S2.

### CCK-8 and colony formation assays

For the CCK-8 assay, cells were seeded at a density of 1000 cells per well in 96-well plates. Cell proliferation was measured at 0, 24, 48, 72, and 96 h after cell attachment using 10 µL of the Cell Counting Kit-8 (CCK-8) solution (APExBIO, USA). After incubation at 37 °C with 5% CO₂ for 2 h, absorbance was measured at 450 nm. Results were normalized to the absorbance at 0 h.

For the colony formation assay, 1000 cells were seeded in 6-well plates and cultured at 37 °C with 5% CO₂. When visible colonies formed (approximately 7 days), cells were fixed with 4% paraformaldehyde and stained with 0.1% crystal violet.

### EdU assay

EdU Cell Proliferation Kit (Beyotime, China) was used to assess cell proliferation. Briefly, cells were seeded in 96-well plates and incubated overnight at 37 °C with 5% CO₂. Subsequently, 50 µM EdU solution was added to each well, and the plates were incubated for 2 h under the same conditions. After washing three times with PBS containing 3% bovine serum albumin, cells were fixed with 4% paraformaldehyde for 30 min at room temperature. Permeabilization was performed using 0.3% Triton X-100. Cells were then stained with Azide 594 and Hoechst. Fluorescence images were acquired using a scanning fluorescence microscope. Data analysis was performed using ImageJ (v1.54) and GraphPad Prism (v10.1.2).

### Transwell assay

For the Transwell migration assay, 5 × 10^4^ cells were suspended in 200 µL of serum-free DMEM and seeded into the upper chamber (Biofil, China), while the lower chamber was filled with 600 µL of DMEM containing 10% FBS. After 24–72 h, the migrated cells were fixed with 4% paraformaldehyde, stained with 1% crystal violet, and imaged. For the Transwell invasion assay, the upper chamber was pre-coated with Matrigel (Beyotime, China), and the cell treatment procedure was performed as described above. The results were analyzed using ImageJ (v1.54) and GraphPad Prism (v10.1.2).

### Wound healing assay

Cells were seeded into 6-well plates and incubated until reaching at least 90% confluency. A sterile pipette tip was used to create a scratch wound on the monolayer. The detached cells were washed away with PBS, and serum-free DMEM was added for further incubation. Images were captured at 0 and 24 h post scratching. The migration distance was quantified using ImageJ (v1.54) and GraphPad Prism (v10.1.2).

### WB analysis

Cultured cells were lysed using RIPA buffer supplemented with phosphatase inhibitors (Beyotime, China) and protease inhibitors (Sparkjade, China). After centrifugation (12,000 rpm, 15 min), the protein concentration in the supernatant was determined using the bicinchoninic acid assay (Sparkjade, China). Proteins (20–40 µg) were separated by SDS-PAGE and transferred onto a nitrocellulose membrane. The membrane was blocked with 5% skim milk in TBST or a protein-free rapid blocking buffer (Yeasen, China). After overnight incubation with primary antibodies at 4 °C, the membrane was probed with HRP-conjugated secondary antibodies (1:10,000, Immunoway) and visualized using an ECL chemiluminescent substrate (Oriscience, China). Protein band intensities were quantified by densitometry using ImageJ (v1.54) and normalized to GAPDH. Detailed antibody information is provided in Table S3.

### IP-MS

Flag-S100A14 was immunoprecipitated from HCT116 cells overexpressing S100A14 using an anti-Flag antibody (Immunoway, USA). Target protein bands were excised from sodium dodecyl sulfate‑polyacrylamide gel electrophoresis (SDS-PAGE) gels following electrophoresis. Purified peptides were resuspended in 0.1% formic acid aqueous solution for LC-MS/MS analysis.

LC-MS/MS was performed using an Orbitrap Exploris 480 mass spectrometer (Thermo Fisher Scientific, MA, USA) coupled with an EASY-nanoLC 1200 system. The mass spectrometer operated in data-dependent acquisition mode, automatically switching between MS and MS/MS scans. Instrument parameters were configured as follows: (1) MS: Scan range (m/z): 350–1200; Resolution: 120,000; Normalized AGC target: 300%; Maximum injection time: 100 ms (2) HCD-MS/MS: Resolution: 30,000; Normalized AGC target: 200%; Maximum injection time: 50 ms; Collision energy: 25%, 30%, 35%; Dynamic exclusion: 30 s. Mass spectra were analyzed using PEAKS Studio version 10.6 (Bioinformatics Solutions Inc., Waterloo, Canada). All candidate proteins are listed in Table S4.

### Co-IP

Cell lysates were incubated with target antibodies overnight at 4 °C. Antigen-antibody complexes were then bound to Protein A/G magnetic beads (HY-K0202, MCE) for 1 h at room temperature. Bead-lysate mixtures were washed with 0.5% PBST, followed by antigen-antibody complex elution at 95 °C for 10 min. Co-IP products were analyzed by WB.

### qPCR

Total RNA was extracted from cells and tissues using TRIzol reagent (Invitrogen, USA) according to the manufacturer’s protocol. cDNA was synthesized using the PrimeScript 1st Strand cDNA Synthesis Kit (Takara, Japan) and served as a template for real-time PCR with TB Green Premix (Takara, Japan). ACTB was used as the endogenous control gene. Relative gene expression was calculated using the 2^−ΔCt^ or 2^−ΔΔCt^ method. Primer sequences are provided in Table S5.

### RIP

RIP assays were performed using the PureBinding RNA Immunoprecipitation Kit (P0101, Geneseed) to analyze the binding of UPF1 to specific RNAs in HCT116 and MC38. Briefly, cell lysates prepared in RIP lysis buffer containing protease/RNase inhibitors were incubated overnight at 4 °C with 5 µg anti-UPF1 antibody (A5071, Abclonal) or equivalent control IgG (HA1002, Huabio). Immune complexes were captured with Protein A/G magnetic beads, washed extensively, and the co-precipitated RNAs were purified. Enrichment of target RNAs was quantified by qRT-PCR using gene-specific primers (Table S5), with data normalized to input samples and presented as fold enrichment (log2fold-change) relative to IgG control. Experiments included four biological replicates.

### Animal experiments

Male C57BL/6J mice aged 6–8 weeks were obtained from the Experimental Animal Center of Chongqing Medical University. For the xenograft tumor model, mice were randomly assigned to groups (*N* = 5). A total of 1 × 10^6^ MC38 cells were subcutaneously injected into the right axillary region. The major and minor axes of the subcutaneous tumors were measured on days 6, 8, 11, and 14 post injection, and tumor volume was calculated using the formula *V* = (width^2^×length)/2. Mice in the anti-PD-1 treatment group received intraperitoneal injections (100 µg/mouse, diluted in PBS; HY-P99144, MCE) on days 8, 11, and 14 post tumor inoculation. On day 17, mice were euthanized, and subcutaneous tumors were excised, weighed, and measured for mass and volume. Tumors were fixed in 4% formaldehyde solution.

A liver metastasis model was employed to assess the metastatic potential of cancer cells. Mice were randomly allocated (*N* = 3) and injected with 2 × 10^5^ MC38 cells into the spleen of C57BL/6J mice. After 25 days, mice were sacrificed. Liver tissue sections were stained with hematoxylin and eosin (H&E) and examined under a microscope for tumor colonies.

No formal statistical power calculation was conducted prior to the experiment. The group sizes were chosen based on practical considerations of animal availability, logistical resources for genotyping and molecular analyzes. There were no exclusions of animals.

### Tissue dissociation and flow cytometric analysis

Freshly excised mouse tumors (100 mg) were immediately dissociated using a tissue dissociation solution (AC05L322, Life-iLab). The digested cells were filtered through a 70 μm strainer, washed with DMEM, and centrifuged twice at 1200 rpm for 5 min. The supernatant was discarded, and cells were resuspended in cell staining buffer.

For intracellular staining, cells were fixed with Fixation Working Solution (E-CK-A108A, Elabscience) and permeabilized with Permeabilization Working Solution (E-CK-A108C, Elabscience). After incubation with antibodies at room temperature for 30 min in the dark, cells were washed twice with cell staining buffer and resuspended in 200 µL of the same buffer. Samples were analyzed using a flow cytometer (CytoFLEX LX, Beckman Coulter, Indianapolis, IN, USA) and CytExpert software (Beckman Coulter). The percentage of each cell population was quantified using GraphPad Prism 10.1.2 software. Detailed antibody information is provided in Table S3.

### IF

The collected tissue sections were deparaffinized with xylene and rehydrated through a graded alcohol series. Antigen retrieval was performed via microwave irradiation, followed by incubation with 3% hydrogen peroxide at room temperature for 15 min. For cell samples, cells (5 × 10^4^) were seeded onto confocal dishes (Biosharp, China) and fixed with 4% paraformaldehyde for 15–20 min. Subsequently, the slides or coverslips were blocked with PBS containing 5% goat serum at room temperature for 1 h. For double IF staining, the slides or coverslips were incubated overnight at 4 °C with a mixture of two primary antibodies. The primary antibodies used included: Foxp3 (AFRP0014, AiFang Biological), CD4 (AF20210, AiFang Biological), GZMB (AFRM0352, AiFang Biological), and CD8 (AFRM0004, AiFang Biological). After washing with cold PBS, the slides or coverslips were incubated in the dark at room temperature for 1 h with a mixture of two secondary antibodies derived from different species. Nuclei were counterstained with 4′,6-diamidino-2-phenylindole (DAPI) (Beyotime, China).

### Statistical analysis

No statistical methods were used to predetermine sample sizes. Rather, sample sizes for in vitro and in vivo experiments were determined based on empirical estimation from prior similar studies in the field and our institutional experience to ensure adequate reproducibility. For clinical samples (e.g., TMA), the sample size was dictated by the availability of appropriately matched tissue specimens. All animals and samples assigned to experimental groups were included in the final analyzes, and no data points, samples, or animals were excluded. Investigators were not blinded to group allocation during the experiments and outcome assessments, as knowledge of the genetic modifications and treatment groups was required for the proper administration of targeted interventions and daily animal care. All experiments were independently repeated in triplicate. Statistical analyzes were performed using GraphPad Prism (version 10.1.2) or SPSS (version 26.0). For comparisons between two groups, two-tailed Student’s *t*-tests were used to calculate *P*-values. For comparisons among multiple groups, one-way analysis of variance was employed. Correlation analyzes were conducted using Spearman’s rank correlation coefficient. Survival curves were generated using the Kaplan-Meier method and compared via the log-rank test. Multivariate survival analysis was performed using Cox proportional hazards regression models to evaluate independent prognostic factors. Quantitative data are presented as the mean ± standard deviation (SD) from at least three independent experiments unless otherwise specified. Estimates of variation within each group are visualized as error bars in all applicable corresponding figures. In all cases, a *P*-value < 0.05 was considered statistically significant.

## Results

### S100A14 correlates with anti-PD-1 resistance in CRC

**Fig. 1:**
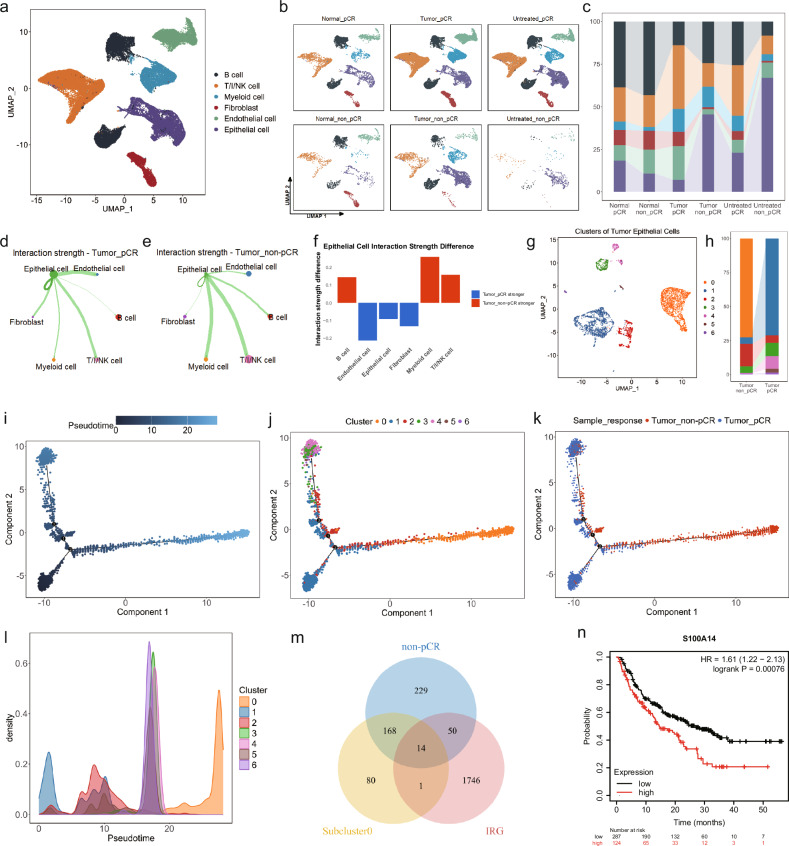
S100A14 is correlated with anti-PD-1 resistance in CRC. **a–c** UMAP plots illustrating the cellular clusters in CRC tumor before and after anti-PD-1 treatment and adjacent normal tissues after anti-PD-1 treatment (**a**, **b**) and a bar chart showing the corresponding cell proportions (**c**). **d–f** Cell chat analysis between epithelial cells and other cell types in post-treatment tumors from the pCR (**d**) and non-pCR (**e**) groups, along with the corresponding statistical analysis (**f**). **g, h** UMAP plot depicting the subclustering of epithelial cells in post-treatment tumors (**g**) and a bar chart of the subcluster proportions (**h**). **i–l** Pseudotime analysis trajectory plots colored by pseudotime (**i**), cell subcluster (**j**), and treatment response (**k**), accompanied by a density distribution plot showing the prevalence of different cell subtypes along the pseudotime trajectory (**l**). **m** Venn diagram illustrating the intersection of differentially expressed genes. “non-pCR”: upregulated differentially expressed genes in non-pCR CRC epithelial cells post-treatment relative to these in pCR group; “subcluster 0”: genes highly expressed in cell subcluster 0 relative to other subclusters; “IRG”: immune-related gene set. **n** Analysis of the KM database indicating that patients with high S100A14 expression exhibit poorer prognosis when treated with anti-PD-1 therapy.

To identify key molecules contributing to anti-PD-1 resistance in CRC, we reanalyzed a landmark scRNA-seq dataset derived from the NCT03926338 clinical trial cohort [15]. This comprehensive cohort encompasses patients with locally advanced CRC who underwent neoadjuvant PD-1 blockade therapy at the Sixth Affiliated Hospital of Sun Yat-sen University, providing a robust transcriptomic landscape for evaluating immunotherapy responses. Cells were manually annotated based on the top 50 marker genes of each cell subpopulation and classified into six major types. It was observed that tumors from patients in the pathological complete response (pCR) group exhibited relatively higher proportions of T/I/NK cells both before and after anti-PD-1 treatment, whereas the non-pCR group showed a higher proportion of epithelial cells (Fig. 1a–c). Cell chat analysis of epithelial cells in post-treatment tumors revealed that communication intensity between epithelial cells and immune cells (T/I/NK, B, and myeloid cells) was enhanced in the non-pCR group (Fig. 1d–f). This suggests that epithelial cells may play an important role in resistance to PD-1 blockade. Further subclustering of epithelial cells in post-treatment tumors identified seven distinct subclusters (Fig. 1g). Subcluster 0 was most abundant in the non-pCR group but nearly absent in the pCR group (Fig. 1h). Pseudotime trajectory analysis indicated that subpopulation 0 originated from the non-pCR group and emerged at the terminal stage of anti-PD-1 treatment, suggesting its potential involvement in treatment resistance (Fig. 1i–l).

By intersecting highly expressed genes in post-treatment non-pCR tumor epithelial cells, marker genes of subpopulation 0, and immune-related genes, 14 candidate genes were identified (Fig. 1m). Further survival analysis of anti-PD-1-treated tumor patients from the KM database revealed that high expression of S100A14 was associated with poorer prognosis, indicating its potential key role in anti-PD-1 therapy resistance in CLC (Fig. 1n).

### S100A14 is upregulated in CRC, with high expression indicating reduced immune cell infiltration and poor prognosis

**Fig. 2:**
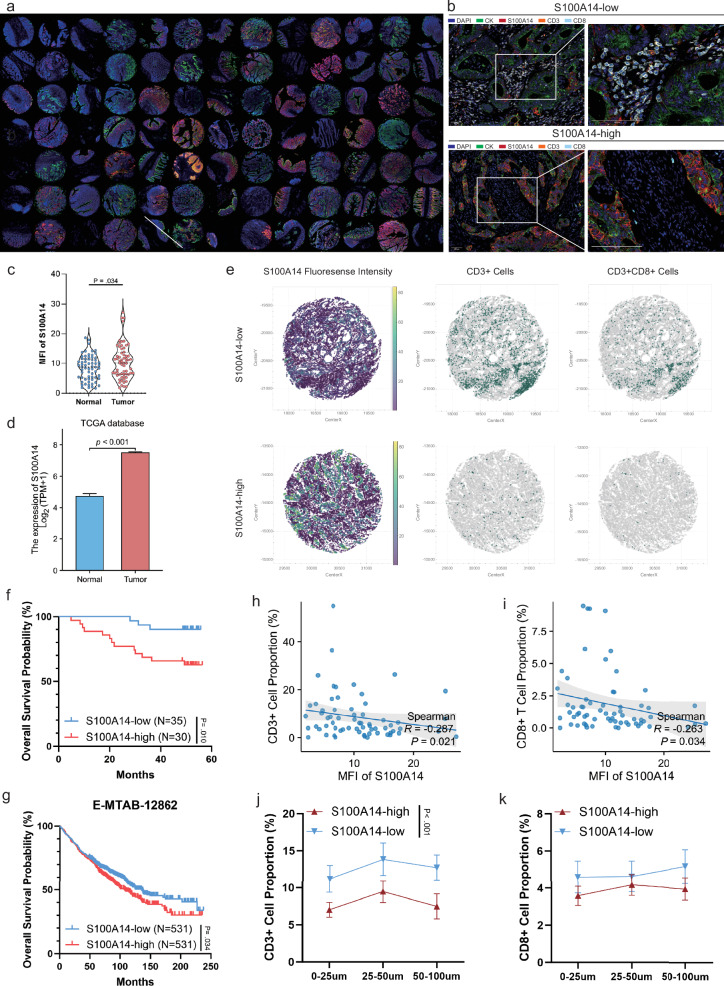
S100A14 is upregulated in CRC, with high expression indicating reduced immune cell infiltration and poor prognosis. **a**, **b** Representative images of multiplex IF staining of the CRC TMA and the identification strategy for tumor-infiltrating CD3^+^ and CD8^+^ cells are shown. Each marker is represented by a different color, as shown in the panel. **c** Differential expression of S100A14 in CRC tissues versus paired normal tissues (*N* = 65). **d** Differential expression of S100A14 in CRC versus normal tissues from the TCGA database. **e** Representative images showing the fluorescence intensity of S100A14 (left), the proportion of CD3^+^ tumor-infiltrating cells (middle), and the proportion of CD3^+^ CD8^+^ cells (right) in two CRC cases. **f, g** Kaplan‒Meier analysis of CRC patients stratified by low vs. high S100A14 expression levels in the TMA (**f**) and E-MTAB-12862 datasets (**g**) (log-rank test). **h, i** Correlation analysis between S100A14 expression and the proportions of CD3^+^ (**h**) and CD3^+^ CD8^+^ (**i**) tumor-infiltrating cells (*N* = 65). **j, k** Differences in the proportions of CD3^+^ (**j**) and CD3^+^ CD8^+^ (**k**) tumor-infiltrating cells at varying peritumoral distances between the S100A14 high- and low-expression groups (*N* = 65).

Having identified S100A14 as a potential candidate associated with anti-PD-1 resistance via single-cell transcriptomic analysis, we next performed mIHC on a TMA containing 65 paired CRC and adjacent normal tissues to evaluate its prognostic importance and role in the immune microenvironment (Fig. 2a, b). Quantitative analysis of the mean fluorescence intensity revealed significantly higher S100A14 expression in CRC tissues than in adjacent normal tissues (*P* = 0.034) (Fig. 2c), which is consistent with data from The Cancer Genome Atlas (TCGA) database (Fig. 2d).

Using the optimal cutoff value of 9.78 (determined by receiver operating characteristic curve analysis for survival prediction), patients were stratified into S100A14-high (*N* = 30) and S100A14-low (*N* = 35) groups (Fig. S1). The S100A14-high group had a higher mortality rate (37.14% vs. 10.00%, *P* = 0.011) and shorter overall survival (OS) (50.18 ± 6.73 vs. 40.92 ± 16.48 months, *P* = 0.005), while no significant differences in age, sex, microsatellite instability (MSI) status, or tumor stage were observed between the two groups (Table 1). Kaplan‒Meier analysis further confirmed poorer OS in the S100A14-high group (hazard ratio [HR] = 3.62; 95% confidence interval [CI]: 1.36–9.66; *P* = 0.010) (Fig. 2f). These findings were validated in an independent cohort of 1063 primary CRC patients on the basis of transcriptomic data (HR = 1.21, 95% CI: 1.01–1.44; *P* = 0.034) (Fig. 2g). Multivariate Cox regression analysis revealed S100A14 expression as an independent prognostic factor for CRC survival (HR = 4.228, 95% CI: 1.231–15.286; *P* = 0.022) (Table 2).

**Table 1:** Clinical characteristics.

Characteristics	Low (*N* = 35)	High (*N* = 30)	*P* value
Age (year)	55.97 ± 11.78	61.37 ± 13.09	0.087
Sex			0.233
Male	19 (63.33%)	17 (48.57%)	
Female	11 (36.67%)	18 (51.43%)	
MSI status			0.343
MSI-H	4 (16.00%)	7 (26.92%)	
MSS	21 (84.00%)	19 (73.08%)	
Tumor stage			0.757
I	2 (6.67%)	1 (2.86%)	
II	16 (53.33%)	16 (45.71%)	
III	9 (30.00%)	13 (37.14%)	
IV	3 (10.00%)	5 (14.29%)	
Survival status			0.011
Alive	27 (90.00%)	22 (62.86%)	
Dead	3 (10.00%)	13 (37.14%)	
Overall survival time (months)	50.18 ± 6.73	40.92 ± 16.48	0.005
Mean fluorescence intensity of S100A14	5.66 ± 2.02	14.75 ± 4.61	<0.001

**Table 2:** Univariate and multivariate regression analysis of complications.

Risk factors	Univariate analysis		Multivariate analysis	
	OR (95% CI)	*P* value	OR (95% CI)	*P* value
Age (≥60 years/<60 years)	2.402 (0.834–6.919)	0.091	1.768 (0.611–5.114)	0.293
Sex (male/female)	1.023 (0.381–2.748)	0.963		
MSI status (MSI-H/MSS)	0.750 (0.164–3.425)	0.701		
Tumor stage (III-IV/I-II)	6.595 (1.875–23.191)	0.001*	6.256 (1.769–22.122)	0.004*
S100A14 expression (high/low)	4.495 (1.280–15.786)	0.008*	4.228 (1.231–15.286)	0.022*

^*^P-value < 0.05.

Furthermore, correlation analysis between S100A14 expression and CD3^+^/CD8^+^ cell infiltration in CRC samples revealed a significant negative correlation with CD3^+^ (*R* = −0.287, *P* = 0.021) and CD3^+^CD8^+^ cell densities (*R* = −0.263, *P* = 0.034) (Fig. 2e, h, i). Using pancytokeratin (PAN-CK) to demarcate tumor regions, we observed reduced CD3^+^ and CD8^+^ cell infiltration within 0–100 μm of the tumor periphery in the S100A14-high group, although the difference in CD8^+^ cells did not reach statistical significance (Fig. 2j, k). These results demonstrate that S100A14 expression is negatively correlated with immune infiltration in CRC, suggesting its potential role in mediating resistance to anti-PD-1 therapy.

### S100A14 promotes CRC cell malignant phenotypes and inhibits the function of CD8^+^ T cells in vitro

**Fig. 3:**
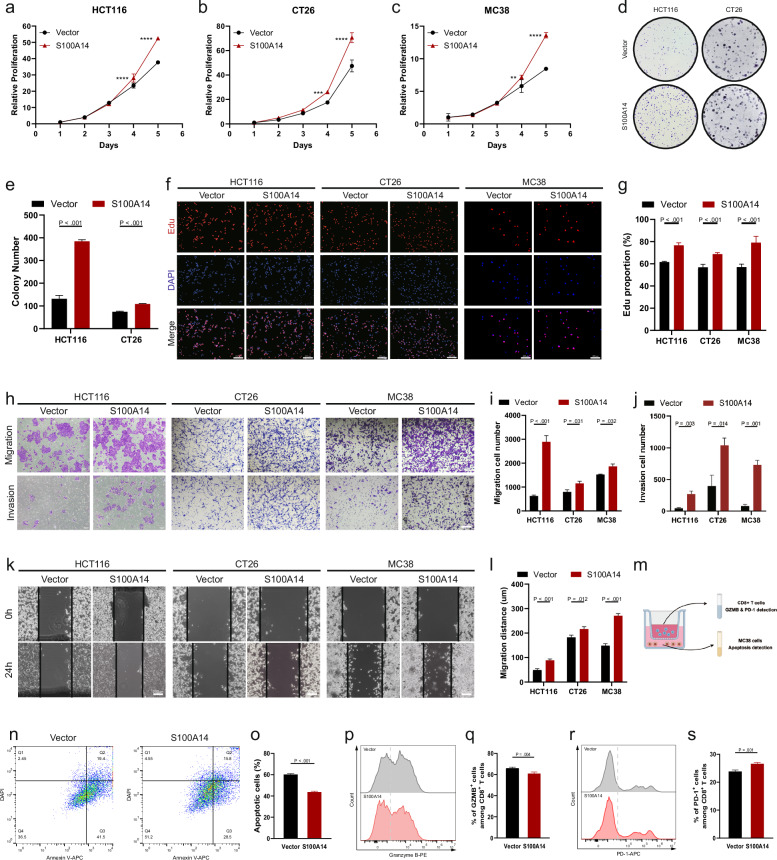
S100A14 promotes CRC cell malignant phenotypes and inhibits the function of CD8^+^ T cells in vitro. **a**–**g** Differences in CRC cell proliferation after S100A14 overexpression, as detected by a CCK-8 assay (**a**–**c**), colony formation assay (**d**, **e**), and EdU assay (scale bar: 200 µm) (**f**, **g**). **h**–**j** Changes in the migration and invasion capacities of HCT116, CT26, and MC38 cells after S100A14 overexpression, as determined by a Transwell assay (scale bar: 200 µm). **k**, **l** Wound healing assay demonstrating the migration and invasion capacities of HCT116, CT26, and MC38 cells after S100A14 overexpression (scale bar: 200 µm). **m** Schematic diagram of the co-culture of MC38 cells with CD8⁺ T cells. MC38 cells were placed in the lower chamber of a Transwell insert, and CD8^+^ T cells were placed in the upper chamber (CD8⁺ T cells : MC38 cells = 5:1). After 24 h of co-culture, MC38 cells were used for apoptosis detection, and CD8⁺ T cells were used for flow cytometric analysis of GZMB and PD-1 expression. **n**, **o** Flow cytometry dot plots (**n**) and statistical analysis (**o**) of apoptosis in MC38 cells after co-culture with CD8⁺ T cells. **p**–**s** Flow cytometry histograms and statistical analysis of GZMB (**p**, **q**) and PD-1 (**r**, **s**) expression in CD8⁺ T cells after co-culture with MC38 cells. The data are presented as the mean ± SD from three independent experiments.

Given the strong clinical correlation between high S100A14 expression, poor prognosis, and reduced immune infiltration, we next investigated whether S100A14 directly drives CRC pathogenesis in vitro. Since the proliferative, migratory, and invasive capacities of cells play pivotal roles in the uncontrolled expansion and metastasis of tumors [16, 17], three cell lines—HCT116, CT26, and MC38—were used to evaluate these malignant phenotypes. CCK-8, colony formation, and EdU (5-ethynyl-2′-deoxyuridine) assays demonstrated that S100A14 overexpression significantly increased the proliferation of CRC cells (Fig. 3a–g). Transwell and wound healing assays further revealed that S100A14 overexpression markedly increased the migratory and invasive abilities of CRC cells (Fig. 3h–l). For the S100A14 knockdown experiments, four shRNA sequences targeting human and murine S100A14 were designed, packaged into lentiviral vectors, and used to infect cells. Western blot (WB) analysis confirmed that shRNA_homo#2 and shRNA_mus#2 exhibited the highest knockdown efficiency and were thus selected for subsequent experiments (Fig. S2). Notably, S100A14 knockdown attenuated the proliferation, migration, and invasion of CRC cells (Fig. S3a–l). These findings collectively support the hypothesis that S100A14 functions as an oncogenic factor, facilitating the initiation and progression of CRC.

Furthermore, using a co-culture system of MC38 cells and murine CD8⁺ T cells, it was found that MC38 cells overexpressing S100A14 exhibited a significantly reduced apoptosis rate in the presence of CD8^+^ T cells (Fig. 3m–o). Flow cytometry analysis revealed that MC38 cells overexpressing S100A14 induced lower expression of GZMB, a key cytotoxic molecule in CD8^+^ T cells (Fig. 3p, q), while promoting higher expression of the exhaustion marker PD-1 in CD8^+^ T cells (Fig. 3r, s). Conversely, knockdown of S100A14 produced the opposite effects (Fig. S3m–s). These data suggest that S100A14 expression in tumor cells exerts an inhibitory effect on CD8^+^ T cell function.

### S100A14 promotes tumor progression and confers anti-PD-1 resistance in vivo

**Fig. 4:**
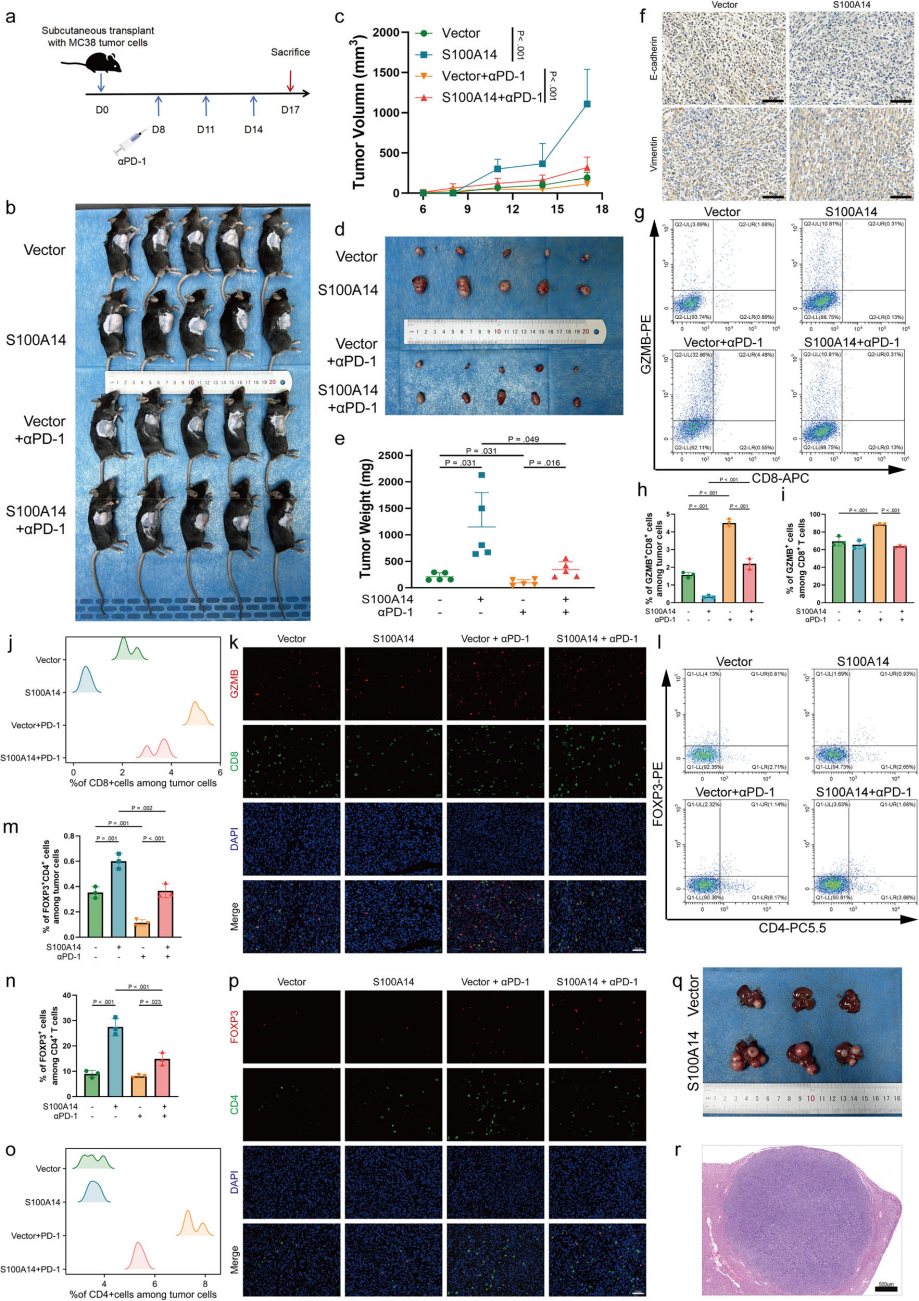
S100A14 promotes tumor progression and confers anti-PD-1 resistance in vivo. **a** Schematic illustration of anti-PD-1 mAb treatment in mice. Mice receiving anti-PD-1 mAb were administered intraperitoneal injections (100 μg/mouse) on Days 8, 11, and 14 after subcutaneous tumor inoculation, followed by euthanasia on Day 17 for subsequent experiments. **b**, **d** Photographs of subcutaneous tumors in mice. **c, e** Growth curves (**c**) and tumor weights (**e**) of subcutaneous tumors. **f** IHC staining for E-cadherin and Vimentin in subcutaneous tumors derived from S100A14-overexpressing and control MC38 cells. **g–j** Flow cytometry (**g**) analysis of the percentages of GZMB^+^ CD8^+^ cells in tumors (**h**), GZMB^+^ cells among CD8^+^ cells (**i**), and CD8^+^ cells in tumors (**j**) between the control and S100A14 overexpression groups (*n* = 3). **k** Representative IF staining images of GZMB^+^ CD8^+^ tumor-infiltrating cells in tumors. Scale bar, 50 µm. **l–o** Flow cytometry (**l**) analysis of the percentages of FOXP3^+^ CD4^+^ cells in tumors (**m**), FOXP3^+^ cells among CD4^+^ cells (**n**), and CD4^+^ cells in tumors (**o**) between the control and S100A14 overexpression groups (*N* = 3). **p** Representative IF staining images of FOXP3^+^ CD4^+^ tumor-infiltrating cells in tumors. Scale bar, 50 µm. **q** Effect of S100A14 overexpression on MC38 cell liver metastasis. **r** H&E-stained images of intrahepatic metastases.

To determine whether the oncogenic and immunosuppressive effects of S100A14 observed in vitro translate to a complex physiological environment, we employed an MC38 syngeneic mouse model to investigate its role in CRC proliferation, migration, invasion, and response to anti-PD-1 therapy in vivo. The treatment schedule for anti-PD-1 therapy is shown in Fig. 4a. Owing to ethical constraints, treatment was limited to three doses of anti-PD-1 because tumors in the untreated group reached the maximum allowed size by Day 17 postimplantation. Compared with the control treatment, S100A14 overexpression significantly accelerated tumor growth, as evidenced by increased tumor volume and weight. Anti-PD-1 therapy induced marked tumor regression in both groups. However, the S100A14-overexpressing group still showed a similar trend of accelerated tumor growth (Fig. 4b–e). Conversely, S100A14 knockdown significantly slowed tumor growth regardless of anti-PD-1 treatment (Fig. S4a–d). Notably, in one mouse with S100A14 knockdown, complete tumor resolution was observed following anti-PD-1 therapy. Meanwhile, consistent with the in vitro experiments, immunohistochemical staining of subcutaneous tumors indicated that S100A14 promoted epithelial–mesenchymal transition (EMT) in CRC (Fig. 4f). Additionally, using a spleen-injected MC38 cell liver metastasis model, we demonstrated that S100A14 promotes hepatic metastasis in immunocompetent mice (Figs. 4q, r and S4o, p). These findings indicate that S100A14 drives CRC progression and confers resistance to anti-PD-1 therapy.

To further elucidate the impact of S100A14 on the CRC immune microenvironment, single-cell suspensions were prepared immediately after tumor resection for flow cytometry analysis. Effector T cells are the primary executors of tumor cell death. Here, we observed a significant negative correlation between CD8^+^ cells (particularly GZMB^+^ CD8^+^ cells) and S100A14 expression in tumor tissues (Figs. 4g–j and S4e–h), which is consistent with our earlier findings. Moreover, compared with the control treatment, S100A14 overexpression increased the proportions of Treg cells (FOXP3^+^ CD4^+^) among tumor cells and FOXP3^+^ cells among CD4^+^ cells, despite a posttreatment decline in both groups (Fig. 4l–n). Although anti-PD-1 therapy increased CD4^+^ cell infiltration in tumors, this increase was attenuated in the S100A14-overexpressing group (Fig. 4o), suggesting that S100A14-mediated CD4^+^ infiltration primarily involves Treg recruitment. Conversely, in the S100A14 knockdown group, the proportions of Treg cells, FOXP3⁺ cells among CD4⁺ cells, and CD4⁺ cells in tumor tissues were significantly lower than those in the control group both before and after treatment (Fig. S4j–m). Immunofluorescence (IF) staining of tumor sections further validated these results (Figs. 4k, p and S4i, n). In summary, S100A14 mediates anti-PD-1 resistance in vivo by suppressing GZMB^+^ CD8^+^ cell infiltration and enhancing Treg recruitment.

### Noncanonical NF-κB activation mediates S100A14-induced aggressiveness and immune evasion

**Fig. 5:**
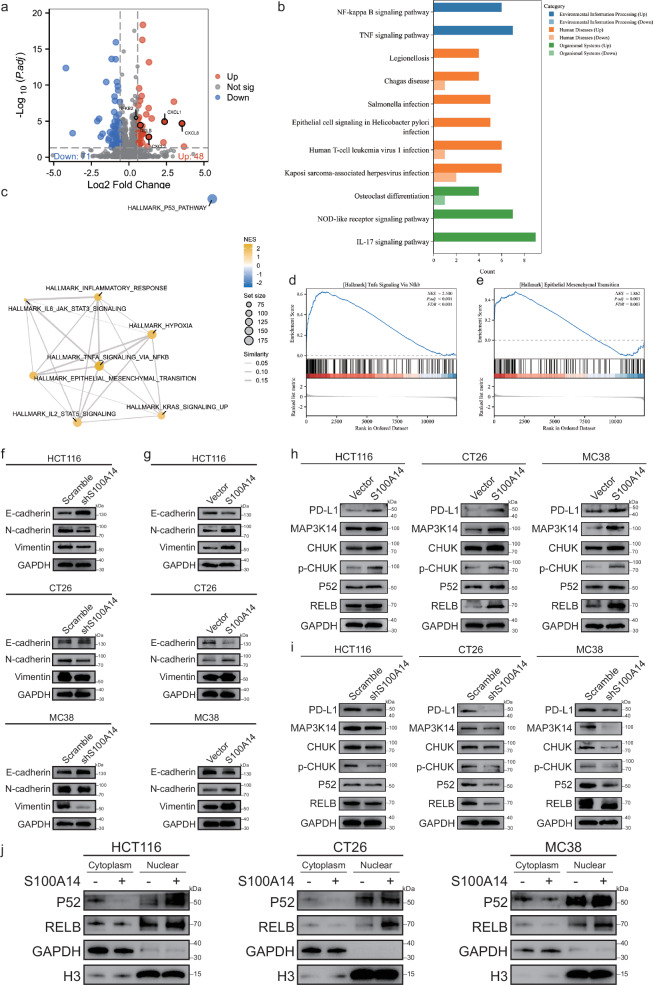
S100A14 upregulates the noncanonical NF-κB cascade, accompanied by EMT and PD-L1 induction. **a** Volcano plot of differentially expressed genes in HCT116 cells overexpressing S100A14 (4 vs 4). **b** KEGG pathway enrichment analysis after S100A14 overexpression. **c** Enrichment analysis of the HALLMARK gene set by GSEA. **d**, **e** GSEA enrichment analysis of EMT-related genes after S100A14 overexpression. **f**, **g** WB analysis demonstrating the role of S100A14 in EMT. **h**, **i** WB analysis of the effects of S100A14 on the expression of noncanonical NF-κB pathway proteins and PD-L1. **j** WB analysis showing that S100A14 overexpression promotes nuclear translocation of the non-canonical NF-κB pathway effector protein.

Having established that S100A14 drives CRC aggressiveness and immune evasion both in vitro and in vivo, we next sought to uncover the underlying molecular mechanisms. To this end, RNA sequencing was performed on four pairs of S100A14-overexpressing HCT116 cells, revealing 48 upregulated genes and 71 downregulated genes (Fig. 5a). Kyoto Encyclopedia of Genes and Genomes (KEGG) enrichment analysis of the differentially expressed genes and gene set enrichment analysis (GSEA) of all the genes revealed significant upregulation of the NF-κB pathway (Fig. 5b, d). Further examination of the expression levels of molecules in the NF-κB pathway indicated that the noncanonical NF-κB pathway was predominantly activated. Specifically, global visualization via a volcano plot highlighted a defined “non-canonical NF-κB–CXCLs signature” within the upregulated fraction (Fig. 5a). While the core upstream transcription factors of this pathway (NFKB2 and RELB) exhibited modest but statistically significant transcriptional upregulation, this initial signal was robustly amplified downstream. This amplification culminated in the profound induction of effector chemokines, including CXCL1, CXCL2, and CXCL8, which function as primary drivers orchestrating Treg recruitment. Thus, we propose that S100A14 primarily functions by upregulating the activity of the noncanonical NF-κB pathway.

Consistent with our previous findings, hallmark EMT genes were significantly enriched upon S100A14 overexpression (Fig. 5e). The GSEA-emap plot revealed similarities between alterations in the NF-κB and EMT pathways following S100A14 overexpression, suggesting that S100A14 may influence EMT via the noncanonical NF-κB pathway (Fig. 5c). Intriguingly, Chen et al. reported that S100A14 mediates EMT by degrading p53 in esophageal squamous cell carcinoma [18], which corroborates our observation of significant downregulation of the p53 pathway upon S100A14 overexpression.

**Fig. 6:**
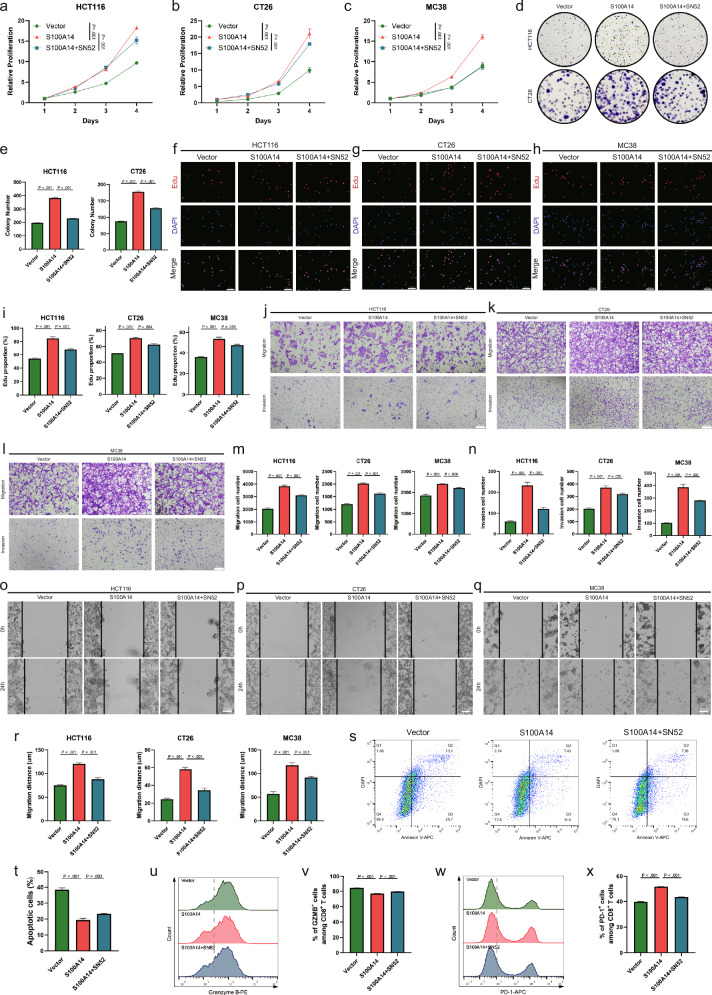
Noncanonical NF-κB activation mediates S100A14-induced CRC aggressiveness and immune evasion. **a–i** CCK-8 (**a**–**c**), colony formation (**d**, **e**), and EdU (**f**–**i**) assays were performed to investigate the effect of SN52 (a specific noncanonical NF-κB inhibitor) (40 μg/ml, treated for 1 h) on S100A14-mediated CRC cell proliferation. **j–r** Transwell (**j**–**n**) and wound-healing (**o**–**r**) assays were conducted to determine the role of the noncanonical NF-κB pathway in S100A14-driven CRC cell migration and invasion. **s, t** Flow cytometry dot plots (**s**) and corresponding quantification (**t**) showing apoptosis of MC38 cells after co-culture with CD8⁺ T cells in the presence or absence of SN52. **u–x** Flow cytometry histograms and corresponding quantification showing the expression of GZMB (**u**, **v**) and PD-1 (**w**, **x**) in CD8⁺ T cells after co-culture with MC38 cells. The data are presented as the mean ± standard deviation (SD) of three independent experiments. Scale bar, 200 µm.

To further validate the reliability of the mRNA sequencing results, WB analysis was conducted to assess the expression levels of noncanonical NF-κB pathway components and EMT signature proteins. As anticipated, S100A14 expression was positively correlated with the expression of noncanonical NF-κB pathway proteins and mesenchymal markers (N-cadherin and vimentin) but negatively correlated with the expression of the epithelial marker E-cadherin (Fig. 5f–i). Nuclear and cytoplasmic protein fractionation followed by WB analysis revealed that S100A14 overexpression led to a significant increase in nuclear NFKB2 and RELB levels, accompanied by a marked decrease in their cytoplasmic levels, further indicating that S100A14 promotes activation of the non-canonical NF-κB pathway (Fig. 5j). Concurrently, PD-L1 expression was found to increase with NF-κB pathway activation, which is consistent with the findings of Zhang et al. that the key effector molecule NFKB2 of the non-canonical NF-κB pathway contributes to immune evasion and metastasis in CRC via a STAT2/PD-L1-dependent mechanism (Fig. 5h, i) [19]. Collectively, the concerted upregulation of PD-L1 and specific CXCL chemokines elegantly bridges the S100A14-driven noncanonical NF-κB activation with the observed immune evasion and aggressive phenotypes. To definitively prove that the noncanonical NF-κB pathway functionally mediates (rather than merely accompanies) S100A14-induced aggressiveness and immune evasion, we performed extensive pharmacological rescue experiments using SN52, a highly specific inhibitor of the noncanonical NF-κB pathway. Crucially, blocking this signaling cascade with SN52 significantly reversed the S100A14-enhanced cell proliferation (Fig. 6a–i), migration, and invasion capabilities (Fig. 6j–r). Furthermore, in CD8^+^ T cell co-culture assays, SN52 treatment effectively abrogated S100A14-induced resistance to T cell-mediated killing and restored T cell cytotoxicity (Fig. 6s–x). These functional rescue data provide direct causal evidence that noncanonical NF-κB hyperactivation is the essential mediator executing S100A14-driven CRC progression and immunosuppression.

### S100A14 activates noncanonical NF-κB by triggering UPF1 ubiquitination and degradation

**Fig. 7:**
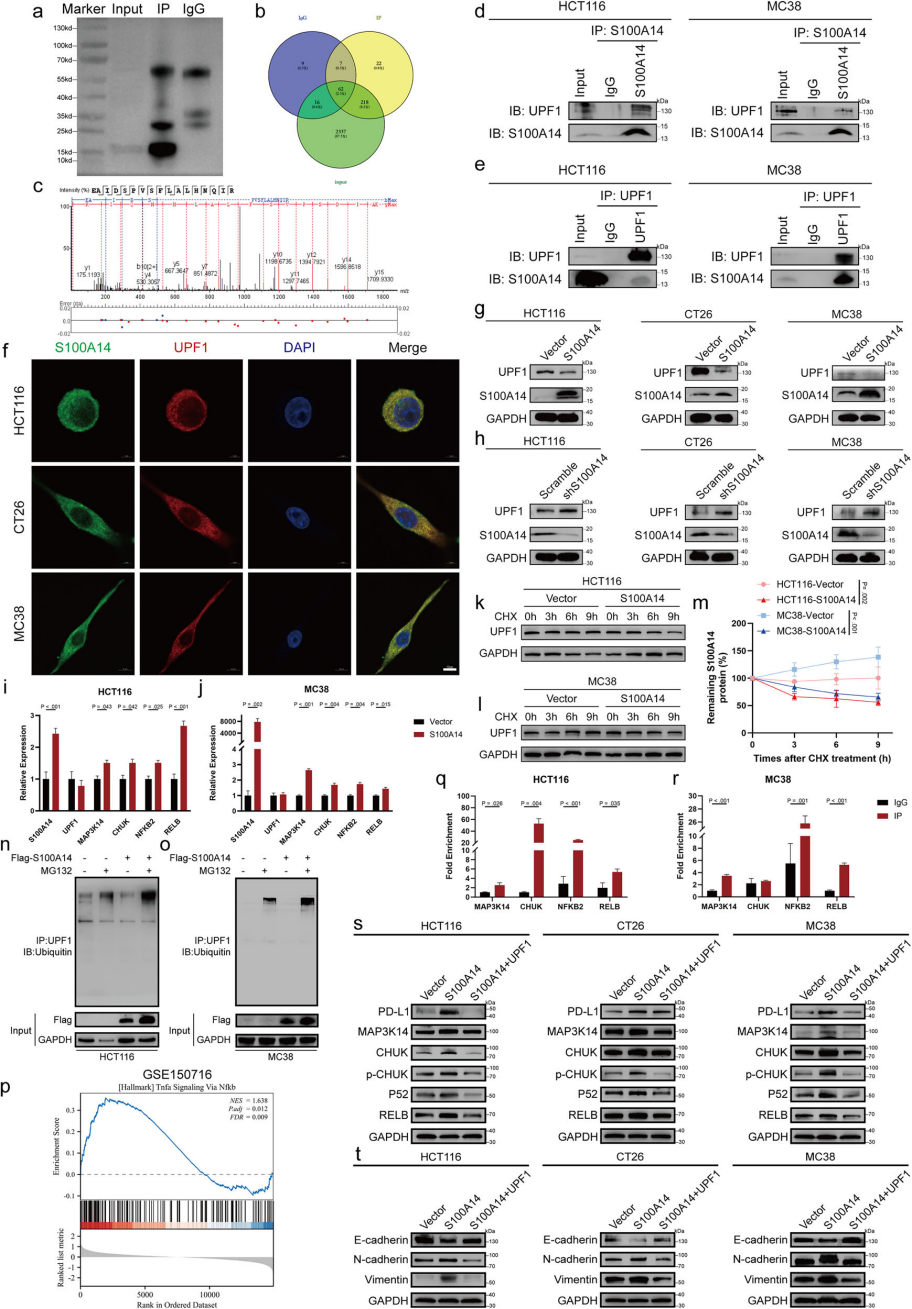
S100A14 activates noncanonical NF-κB signaling by triggering UPF1 ubiquitination and degradation. **a** IP was performed using an anti-FLAG antibody, with IgG serving as the negative control, followed by WB analysis of the immunocomplexes. **b** Mass spectrometry analysis revealed 218 proteins that directly interact with S100A14. **c** Mass spectrum of the UPF1 protein. **d**, **e** HCT116 and MC38 cells were subjected to IP with anti-S100A14 or anti-UPF1 antibodies and subsequently analyzed. **f** IF analysis of S100A14 and UPF1 colocalization in CRC cells. **g**, **h** WB demonstrating the effect of S100A14 on UPF1 expression. **i**, **j** qPCR analysis of gene expression changes in HCT116 and MC38 cells following S100A14 overexpression. **k**–**m** HCT116 and MC38 cells stably transfected with control or S100A14 constructs were treated with CHX (0.1 mg/mL) for the indicated durations, followed by lysate analysis. The percentage of remaining UPF1 protein relative to baseline levels after CHX treatment is shown. **n**, **o** Anti-UPF1 IP was performed to assess the effect of S100A14 on UPF1 ubiquitination. **p** GSEA enrichment analysis of noncanonical NF-κB genes from mRNA-seq data of HCT116 cells following UPF1 knockdown (GSE150716). **q**, **r** RIP‒qPCR analysis of genes capable of binding to UPF1 protein in HCT116 and MC38 cells. **s**, **t** WB analysis of the role of UPF1 in regulating the S100A14-mediated expression of noncanonical NF-κB pathway proteins, EMT-related proteins and PD-L1.

While transcriptomic analysis and WB revealed that S100A14 primarily functions by activating the noncanonical NF-κB pathway, the direct upstream interactors bridging S100A14 to this pathway remained unknown. To elucidate the mechanism by which S100A14 regulates the noncanonical NF-κB pathway, 218 proteins were identified via immunoprecipitation‒mass spectrometry (IP‒MS) as potential S100A14 interactors (Fig. 7a, b). Among these candidates, UPF1 (Fig. 7c) was previously reported to increase nonsense-mediated RNA decay (NMD) of MAP3K14, a key upstream kinase of the noncanonical NF-κB pathway, in inflammatory myofibroblastic tumors [20]. Therefore, UPF1 was selected as a downstream target gene of S100A14. Coimmunoprecipitation (Co-IP) assays confirmed that S100A14 directly binds to UPF1 (Fig. 7d, e), which confirms the reliability of the IP-MS results. Additionally, IF demonstrated the colocalization of S100A14 and UPF1 (Fig. 7f). WB analysis revealed an inverse correlation between S100A14 and UPF1 protein levels (Fig. 7g, h), whereas quantitative PCR (qPCR) revealed no association at the mRNA level (Fig. 7i, j). Given that ubiquitination-mediated protein degradation plays a critical role in diverse biological processes independent of transcriptional or posttranscriptional regulation [21], we hypothesized that S100A14 might regulate UPF1 protein expression via ubiquitination. Treatment with cycloheximide (CHX, 100 µg/mL) to inhibit protein synthesis accelerated the degradation of UPF1 in S100A14-overexpressing cells (Fig. 7k–m). Furthermore, co-IP assays following proteasome inhibition with MG132 revealed significantly increased ubiquitination of UPF1 in S100A14-overexpressing cells (Fig. 7n, o). These findings suggest that S100A14 promotes UPF1 degradation by increasing its ubiquitination.

RNA sequencing data from HCT116 cells with UPF1 knockdown (siUPF1) were retrieved from the GEO database. GSEA of the expression profiles indicated significant upregulation of the NF-κB signaling pathway—particularly the noncanonical NF-κB pathway—upon UPF1 knockdown (Fig. 7p), further supporting the hypothesis that S100A14 upregulates this pathway by promoting UPF1 degradation. The direct binding of UPF1 to mRNA is central to its role in NMD [22]. Here, RNA immunoprecipitation‒qPCR (RIP‒qPCR) assays demonstrated that UPF1 directly binds to mRNAs encoding key noncanonical NF-κB pathway components—MAP3K14, CHUK, NFKB2, and RELB—in CRC cells (Fig. 7q, r). qPCR confirmed elevated mRNA levels of these genes following S100A14 overexpression (Fig. 7i, j), indicating that UPF1 directly degrades their transcripts. Notably, UPF1 expression differed slightly between human HCT116 cells and murine MC38 cells. In MC38 cells, UPF1 did not appear to bind CHUK mRNA directly, yet CHUK mRNA levels still increased upon S100A14 overexpression, suggesting an alternative regulatory mechanism that requires further investigation. Finally, WB analysis revealed that UPF1 overexpression suppressed S100A14-induced alterations in noncanonical NF-κB pathway activity and EMT marker expression (Fig. 7s, t). Collectively, these results demonstrate that S100A14 upregulates the activity of the noncanonical NF-κB pathway by directly binding UPF1 and promoting its ubiquitination-dependent degradation.

### UPF1 is essential for S100A14-mediated CRC progression and immune evasion

**Fig. 8:**
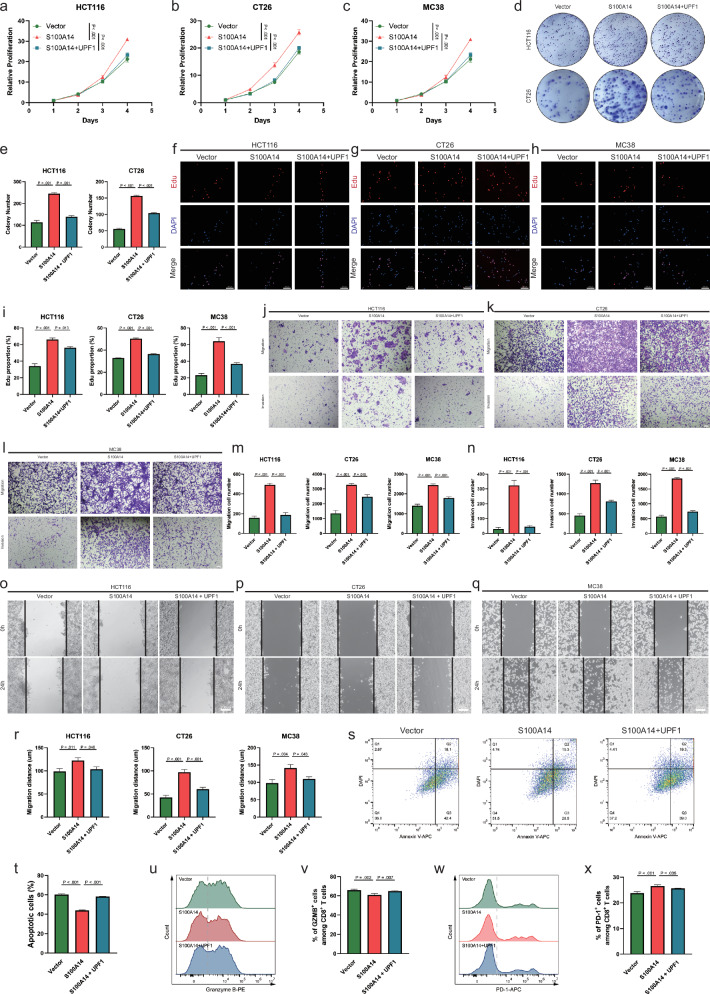
UPF1 is essential for S100A14-mediated CRC progression and immune evasion. **a–i** CCK-8 (**a**–**c**), colony formation (**d**, **e**), and EdU (**f**–**i**) assays were performed to investigate the effect of UPF1 on S100A14-mediated CRC cell proliferation. **j–r** Transwell (**j**–**n**) and wound-healing (**o**–**r**) assays were conducted to determine the role of UPF1 in S100A14-driven CRC cell migration and invasion. **s, t** Flow cytometry dot plots (**s**) and corresponding quantification (**t**) showing apoptosis of MC38 cells after co-culture with CD8⁺ T cells. **u–x** Flow cytometry histograms and corresponding quantification showing the expression of GZMB (**u**, **v**) and PD-1 (**w**, **x**) in CD8⁺ T cells after co-culture with MC38 cells. The data are presented as the mean ± standard deviation (SD) of three independent experiments. Scale bar, 200 µm.

Our mechanistic data demonstrated that S100A14 directly binds to UPF1 and promotes its ubiquitin-dependent degradation. In vitro experiments were subsequently conducted to functionally verify whether S100A14 influences the proliferation, migration, and invasion of CRC cells specifically through the targeting of UPF1. The results of the CCK-8, colony formation, and EdU incorporation assays demonstrated that UPF1 overexpression partially reversed S100A14-induced cell proliferation (Fig. 8a–i). Similarly, the partial reversal of S100A14-enhanced migration and invasion by UPF1 was observed in Transwell and wound-healing assays (Fig. 8j–r). Furthermore, Co-culture of MC38 cells with murine CD8⁺ T cells revealed that UPF1 partially reversed S100A14-induced resistance to CD8⁺ T cell-mediated killing and the induction of T cell exhaustion (Fig. 8s–x).

To further confirm this functional dependency from a loss-of-function perspective, the knockdown efficiency of specific siRNA sequences targeting UPF1 was validated via WB. siUPF1#2 exhibited the most pronounced depletion in human HCT116 and murine CT26 cells, while siUPF1#3 was most effective in murine MC38 cells (Fig. S5a). Subsequently, the functional outcomes were evaluated across a spectrum of genetic contexts, primarily focusing on the comparison between S100A14 deficiency (shS100A14) and the concurrent depletion of both S100A14 and UPF1 (shS100A14 + siUPF1), with Scramble and shS100A14 + NC serving as the respective internal controls. As anticipated, S100A14 deficiency significantly suppressed tumor cell proliferation, migration, and invasion, while also restoring CD8⁺ T cell cytotoxicity. Strikingly, the additional depletion of UPF1 partially reversed these tumor-suppressive effects, effectively reinstating the aggressive and immunosuppressive phenotypes (Fig. S5b–y). In summary, these bidirectional genetic evaluations conclusively demonstrate that UPF1 is the essential downstream mediator for S100A14-driven CRC progression and anti-PD-1 resistance.

## Discussion

Although ICIs demonstrate significant efficacy in patients with MSI-H/dMMR CRC (ORRs reaching 54%), a subset of patients still fail to benefit [23]. Mechanistic studies on ICI resistance in MSI-H/dMMR CRC may facilitate the discovery of novel clinical predictive biomarkers and the development of combined treatment plans to improve therapeutic outcomes. Additionally, these findings may provide new insights into expanding ICI applications to MSS (microsatellite stable)-type CRC, which accounts for 85–95% of cases [24]. In this study, scRNA-seq analysis revealed S100A14 as a critical molecular mediator of anti-PD-1 antibody resistance.

The S100A14 gene is located on chromosome 1q21, and its protein monomer consists of two EF-hand Ca²⁺-binding motifs separated by a flexible hinge region [25]. S100A14 has been reported to be negatively correlated with prognosis in patients with lung, breast, ovarian, and liver cancers [9–12]. In cervical, liver, esophageal squamous cell, and pancreatic cancers, S100A14 promotes tumor cell proliferation and promotes tumor cell migration and invasion via the EMT [12, 18, 26, 27]. In liver and esophageal squamous cell carcinomas, S100A14 has been identified as a marker gene of stem cell subpopulations [28, 29], suggesting its potential role in tumor heterogeneity, drug resistance, and metastatic propensity. Furthermore, in pancreatic, gastric, and endometrial cancers, S100A14 has been recognized as a predictive gene for immunotherapy resistance and is involved in TME regulation [30–32]. However, its prognostic value in CRC remains controversial. Therefore, investigating the mechanistic role of S100A14 and its contribution to immune resistance in CRC is imperative.

Our study revealed that S100A14 is upregulated in CRC compared with adjacent normal tissues and is significantly negatively correlated with prognosis in patients, which is consistent with the results of the largest integrated transcriptome analysis of CRC [33]. Diamantopoulou et al. employed a quantitative scale for more accurate S100A14 expression assessment and similarly reported that high S100A14 expression was associated with shorter DSS and RFS [14]. However, studies using semiquantitative or qualitative methods have reported that low S100A14 expression is correlated with increased malignancy and poor prognosis in patients with CRC [13]. These discrepancies may stem from methodological differences in S100A14 expression evaluation. In our study, compared with single-tissue IHC, TMA-based mIHC minimized intra- and interbatch variability, thereby reducing experimental errors. Moreover, standardized software-based quantitative analysis further mitigated subjective biases from manual interpretation, enhancing the reliability and reproducibility of the results.

Furthermore, in vitro experiments using HCT116, CT26, and MC38 cells revealed positive correlations between S100A14 expression and proliferation, migration, and invasion capabilities. GSEA revealed that the upregulation of EMT signatures following S100A14 overexpression further validated the accuracy of these findings. In vivo murine experiments also confirmed that S100A14 promotes CRC progression. However, Rasool et al. reported that knockdown of S100A14 in human HT29 cells increased proliferation, migration, and invasion [34], which may be attributed to differences in genetic backgrounds among experimental cell lines. Our sequencing results indicated that S100A14 overexpression significantly downregulated p53 signaling, which is consistent with the findings of previous studies. The presence of P53 mutations in HT29 cells may partially alter S100A14 function [35], potentially explaining the discrepancy with the findings of Rasool et al.

Through co-culture experiments, it was found that S100A14 in tumor cells can promote CD8⁺ T cell exhaustion and attenuate their tumor-killing effects. Furthermore, a subcutaneous metastasis model using MC38-derived cells demonstrated that S100A14 acts as a positive regulator of immune evasion in CRC. Flow cytometry and IF analyzes suggested that S100A14 mediates resistance to anti-PD-1 therapy by recruiting more Treg cells and suppressing cytotoxic CD8^+^ cell infiltration. Similarly, mIHC revealed a significant negative correlation between S100A14 expression and CD3^+^/CD8^+^ cell infiltration in CRC. However, Min et al. proposed that compared with chemotherapy alone, S100A14 is a negative regulator of immune evasion in CRC, while the control group demonstrated stronger tumor suppression with combined chemotherapy and anti-PD-1 treatment, and no significant difference was observed between these treatment regimens in CT26 mouse tumors overexpressing S100A14 [36]. Notably, compared with control tumors, S100A14-overexpressing CT26 tumors tended to have increased tumor volume and a decreased proportion of GZMB^+^ cells. These results align with our findings, suggesting that S100A14 promotes CRC progression and anti-PD-1 resistance, contradicting the conclusion that “S100A14 is a novel negative regulator of immune evasion”. Additionally, Min et al. reported that in chemotherapy-resistant HCT116 cells, S100A14 directly binds to STAT3 to increase its ubiquitination-mediated degradation, thereby downregulating PD-L1 expression. These findings contrast with our observation that S100A14 upregulates PD-L1 expression in CRC cells. Importantly, our IP‒MS analysis failed to detect any interaction between S100A14 and STAT3, suggesting potential functional alterations in S100A14 in chemotherapy-resistant CRC cells.

After observing the significant impact of S100A14 on the tumor immune context, we next sought to elucidate the underlying molecular mechanisms responsible for this immunomodulation. Accumulating evidence indicates that the noncanonical NF-κB signaling pathway is involved in immune evasion across multiple cancer types. Zhang Yanyan et al. demonstrated that the noncanonical NF-κB pathway upregulates PD-L1 expression and exacerbates immune evasion in prostate cancer [37]. Yu et al. revealed that activation of the noncanonical NF-κB pathway mediates STAT3-stimulated IDO upregulation in myeloid-derived suppressor cells, significantly suppressing T-cell immunity in breast cancer [38]. Kundu et al. reported that NFKB2-dependent cytokines in triple-negative breast cancer promote neutrophil-mediated inhibition of T-cell proliferation and enhance the immunosuppressive Treg phenotype [39]. Zhang Tianhao et al. reported that the noncanonical NF-κB pathway facilitates immune evasion in gastric cancer by promoting tumor-associated neutrophil recruitment, thereby sensitizing tumors to immune checkpoint blockade therapy [40]. In CRC, substantial evidence suggests that the noncanonical NF-κB pathway plays a pivotal role in promoting tumorigenesis and progression [41–45]. Zhang Jiwei et al. further demonstrated that this pathway mediates immune evasion and metastasis of CRC cells in a STAT2/PD-L1-dependent manner [19]. Through RNA sequencing and WB analysis, our study revealed that S100A14 primarily promotes CRC progression and anti-PD-1 resistance via the noncanonical NF-κB pathway. Notably, our RNA-seq data (visualized via a volcano plot) unveiled a classic “signal amplification” cascade within this axis. While the core upstream transcription factors (e.g., NFKB2 and RELB) exhibited modest but statistically significant transcriptional upregulation, this initial signal was robustly amplified downstream, culminating in the massive induction of specific effector chemokines (CXCL1, CXCL2, and CXCL8). This transcriptomic signature elegantly explains the robust Treg recruitment observed in our phenotypic assays. Most importantly, the ability of SN52 to functionally abrogate the S100A14-driven proliferative, invasive, and immunosuppressive phenotypes provides definitive causal evidence. This confirms that hyperactivation of the noncanonical NF-κB pathway does not merely accompany, but intrinsically mediates, the aggressive progression of CRC.

In this study, we employed IP‒MS and co-IP assays to demonstrate for the first time that S100A14 and UPF1 directly interact. UPF1, a critical factor in the NMD mechanism, maintains gene expression integrity by recognizing premature termination codons, interacting with translation termination factors, and initiating mRNA degradation [22]. UPF1 has been reported to suppress tumorigenesis and progression in multiple cancers [46–51]. In CRC, UPF1 inhibits tumor development and metastasis by suppressing TGF-β/Smad pathway activation and promoting ubiquitin-mediated degradation of the CHD4 and NR4A1 proteins [52–54]. Additionally, in hepatocellular carcinoma, UPF1-mediated downregulation of GPX4 transcript degradation has been implicated in TRIM34-induced resistance to immunotherapy [55]. Notably, Zhu et al. demonstrated that UPF1 overexpression reduces immunogenicity and anti-PD-1 treatment sensitivity in MSI-H CRC, significantly impairing dendritic cell activation and CD8^+^ T-cell immune responses [56]. We therefore hypothesized that S100A14 may promote CRC progression and anti‒PD-1 resistance partly through UPF1. This hypothesis was substantiated by our bidirectional functional evaluations. Specifically, UPF1 overexpression alleviated the CRC progression induced by S100A14, while the concomitant depletion of UPF1 effectively rescued the suppressed aggressive and immunosuppressive phenotypes in S100A14-deficient cells. This consistency observed across both gain- and loss-of-function contexts solidifies UPF1 as the essential downstream mediator in this axis.

Since UPF1 mRNA levels did not significantly increase but its protein levels markedly decreased upon S100A14 overexpression, we speculate that S100A14 is involved in the posttranslational modification (PTM) of UPF1. Ubiquitination is among the most prevalent forms of PTM in eukaryotes and involves the targeting of proteins for degradation via the proteasomal pathway [21]. In nasopharyngeal carcinoma, S100A14 has been reported to promote the degradation of interleukin-1 receptor-associated kinase 1 (IRAK1) through the ubiquitin–proteasome pathway, suggesting that S100A14 may directly or indirectly facilitate ubiquitination [57]. Moreover, UPF1 is degraded via the ubiquitin–proteasome pathway promoted by rotavirus nonstructural protein 5 (NSP5), thereby facilitating viral infection [58]. Using CHX chase assays and ubiquitination experiments, we confirmed that S100A14 promotes the ubiquitination of UPF1, leading to its proteasomal degradation. Additionally, it has been reported that in inflammatory myofibroblastic tumors, UPF1 enhances the expression of NMD of MAP3K14, a key upstream kinase in the noncanonical NF-κB pathway [20]. To investigate whether a similar mechanism exists in CRC, we performed RIP‒qPCR experiments. These results further demonstrated that UPF1 directly mediates the mRNA degradation of MAP3K14, CHUK, NFKB2, and RELB—key components of the noncanonical NF-κB pathway—in CRC. Overexpression of UPF1 partially counteracted the S100A14-induced upregulation of the expression of noncanonical NF-κB pathway proteins and PD-L1. These findings indicate that in CRC, S100A14 directly binds to UPF1, promotes its ubiquitin-dependent degradation, and impairs the UPF1-mediated mRNA decay of core components in the noncanonical NF-κB pathway, thereby activating this pathway to facilitate CRC progression and anti–PD-1 resistance. This mechanism may represent a potential target for tumor-directed therapeutic strategies.

Furthermore, while our study elucidates the intrinsic intracellular S100A14-UPF1 signaling cascade in driving immunosuppression, the tumor-immune microenvironment is also profoundly shaped by extrinsic factors, notably the gut microbiota. Recent extensive reviews have highlighted the critical role of the gut microbiome in broad gastrointestinal cancers [59], particularly in modulating the tumor-immune-microbiome axis [60]. For instance, beyond the local colorectal environment, the gut microbiota and its specific metabolites (such as Equol) can orchestrate systemic anti-tumor immunity and suppress progression in associated gastrointestinal malignancies like hepatocellular carcinoma via the gut-liver axis [61]. Given that harnessing the microbiome is rapidly emerging as a transformative frontier for cancer therapy [62], it is highly intriguing to speculate whether specific gut microbiota profiles in CRC patients might dynamically influence S100A14 expression, or whether S100A14-driven noncanonical NF-κB activation crosstalks with microbiome-derived signals to collectively orchestrate Treg recruitment. Exploring this intrinsic-extrinsic crosstalk represents a promising avenue for our future investigations to further optimize combination therapies.

Beyond these future macroscopic explorations, our current mechanistic findings already hold significant translational value. The identification of S100A14 not only serves as a robust predictive biomarker for identifying CRC patients who are likely to exhibit intrinsic resistance to ICIs, but also uncovers a novel therapeutic vulnerability. Targeting the S100A14-UPF1-noncanonical NF-κB axis holds the potential to effectively reverse the immunosuppressive microenvironment, dampen Treg accumulation, and ultimately restore ICI sensitivity. Therefore, precision combination strategies that couple specific inhibitors of this cascade with PD-1/PD-L1 blockade may represent a rational and promising clinical approach to overcome immunotherapy resistance and improve survival outcomes in patients with aggressive CRC.

This study has several limitations. First, the murine CRC model may not fully recapitulate the tumor–immune interactions observed in human cancers. Future studies should employ orthotopic transplantation or genetically engineered models to validate and extend our findings. Second, the sample size of 65 CRC patients was relatively small. Further expansion of the cohort is warranted to increase the robustness of the outcomes. Finally, the precise mechanisms underlying S100A14-mediated crosstalk between CRC cells and TME components require additional investigation to improve our understanding of immunotherapy resistance in CRC.

In summary, our study elucidates the specific regulatory mechanisms through which S100A14 facilitates CRC progression and confers resistance to PD-1 blockade therapy, underscoring its prognostic value and clinical implications for immunotherapy. The therapeutic strategy combining S100A14 targeting with ICIs shows promising potential for CRC treatment.

## Supplementary information

SUPPLEMENTAL MATERIAL

Uncropped Western Blots

## Data Availability

The expression profile data analyzed in this study are publicly available from the Gene Expression Omnibus (GEO) under accession number GSE205506 and the ArrayExpress Database under accession number E-MTAB-12862. The original mRNA sequencing data generated in this study have been deposited in the figshare repository and are publicly accessible via: 10.6084/m9.figshare.32034906. Other original data generated during this study are available from the corresponding author upon reasonable request. Access to these data will be granted to researchers who provide a methodologically sound proposal for research purposes, subject to the approval of the corresponding author. No additional study-related documents are available. All other supporting data are provided within the article and its supplementary data files.

## References

[1] Bray F, Laversanne M, Sung H, Ferlay J, Siegel RL, Soerjomataram I, et al. Global cancer statistics 2022: GLOBOCAN estimates of incidence and mortality worldwide for 36 cancers in 185 countries. CA Cancer J Clin. 2024;74:229–63. 38572751 10.3322/caac.21834

[2] Keum N, Giovannucci E. Global burden of colorectal cancer: emerging trends, risk factors and prevention strategies. Nat Rev Gastroenterol Hepatol. 2019;16:713–32. 31455888 10.1038/s41575-019-0189-8

[3] Biller LH, Schrag D. Diagnosis and treatment of metastatic colorectal cancer: a review. JAMA. 2021;325:669–85. 33591350 10.1001/jama.2021.0106

[4] Benson AB, Venook AP, Adam M, Chang G, Chen YJ, Ciombor KK, et al. Colon cancer, version 3.2024, NCCN Clinical Practice Guidelines in Oncology. J Natl Compr Canc Netw. 2024;22:e240029. 38862008 10.6004/jnccn.2024.0029

[5] Benson AB, Venook AP, Adam M, Chang G, Chen YJ, Ciombor KK, et al. NCCN Guidelines® Insights: rectal cancer, version 3.2024. J Natl Compr Canc Netw. 2024;22:366–75. 39151454 10.6004/jnccn.2024.0041

[6] Diaz LA Jr, Shiu KK, Kim TW, Jensen BV, Jensen LH, Punt C, et al. Pembrolizumab versus chemotherapy for microsatellite instability-high or mismatch repair-deficient metastatic colorectal cancer (KEYNOTE-177): final analysis of a randomised, open-label, phase 3 study. Lancet Oncol. 2022;23:659–70. 35427471 10.1016/S1470-2045(22)00197-8 PMC9533375

[7] Donato R. S100: a multigenic family of calcium-modulated proteins of the EF-hand type with intracellular and extracellular functional roles. Int J Biochem Cell Biol. 2001;33:637–68. 11390274 10.1016/s1357-2725(01)00046-2

[8] Chen H, Xu C, Jin Q, Liu Z. S100 protein family in human cancer. Am J Cancer Res. 2014;4:89–115. 24660101 PMC3960449

[9] Ding F, Wang D, Li XK, Yang L, Liu HY, Cui W, et al. Overexpression of S100A14 contributes to malignant progression and predicts poor prognosis of lung adenocarcinoma. Thorac Cancer. 2018;9:827–35. 29733545 10.1111/1759-7714.12654 PMC6026614

[10] Al-Ashkar N, Zetoune AB. S100A14 serum level and its correlation with prognostic factors in breast cancer. J Egypt Natl Canc Inst. 2020;32:37. 32984913 10.1186/s43046-020-00048-y PMC13317075

[11] Hu L, Kong F, Pan Y. Prognostic and clinicopathological significance of S100A14 expression in cancer patients: a meta-analysis. Medicine. 2019;98:e16356. 31305429 10.1097/MD.0000000000016356 PMC6641819

[12] Zhao FT, Jia ZS, Yang Q, Song L, Jiang XJ. S100A14 promotes the growth and metastasis of hepatocellular carcinoma. Asian Pac J Cancer Prev. 2013;14:3831–6. 23886191 10.7314/apjcp.2013.14.6.3831

[13] Wang HY, Zhang JY, Cui JT, Tan XH, Li WM, Gu J, et al. Expression status of S100A14 and S100A4 correlates with metastatic potential and clinical outcome in colorectal cancer after surgery. Oncol Rep. 2010;23:45–52. 19956863

[14] Diamantopoulou A, Mantas D, Kostakis ID, Agrogiannis G, Garoufalia Z, Kavantzas N, et al. A clinicopathological analysis of S100A14 expression in colorectal cancer. In Vivo. 2020;34:321–30. 31882495 10.21873/invivo.11777 PMC6984099

[15] Li J, Wu C, Hu H, Qin G, Wu X, Bai F, et al. Remodeling of the immune and stromal cell compartment by PD-1 blockade in mismatch repair-deficient colorectal cancer. Cancer Cell. 2023;41:1152–69.e1157. 37172580 10.1016/j.ccell.2023.04.011

[16] Evan GI, Vousden KH. Proliferation, cell cycle and apoptosis in cancer. Nature. 2001;411:342–8. 11357141 10.1038/35077213

[17] Spano D, Heck C, De Antonellis P, Christofori G, Zollo M. Molecular networks that regulate cancer metastasis. Semin Cancer Biol. 2012;22:234–49. 22484561 10.1016/j.semcancer.2012.03.006

[18] Chen H, Yuan Y, Zhang C, Luo A, Ding F, Ma J, et al. Involvement of S100A14 protein in cell invasion by affecting expression and function of matrix metalloproteinase (MMP)-2 via p53-dependent transcriptional regulation. J Biol Chem. 2012;287:17109–19. 22451655 10.1074/jbc.M111.326975 PMC3366841

[19] Zhang J, Ma F, Li Z, Li Y, Sun X, Song M, et al. NFKB2 mediates colorectal cancer cell immune escape and metastasis in a STAT2/PD-L1-dependent manner. MedComm. 2024;5:e521. 38660687 10.1002/mco2.521 PMC11042535

[20] Lu J, Plank TD, Su F, Shi X, Liu C, Ji Y, et al. The nonsense-mediated RNA decay pathway is disrupted in inflammatory myofibroblastic tumors. J Clin Invest. 2016;126:3058–62. 27348585 10.1172/JCI86508 PMC4966300

[21] Swatek KN, Komander D. Ubiquitin modifications. Cell Res. 2016;26:399–422. 27012465 10.1038/cr.2016.39 PMC4822133

[22] Kurosaki T, Miyoshi K, Myers JR, Maquat LE. NMD-degradome sequencing reveals ribosome-bound intermediates with 3’-end non-templated nucleotides. Nat Struct Mol Biol. 2018;25:940–50. 30275517 10.1038/s41594-018-0132-7 PMC8262411

[23] Ambrosini M, Rousseau B, Manca P, Artz O, Marabelle A, André T, et al. Immune checkpoint inhibitors for POLE or POLD1 proofreading-deficient metastatic colorectal cancer. Ann Oncol. 2024;35:643–55. 38777726 10.1016/j.annonc.2024.03.009

[24] Zhang Y, Rajput A, Jin N, Wang J. Mechanisms of immunosuppression in colorectal cancer. Cancers. 2020;12:3850. 33419310 10.3390/cancers12123850 PMC7766388

[25] Basnet S, Sharma S, DE Costea, Sapkota D. Expression profile and functional role of S100A14 in human cancer. Oncotarget. 2019;10:2996–3012. 31105881 10.18632/oncotarget.26861 PMC6508202

[26] Wang X, Yang J, Qian J, Liu Z, Chen H, Cui Z. S100A14, a mediator of epithelial-mesenchymal transition, regulates proliferation, migration and invasion of human cervical cancer cells. Am J Cancer Res. 2015;5:1484–95. 26101712 PMC4473325

[27] Zhu H, Gao W, Li X, Yu L, Luo D, Liu Y, et al. S100A14 promotes progression and gemcitabine resistance in pancreatic cancer. Pancreatology. 2021;21:589–98. 33579599 10.1016/j.pan.2021.01.011

[28] Ko CH, Cheng CF, Lai CP, Tzu TH, Chiu CW, Lin MW, et al. Differential proteomic analysis of cancer stem cell properties in hepatocellular carcinomas by isobaric tag labeling and mass spectrometry. J Proteome Res. 2013;12:3573–85. 23782096 10.1021/pr4004294

[29] Wong CN, Zhang Y, Ru B, Wang S, Zhou H, Lin J, et al. Identification and characterization of metastasis-initiating cells in ESCC in a multi-timepoint pulmonary metastasis mouse model. Adv Sci. 2024;11:e2401590. 10.1002/advs.202401590 PMC11321633 38864342

[30] Wang C, Chen Y, Xinpeng Y, Xu R, Song J, Ruze R, et al. Construction of immune-related signature and identification of S100A14 determining immune-suppressive microenvironment in pancreatic cancer. BMC Cancer. 2022;22:879. 35953822 10.1186/s12885-022-09927-0 PMC9367131

[31] Che G, Yin J, Wang W, Luo Y, Chen Y, Yu X, et al. Circumventing drug resistance in gastric cancer: a spatial multi-omics exploration of chemo and immuno-therapeutic response dynamics. Drug Resist Updat. 2024;74:101080. 38579635 10.1016/j.drup.2024.101080

[32] Liu J, Chen X, Jiang Y, Cheng W. Development of an immune gene prognostic classifier for survival prediction and respond to immunocheckpoint inhibitor therapy/chemotherapy in endometrial cancer. Int Immunopharmacol. 2020;86:106735. 32619957 10.1016/j.intimp.2020.106735

[33] Nunes L, Li F, Wu M, Luo T, Hammarström K, Torell E, et al. Prognostic genome and transcriptome signatures in colorectal cancers. Nature. 2024;633:137–46. 39112715 10.1038/s41586-024-07769-3 PMC11374687

[34] Rasool S, Hussein Z, Ali H, Al Ismaeel Q, Al-Mahmoodi H, Yalda M, et al. The emerging role of S100A4 and S100A14 proteins in colorectal cancer progression. Cell Mol Biol. 2024;70:134–43. 39707769 10.14715/cmb/2024.70.11.20

[35] Zhang J, Li C, Sun L, Sun D, Zhao T. P53‑microRNA interactions regulate the response of colorectal tumor cells to oxaliplatin under normoxic and hypoxic conditions. Oncol Rep. 2023;50:219. 37921068 10.3892/or.2023.8656 PMC10636723

[36] Min HY, Cho J, Sim JY, Boo HJ, Lee JS, Lee SB, et al. S100A14: A novel negative regulator of cancer stemness and immune evasion by inhibiting STAT3-mediated programmed death-ligand 1 expression in colorectal cancer. Clin Transl Med. 2022;12:e986. 35858011 10.1002/ctm2.986 PMC9299575

[37] Zhang Y, Zhu S, Du Y, Xu F, Sun W, Xu Z, et al. RelB upregulates PD-L1 and exacerbates prostate cancer immune evasion. J Exp Clin Cancer Res. 2022;41:66. 35177112 10.1186/s13046-022-02243-2 PMC8851785

[38] Yu J, Wang Y, Yan F, Zhang P, Li H, Zhao H, et al. Noncanonical NF-κB activation mediates STAT3-stimulated IDO upregulation in myeloid-derived suppressor cells in breast cancer. J Immunol. 2014;193:2574–86. 25063873 10.4049/jimmunol.1400833 PMC4719564

[39] Kundu M, Greer YE, Lobanov A, Ridnour L, Donahue RN, Ng Y, et al. TRAIL induces cytokine production via the NFkB2 pathway promoting neutrophil chemotaxis and neutrophil-mediated immune-suppression in triple negative breast cancer cells. Cancer Lett. 2025;620:217692. 40187604 10.1016/j.canlet.2025.217692 PMC12049148

[40] Zhang T, Li Y, Zhai E, Zhao R, Qian Y, Huang Z, et al. Intratumoral Fusobacterium nucleatum recruits tumor-associated neutrophils to promote gastric cancer progression and immune evasion. Cancer Res. 2025;85:1819–41. 39992708 10.1158/0008-5472.CAN-24-2580 PMC12079103

[41] Shi W, Ye Z, Zhuang L, Li Y, Shuai W, Zuo Z, et al. Olfactomedin 1 negatively regulates NF-κB signalling and suppresses the growth and metastasis of colorectal cancer cells. J Pathol. 2016;240:352–65. 27555280 10.1002/path.4784

[42] Tao Y, Liu Z, Hou Y, Wang S, Liu S, Jiang Y, et al. Alternative NF-κB signaling promotes colorectal tumorigenesis through transcriptionally upregulating Bcl-3. Oncogene. 2018;37:5887–900. 29973688 10.1038/s41388-018-0363-4

[43] Zhou X, Shan Z, Yang H, Xu J, Li W, Guo F. RelB plays an oncogenic role and conveys chemo-resistance to DLD-1 colon cancer cells. Cancer Cell Int. 2018;18:181. 30473630 10.1186/s12935-018-0677-x PMC6234565

[44] Burkitt MD, Hanedi AF, Duckworth CA, Williams JM, Tang JM, O’Reilly LA, et al. NF-κB1, NF-κB2 and c-Rel differentially regulate susceptibility to colitis-associated adenoma development in C57BL/6 mice. J Pathol. 2015;236:326–36. 25727407 10.1002/path.4527 PMC4737252

[45] Moy RH, Nguyen A, Loo JM, Yamaguchi N, Kajba CM, Santhanam B, et al. Functional genetic screen identifies ITPR3/calcium/RELB axis as a driver of colorectal cancer metastatic liver colonization. Dev Cell. 2022;57:1146–59.e1147. 35487218 10.1016/j.devcel.2022.04.010 PMC9446818

[46] Li Y, Hu J, Guo D, Ma W, Zhang X, Zhang Z, et al. LncRNA SNHG5 promotes the proliferation and cancer stem cell-like properties of HCC by regulating UPF1 and Wnt-signaling pathway. Cancer Gene Ther. 2022;29:1373–83. 35338348 10.1038/s41417-022-00456-3 PMC9576592

[47] Liu S, Chen W, Hu H, Zhang T, Wu T, Li X, et al. Long noncoding RNA PVT1 promotes breast cancer proliferation and metastasis by binding miR-128-3p and UPF1. Breast Cancer Res. 2021;23:115. 34922601 10.1186/s13058-021-01491-y PMC8684126

[48] Abudourexiti G, Zhang J, Abuduxikuer G. UPF1 inhibits cervical cancer cell migration and invasion by regulating nonsense-mediated decay of BMP6 and lncRNA WAKMAR2. J Gynecol Oncol. 2025;36:e127. 40613114 10.3802/jgo.2025.36.e127 PMC12636110

[49] Pei CL, Fei KL, Yuan XY, Gong XJ. LncRNA DANCR aggravates the progression of ovarian cancer by downregulating UPF1. Eur Rev Med Pharmacol Sci. 2019;23:10657–63. 31858532 10.26355/eurrev_201912_19763

[50] Zhou Y, Li Y, Wang N, Li X, Zheng J, Ge L. UPF1 inhibits the hepatocellular carcinoma progression by targeting long non-coding RNA UCA1. Sci Rep. 2019;9:6652. 31040354 10.1038/s41598-019-43148-z PMC6491801

[51] Li L, Geng Y, Feng R, Zhu Q, Miao B, Cao J, et al. The human RNA surveillance factor UPF1 modulates gastric cancer progression by targeting long non-coding RNA MALAT1. Cell Physiol Biochem. 2017;42:2194–206. 28942451 10.1159/000479994

[52] Xie X, Lin J, Liu J, Huang M, Zhong Y, Liang B, et al. A novel lncRNA NR4A1AS up-regulates orphan nuclear receptor NR4A1 expression by blocking UPF1-mediated mRNA destabilization in colorectal cancer. Clin Sci. 2019;133:1457–73. 10.1042/CS20181061 31253658

[53] Wang X, Lai Q, He J, Li Q, Ding J, Lan Z, et al. LncRNA SNHG6 promotes proliferation, invasion and migration in colorectal cancer cells by activating TGF-β/Smad signaling pathway via targeting UPF1 and inducing EMT via regulation of ZEB1. Int J Med Sci. 2019;16:51–59. 30662328 10.7150/ijms.27359 PMC6332483

[54] Luo J, Xu S, Wang J, He L, Li Z. Circular RNA circWBSCR22 facilitates colorectal cancer metastasis by enhancing CHD4’s protein stability. Int J Biol Macromol. 2024;282:137135. 39486700 10.1016/j.ijbiomac.2024.137135

[55] Yao F, Zhou S, Zhang R, Chen Y, Huang W, Yu K, et al. CRISPR/Cas9 screen reveals that targeting TRIM34 enhances ferroptosis sensitivity and augments immunotherapy efficacy in hepatocellular carcinoma. Cancer Lett. 2024;593:216935. 38704136 10.1016/j.canlet.2024.216935

[56] Zhu S, Bai Y, Zhang D, Huang C, Qian X, Li P, et al. Inhibiting UPF1 methylation enhances tumor immunotherapy sensitivity by reducing nonsense-mediated mRNA decay. Cell Rep. 2025;44:115919. 40570371 10.1016/j.celrep.2025.115919

[57] Meng DF, Sun R, Liu GY, Peng LX, Zheng LS, Xie P, et al. S100A14 suppresses metastasis of nasopharyngeal carcinoma by inhibition of NF-kB signaling through degradation of IRAK1. Oncogene. 2020;39:5307–22. 32555330 10.1038/s41388-020-1363-8

[58] Sarkar R, Banerjee S, Mukherjee A, Chawla-Sarkar M. Rotaviral nonstructural protein 5 (NSP5) promotes proteasomal degradation of up-frameshift protein 1 (UPF1), a principal mediator of nonsense-mediated mRNA decay (NMD) pathway, to facilitate infection. Cell Signal. 2022;89:110180. 34718106 10.1016/j.cellsig.2021.110180

[59] Ağagündüz D, Cocozza E, Cemali Ö, Bayazıt AD, Nanì MF, Cerqua I, et al. Understanding the role of the gut microbiome in gastrointestinal cancer: A review. Front Pharmacol. 2023;14:1130562. 36762108 10.3389/fphar.2023.1130562 PMC9903080

[60] Yang Z, Zhou M, Luo F, Feng S, Tan Y, Wang Q, et al. Cryptotanshinone targets tumor-immune-microbiome axis to suppress colorectal cancer. Front Pharmacol. 2025;16:1667500. 41446797 10.3389/fphar.2025.1667500 PMC12722906

[61] Wei X, Huang H, Wang F, Tan P, Wang Z, Qiu X, et al. Modulation of gut microbiota and its metabolite Equol by Huaier granule suppresses hepatocellular carcinoma via the gut-liver axis. NPJ Biofilms Microbiomes. 2026;12:54. 41588026 10.1038/s41522-026-00919-7 PMC12946347

[62] Hajjar R, Mars RAT, Kashyap PC. Harnessing the microbiome for cancer therapy. Nat Rev Microbiol. 2026;24:392–407. 41486395 10.1038/s41579-025-01268-6 PMC12914569

