# Supplementary material for: S100A14 promotes colorectal cancer progression and anti-PD-1 resistance via UPF1-mediated activation of the non-canonical NF-κB signaling
Source: Cell Death Dis. 2026 Jun 25;17(1):597. doi: 10.1038/s41419-026-09032-1 (PMC13303824; doi:10.1038/s41419-026-09032-1)
Supplement: Supplementary file 1 — SUPPLEMENTAL MATERIAL [file 41419_2026_9032_MOESM1_ESM.docx]

**Supplementary Figures**

**
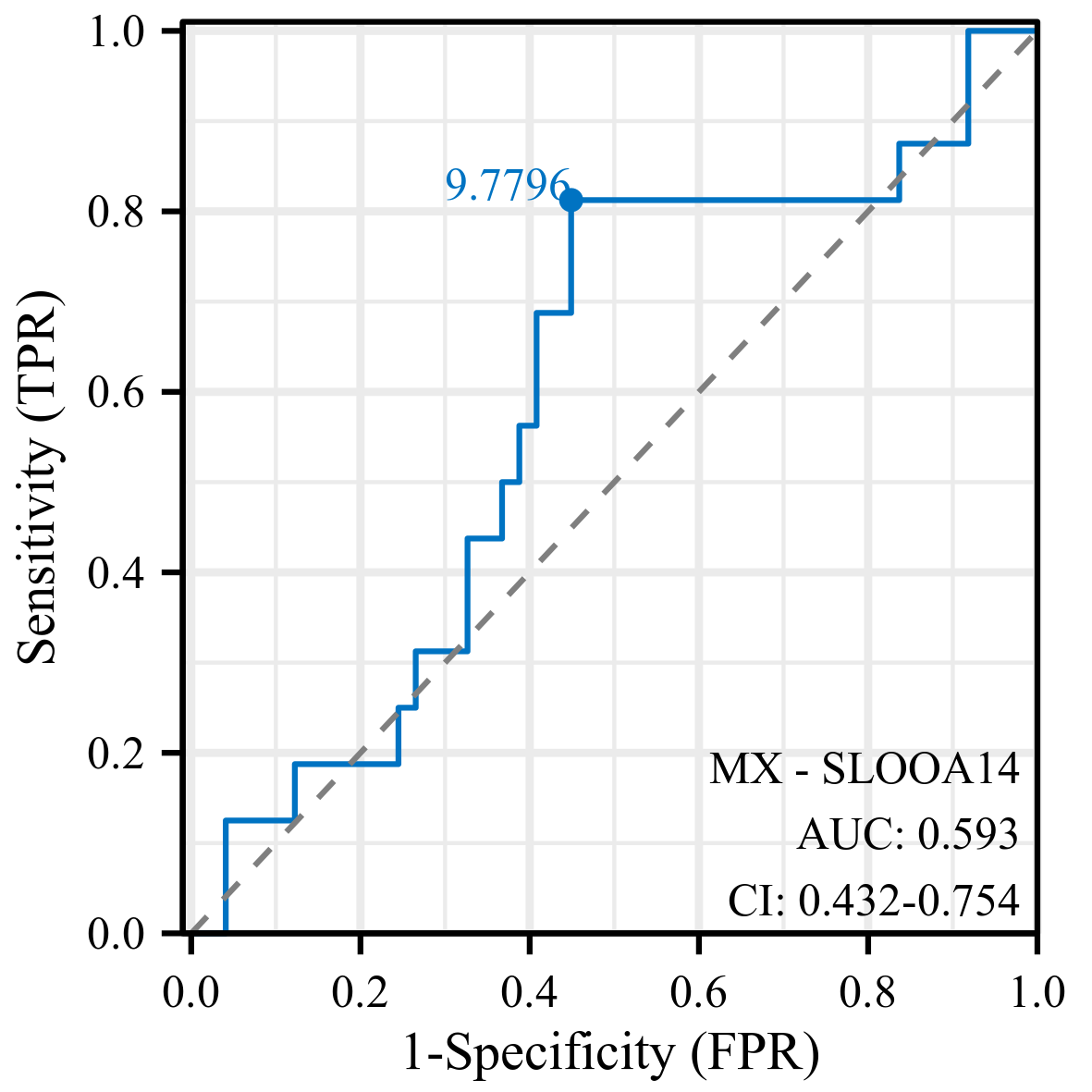
**

***Fig. S1.*** ROC curve for survival prediction in CRC patients.


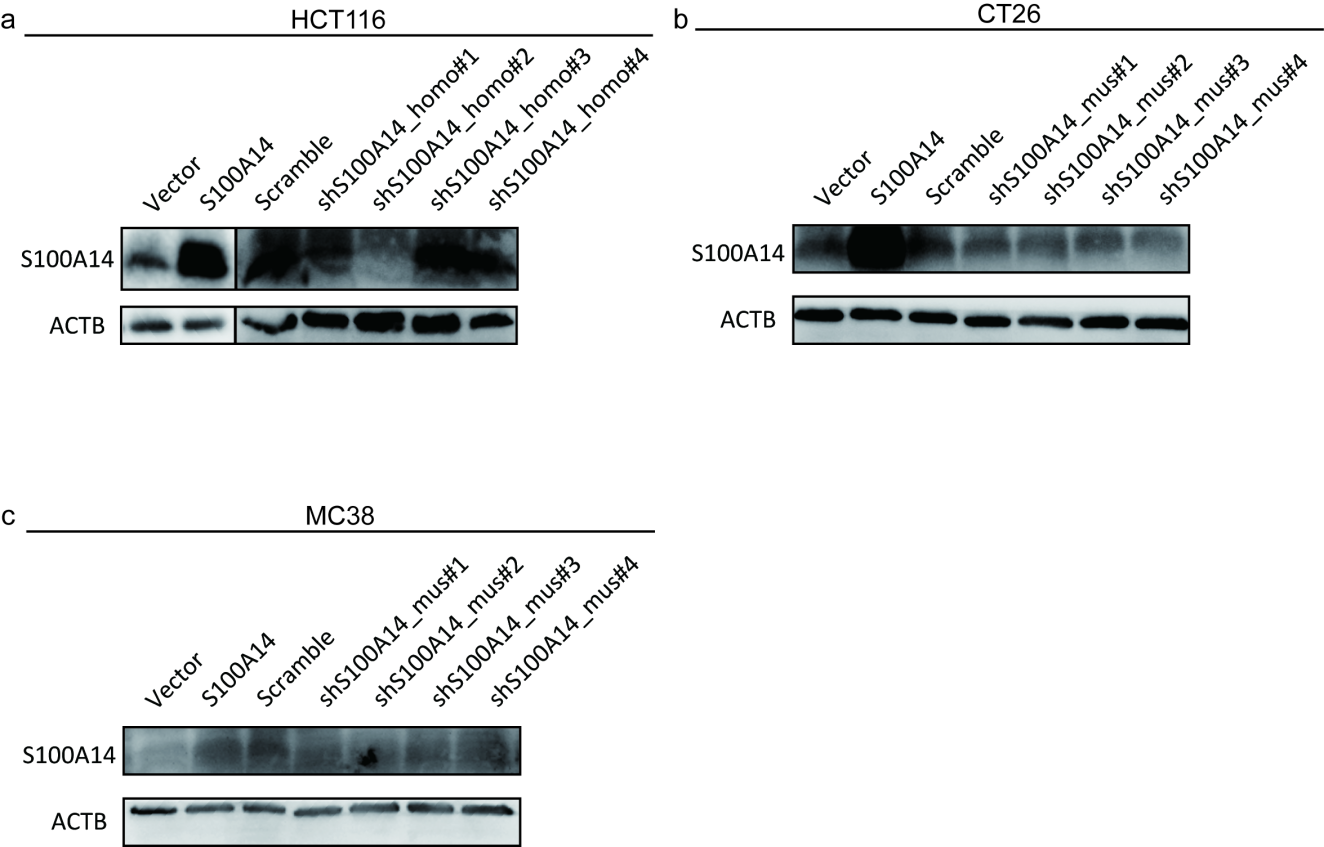

***Fig. S2.*** Validation of S100A14 overexpression and knockdown efficiency in HCT116 (**a**), CT26 (**b**), and MC38 (**c**) cells.


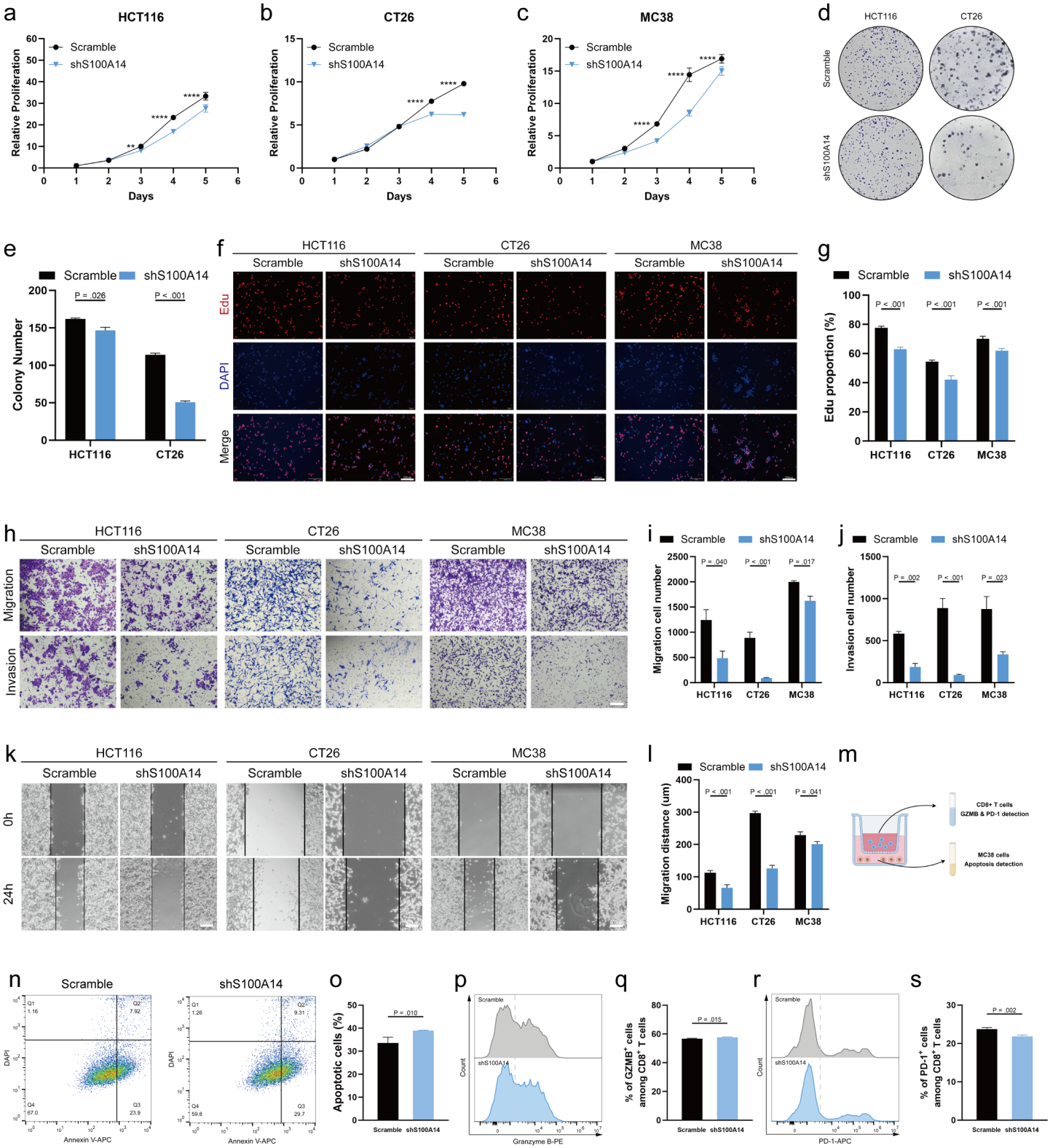


***Fig. S3.*** S100A14 promotes CRC cell malignant phenotypes and inhibits the function of CD8⁺ T cells in vitro. (**a-g**) Differences in CRC cell proliferation after S100A14 knockdown, as detected by a CCK-8 assay (**a-c**), colony formation (**d, e**), and EdU assays (**f, g**). (**h-j**) Changes in the migration and invasion capacities of HCT116, CT26, and MC38 cells after S100A14 knockdown, as determined by a Transwell assay (scale bar: 200 µm). (**k, l**) Wound healing assay demonstrating the migration and invasion capacities of HCT116, CT26, and MC38 cells after S100A14 knockdown(scale bar: 200 µm). (**m**) Schematic diagram of the co-culture of MC38 cells with CD8⁺ T cells. MC38 cells were placed in the lower chamber of a Transwell insert, and CD8⁺ T cells were placed in the upper chamber (CD8⁺ T cells : MC38 cells = 5:1). After 24 hours of co-culture, MC38 cells were used for apoptosis detection, and CD8⁺ T cells were used for flow cytometric analysis of GZMB and PD-1 expression. (**n, o**) Flow cytometry dot plots (**n**) and statistical analysis (**o**) of apoptosis in MC38 cells after co-culture with CD8⁺ T cells. (**p–s**) Flow cytometry histograms and statistical analysis of GZMB (**p, q**) and PD-1 (**r, s**) expression in CD8⁺ T cells after co-culture with MC38 cells. The data are presented as the mean ± SD from three independent experiments.


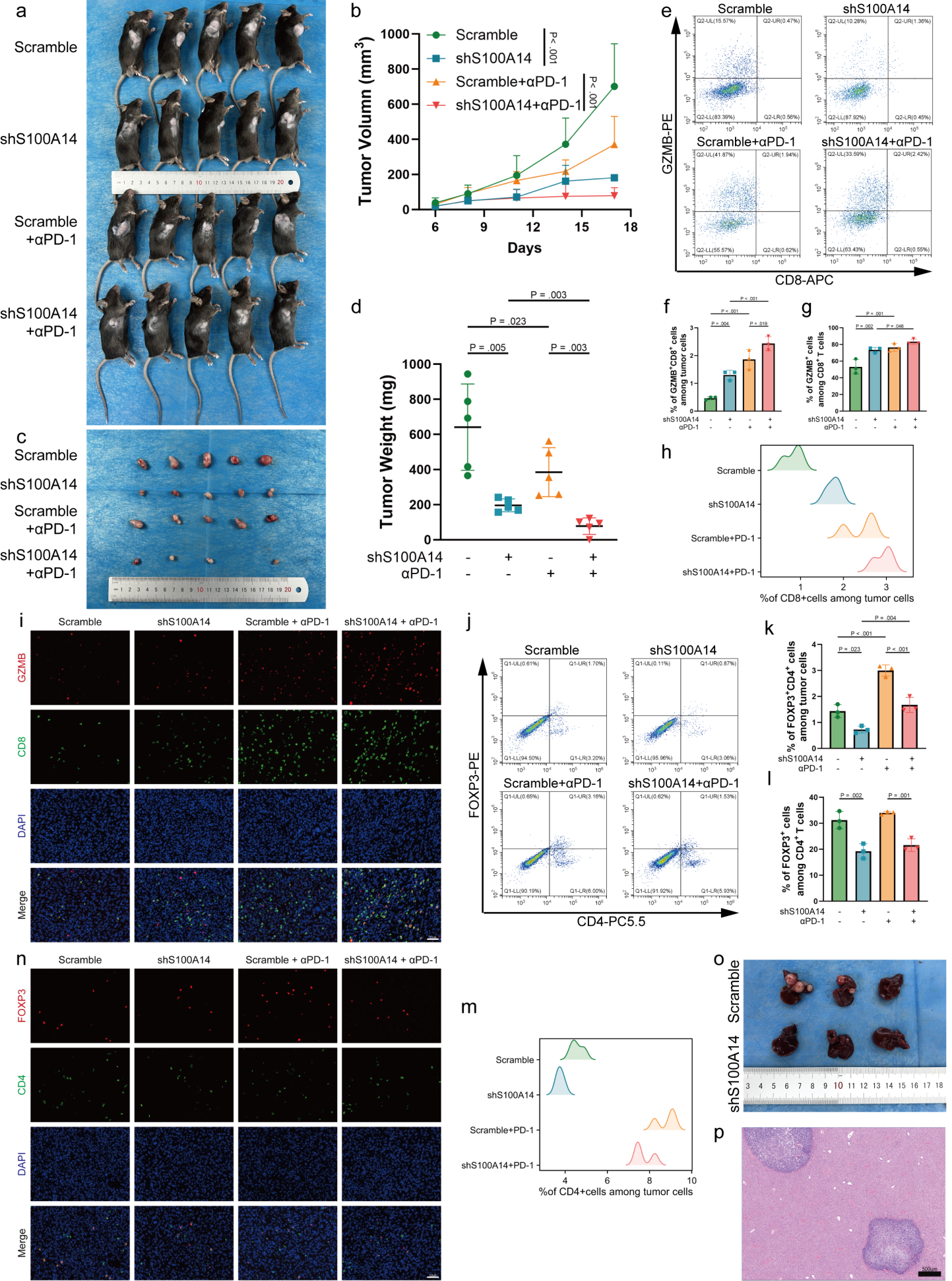


***Fig. S4.*** S100A14 promotes tumor progression and confers anti-PD-1 resistance. (**a, c**) Photographs of subcutaneous tumors in mice. (**b, d**) Growth curves (**b**) and tumor weights (**d**) of subcutaneous tumors. (**e-h**) Flow cytometry (**e**) analysis of the percentages of GZMB+CD8+ cells in tumors (**f**), GZMB+ cells among CD8+ cells (**g**), and CD8+ cells in tumors (**h**) between the control and S100A14 knockdown groups (n=3). (**i**) Representative IF staining images of GZMB+CD8+ tumor-infiltrating cells in tumors. Scale bar, 50 µm. (**j-m**) Flow cytometry (**j**) analysis of the percentages of FOXP3+ CD4+ cells in tumors (**k**), FOXP3+ cells among CD4+ cells (**l**), and CD4+ cells in tumors (**m**) between the control and S100A14 knockdown groups (N=3). (**n**) Representative IF staining images of FOXP3+ CD4+ tumor-infiltrating cells in tumors. Scale bar, 50 µm. (**o**) Effect of S100A14 knockdown on MC38 cell liver metastasis. (**p**) H&E-stained images of intrahepatic metastases.


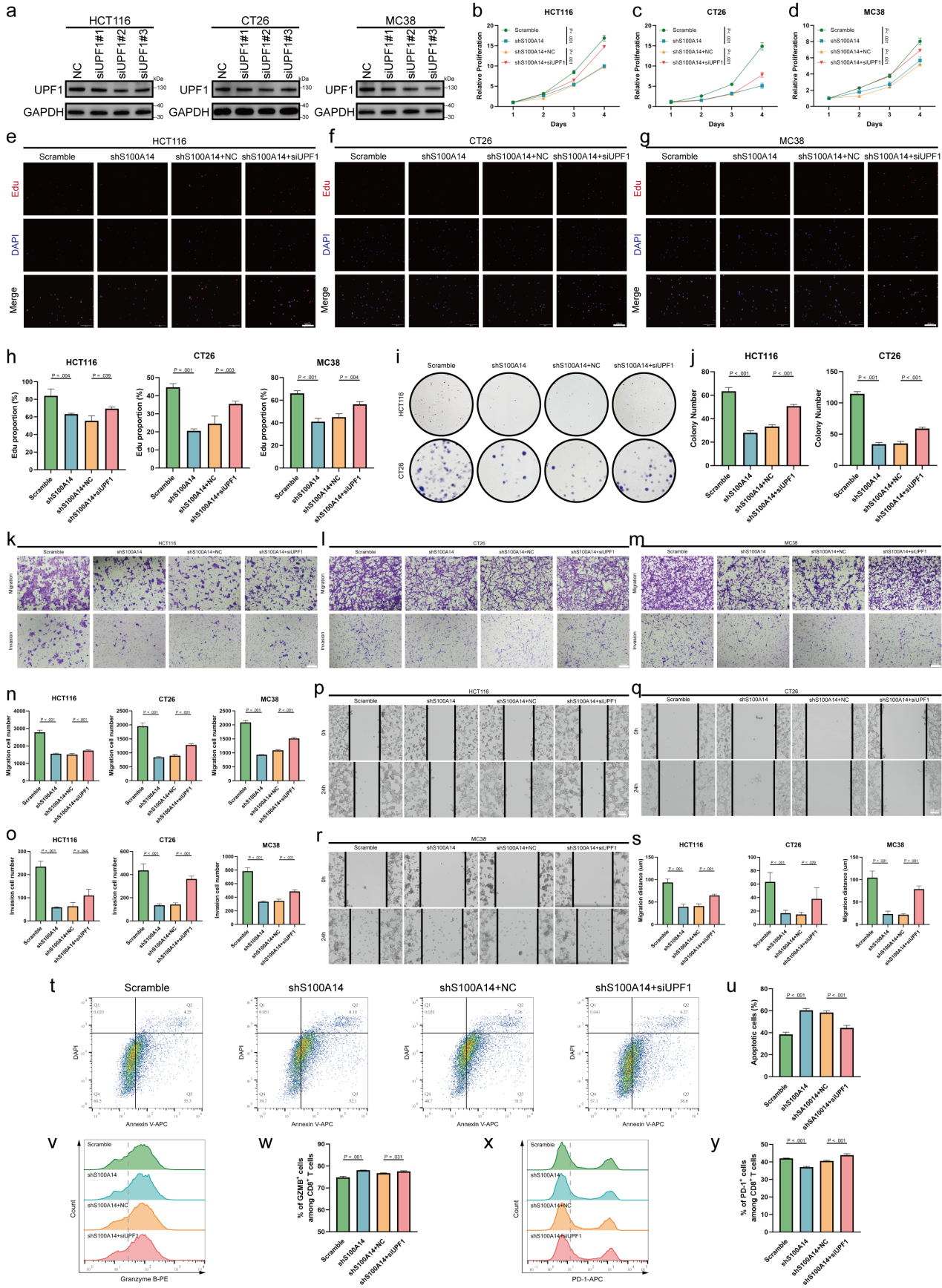


***Fig. S5.*** UPF1 is essential for S100A14-mediated CRC progression and immune evasion. **(a)** Western blot analysis of UPF1 protein levels in human HCT116, murine CT26, and MC38 cells transfected with different siRNA sequences (siUPF1#1, #2, and #3) or a Scramble control. siUPF1#2 was identified as the most effective sequence for HCT116 and CT26 cells, while siUPF1#3 was selected for MC38 cells. **(b-s)** CCK-8 **(b-d)**, EdU **(e-h)**, colony formation **(i, j)**, Transwell **(k-o)**, and Wound healing **(p-s)** assays were conducted to evaluate the effect of concurrent UPF1 depletion on shS100A14-induced suppression of cell proliferation and migration. **(t-y)** Flow cytometry was performed to evaluate MC38 apoptosis (t, u), T cell cytotoxicity (GZMB) **(v, w)**, and exhaustion (PD-1) **(x, y)** in co-cultures, confirming the reversal of shS100A14-restored T cell function upon UPF1 depletion. The data are presented as the mean ± standard deviation (SD) of three independent experiments. Scale bar, 200 µm.

**Supplementary Tables**

**Table*. S1.*** ACT_Annotation results_ACT_Annotation results.

|  | **Cluster** | **Cell.Type** | **Padj** | **whg.p** | **gsea.p** | **Over.Markers.whg** | **Over.Markers.gsea** | **whg.overlap** | **gsea.overlap** |
| --- | --- | --- | --- | --- | --- | --- | --- | --- | --- |
| B cell | cluster0 | Follicular B cell | 3.22549256079119e-61 | 3.41321964104891e-63 | NA | 13/27 | 24/163 | MS4A1,CD79A,CD37,CD79B,TCL1A,IGHD,LTB,CD22,CD74,CD52,HLA-DRA,HLA-DPB1,FCER2 | MS4A1,CD79A,BANK1,VPREB3,CD37,CD79B,TCL1A,IGHD,TNFRSF13C,LTB,SMIM14,IGHM,POU2F2,LINC00926,CD22,HLA-DQB1,CD74,CD52,HLA-DRA,HLA-DPB1,FCER2,CD19,MEF2C,FCRLA |
|  | cluster5 | Plasma cell | 1.23834339321824e-128 | 6.55208144559932e-131 | NA | 19/64 | 29/342 | IGHA1,IGHA2,IGKC,JCHAIN,IGLC2,IGHG1,IGLC3,IGHG4,IGHG3,IGHG2,IGLL5,MZB1,XBP1,SSR4,DERL3,TNFRSF17,FKBP11,SEC11C,PRDX4 | IGHA1,IGHA2,IGKC,JCHAIN,IGLC2,IGHG1,IGLC3,IGHG4,IGHG3,IGHG2,IGLL5,MZB1,XBP1,SSR4,DERL3,HERPUD1,BTG2,TNFRSF17,FKBP11,SEC11C,HSP90B1,TENT5C,PRDX4,FCRL5,PDK1,PIM2,SSR3,FKBP2,TP53INP1 |
|  | cluster14 | Follicular B cell | 1.33143981994992e-39 | 1.40893102640204e-41 | NA | 7/27 | 17/163 | MS4A1,CD79B,TCL1A,CD79A,CD22,CD37,LTB | MS4A1,CD79B,TCL1A,CD79A,SMIM14,VPREB3,CD22,CD37,POU2F2,POU2AF1,LTB,TNFRSF13C,FCRLA,SPIB,CD19,HLA-DOB,LIMD2 |
| T/I/NK cell | cluster1 | CD8-positive, alpha-beta T cell | 1.50264962807506e-55 | 1.9389027459033e-56 | NA | 16/96 | 29/495 | CCL5,GZMK,NKG7,GZMA,CCL4,CD8A,GZMH,CD8B,IFNG,GZMB,CD2,TRAC,CD69,TRBC2,DUSP2,GNLY | CCL5,GZMK,NKG7,GZMA,CCL4,CCL4L2,CD8A,CST7,GZMH,CD8B,CD3D,IFNG,HCST,CTSW,TRGC2,GZMB,CD2,CD3E,TRAC,CD69,TRBC2,CD3G,IL32,KLRD1,GZMM,DUSP2,GNLY,EVL,CD7 |
|  | cluster3 | CD4-positive, alpha-beta T cell | 5.11111948475668e-26 | 3.2974964417785e-27 | NA | 9/93 | 29/499 | IL7R,KLRB1,TRBC1,TRAC,CD2,TCF7,CD40LG,LTB,PTPRC | IL7R,KLRB1,TRBC1,TRAC,FYB1,TRBC2,CD3D,CD2,CD3G,CD3E,TCF7,CD40LG,SPOCK2,PIK3IP1,RORA,LTB,LAT,TRAF3IP3,SARAF,TSC22D3,BCL11B,GIMAP7,EVL,PTPRC,BTG1,CD48,GIMAP4,LCK,RPS27 |
|  | cluster9 | Regulatory T cell | 1.27581262514363e-67 | 4.11552459723756e-69 | NA | 16/68 | 28/222 | TNFRSF4,CTLA4,TNFRSF18,IL32,BATF,TIGIT,TRAC,TRBC1,TRBC2,IL2RA,CD7,ICOS,CD27,FOXP3,DUSP4,RTKN2 | TNFRSF4,CTLA4,TNFRSF18,IL32,BATF,TIGIT,TRAC,TRBC1,SPOCK2,LINC01943,CD2,TRBC2,RGS1,IL2RA,CD7,CD3D,TNFRSF1B,S100A4,LTB,ICOS,MAF,CD27,FOXP3,DUSP4,SRGN,IL2RG,RORA,RTKN2 |
|  | cluster11 | Natural killer cell | 8.83196892295683e-103 | 4.67299943013587e-105 | NA | 24/150 | 28/433 | GNLY,TRDC,KLRD1,CCL5,NKG7,GZMA,CTSW,KLRC1,GZMB,CD7,XCL2,PRF1,TRGC2,CD247,CST7,CCL4,XCL1,GZMH,TRGC1,IL32,IL2RB,CD160,CD96,ITGA1 | GNLY,TRDC,KLRD1,CCL5,NKG7,GZMA,KLRC2,CTSW,KLRC1,HOPX,GZMB,CD7,XCL2,PRF1,TRGC2,CD247,CST7,CCL4,XCL1,HCST,GZMH,TRGC1,IL32,IL2RB,CD160,CD96,MATK,EVL |
| Epithelial cell | cluster2 | Enterocyte | 1.79684736203864e-34 | 1.90142577993506e-36 | NA | 8/33 | 26/494 | FABP1,KRT8,KRT18,PIGR,TSPAN8,EPCAM,AGR2,TFF3 | FABP1,PHGR1,KRT8,KRT18,LGALS4,PIGR,KRT19,TSPAN8,EPCAM,REG1A,ELF3,AGR2,FXYD3,CEACAM5,LGALS3,CKB,OLFM4,S100A6,MT1G,CD24,CLDN4,S100A14,C15orf48,TFF3,CLDN3,GPX2 |
|  | cluster6 | Goblet cell | 0.00166914753009858 | 0.000194292305090839 | NA | 3/81 | 3/631 | UBE2C,MKI67,AGR2 | STMN1,REG3A,AGR2 |
|  | cluster15 | Goblet cell | 1.79654939329027e-73 | 9.50555234545114e-76 | NA | 13/81 | 27/631 | SPINK4,TFF3,MUC2,REG4,CLCA1,AGR2,ITLN1,ZG16,WFDC2,TFF1,EPCAM,MUC5B,REP15 | SPINK4,TFF3,MUC2,REG4,CLCA1,AGR2,FCGBP,ITLN1,ZG16,WFDC2,SPINK1,ST6GALNAC1,TFF1,FAM3D,EPCAM,FXYD3,STARD10,KLK1,AGR3,ELF3,CEACAM5,MUC5B,HEPACAM2,CLDN3,CLDN4,REP15,SMIM22 |
| Endothelial cell | cluster4 | Vein endothelial cell | 7.5583423187142e-23 | 1.19973687598638e-24 | NA | 5/25 | 13/110 | ACKR1,VWF,PECAM1,PLVAP,SELE | ACKR1,SPARCL1,VWF,CLU,IGFBP7,EGFL7,CLDN5,SOCS3,TSPAN7,RAMP3,NPDC1,TGFBR2,HSPG2 |
|  | cluster10 | Endothelial cell | 1.9126435236796e-45 | 1.01198070035958e-47 | NA | 22/466 | 28/319 | COL4A1,SLC9A3R2,PLVAP,RAMP2,FABP4,A2M,GNG11,TM4SF1,CAV1,SPARC,IGFBP7,PODXL,CLDN5,INSR,SPARCL1,PECAM1,CDH5,EGFL7,ENG,CD93,FLT1,ADGRL4 | IGFBP3,COL4A1,HSPG2,COL15A1,SLC9A3R2,PLVAP,RAMP2,FABP4,A2M,COL4A2,GNG11,TM4SF1,CAV1,SPARC,IGFBP7,SOX18,PODXL,CLDN5,SPARCL1,PECAM1,CRIP2,ID1,CDH5,EGFL7,ENG,CD93,FLT1,ADGRL4 |
| Myeloid cell | cluster7 | Monocyte | 3.42997705684495e-85 | 1.81480267557932e-87 | NA | 23/218 | 27/242 | S100A9,S100A8,IL1B,CXCL8,LYZ,FCN1,FCER1G,TIMP1,TYROBP,SOD2,CXCL2,VCAN,CD14,AIF1,NAMPT,FTH1,CTSS,CTSB,FTL,SAT1,S100A12,EREG,SRGN | S100A9,S100A8,IL1B,CXCL8,LYZ,CCL3,FCN1,FCER1G,TIMP1,TYROBP,SOD2,PLAUR,CXCL2,VCAN,CD14,G0S2,AIF1,NAMPT,FTH1,CTSS,CTSB,BCL2A1,FTL,SAT1,S100A12,EREG,SRGN |
|  | cluster8 | Macrophage | 1.39515956791849e-101 | 7.38179665565336e-104 | NA | 28/281 | 29/653 | C1QA,C1QB,APOE,C1QC,LYZ,HLA-DQA1,CD14,HLA-DRA,MS4A6A,APOC1,TYROBP,AIF1,CST3,HLA-DPA1,HLA-DRB1,FCER1G,HLA-DQB1,FTL,CTSB,SELENOP,HLA-DPB1,CD74,PSAP,FCGR3A,HLA-DQA2,CD68,TMEM176B,TYMP | C1QA,C1QB,APOE,C1QC,LYZ,HLA-DQA1,CD14,HLA-DRA,MS4A6A,APOC1,TYROBP,AIF1,CST3,HLA-DPA1,HLA-DRB1,FCER1G,HLA-DQB1,FTL,CTSB,SELENOP,HLA-DPB1,CD74,PSAP,FCGR3A,HLA-DQA2,CD68,TMEM176B,CTSZ,TYMP |
| Fibroblast | cluster12 | Cancer-associated fibroblast | 1.59879092334258e-49 | 2.53776337038505e-51 | NA | 10/25 | 10/213 | DCN,COL3A1,COL1A1,COL1A2,CFD,COL6A3,COL6A2,COL6A1,FN1,SPARC | COL3A1,COL1A1,COL1A2,LUM,MGP,C1S,C1R,RARRES2,FN1,TIMP1 |
|  | cluster13 | Myofibroblast cell | 2.56455594268092e-59 | 2.71381581236076e-61 | NA | 13/72 | 24/251 | ACTA2,TAGLN,MYL9,MYH11,RGS5,IGFBP5,PLN,MCAM,IGFBP7,MYLK,MFGE8,PDGFRB,ACTG2 | ACTA2,TAGLN,C11orf96,MYL9,TPM2,MYH11,CALD1,RGS5,CARMN,IGFBP5,DSTN,TPM1,PLN,SOD3,CSRP2,IGFBP7,MYLK,SELENOM,CSRP1,MFGE8,PPP1R14A,FLNA,FRZB,ACTG2 |

**Table*. S2.*** ShRNA&siRNA Sequences Used in This Study.

| Species | Name | Target Sequence |
| --- | --- | --- |
| Human | sh-S100A14_homo#1 | CAACCTGGGCAGCTGCAATGA |
|  | sh-S100A14_homo#2 | CCTCATCAAGAACTTTCACCA |
|  | sh-S100A14_homo#3 | CCCATCTCATGCCGAGCAACT |
|  | sh-S100A14_homo#4 | GATGCTCAGGAATTCAGTGAT |
| Mouse | sh-S100A14_mus#1 | GAAGCTTCTGGGAGTTGATTG |
|  | sh-S100A14_mus#2 | CTCATCAAGAACTTCCATAAA |
|  | sh-S100A14_mus#3 | GCAACTGTAATGACTCGAAAC |
|  | sh-S100A14_mus#4 | GACTTCTACTTGGAACTTGTT |
| Human & Mouse | siUPF1#1 | CCTGAGCTGCAGAAGCTGCA |
|  | siUPF1#2 | CTGTAATACCAGCAAGAAGT |
|  | siUPF1#3 | CCAAGACTGACTCTGACAT |

**Table*. S3.*** Antibodies Used in This Study.

| **Target** | **Vendor** | **Catalogue Number** | **Dilution ratio** | **Application** |
| --- | --- | --- | --- | --- |
| S100A14 | Proteintech | 10489-1-AP | 1:1000(WB); 1:2000(mIHC) | WB, mIHC |
| UPF1 | Abclonal | A5071 | 1:1000(WB); 1:100(IF); 1:100(IP) | WB, IF, IP |
| GAPDH | HUABIO | SA30-01 | 1:10000 | WB |
| N-cadherin | Proteintech | 22018-1-AP | 1:5000 | WB |
| E-cadherin | Cell Signaling Technology | 3195T | 1:1000 | WB |
| Vimentin | Cell Signaling Technology | 5741T | 1:1000 | WB |
| RelB | Cell Signaling Technology | 4922T | 1:1000 | WB |
| NFKB2 | Cell Signaling Technology | 4882T | 1:1000 | WB |
| CD274 | Proteintech | 17952-1-AP | 1:1000 | WB |
| Flag | Immunoway | YM3808 | 1:10000 (WB); 1:1000 (IF); 1:100 (IP) | WB, IF, IP |
| p-CHUK | Immunoway | YP0140 | 1:1000 | WB |
| CHUK | Immunoway | YM8329 | 1:1000 | WB |
| MAP3K14 | Immunoway | YN1594 | 1:1000 | WB |
| Ubiquitin | Immunoway | YM3636 | 1:1000 | WB |
| PE Anti-Mouse Foxp3 | Elabscience | E-AB-F1238D | 5 ul/test | FACS |
| PerCP/Cyanine5.5 Anti-Mouse CD4 | Elabscience | E-AB-F1353J | 5 ul/test | FACS |
| APC Anti-Mouse CD8a | Elabscience | E-AB-F1104E | 5 ul/test | FACS |
| PE anti-human/mouse Granzyme B | BioLegend | 372207 | 5 ul/test | FACS |
| Foxp3 | AiFang Biologcal | AFRP0014 | 1:200 | IF |
| CD4 | AiFang Biologcal | AF20210 | 1:200 | IF |
| GZMB | AiFang Biologcal | AFRM0352 | 1:200 | IF |
| CD8 | AiFang Biologcal | AFRM0004 | 1:2000(mIHC); 1:200(IF) | mIHC, IF |
| CD3 | Abcam | ab16669 | 1:500 | mIHC |
| PAN-CK | AiFang Biologcal | AF20164 | 1:4000 | mIHC |
| Goat Anti Rabbit IgG(H+L) (HRP) | Immunoway | RS0002 | 1:500 | WB |
| Goat Anti Mouse IgG(H+L) (HRP) | Immunoway | RS0001 | 1:500 | WB |
| Goat Anti Mouse IgG(H+L) (AbFluor 647) | Immunoway | RS3808 | 1:500 | IF |
| Goat Anti Rabbit IgG(H+L) (AbFluor 568) | Immunoway | RS3511 | 1:500 | IF |

**Table*. S4.*** proteins_IPMAS.

|  | **Protein.Group** | **Protein.ID** | **Accession** | **X.10lgP** | **Coverage....** | **Coverage.....IP** | **Area.IP** | **X.Peptides** | **X.Unique** | **X.Spec.IP** | **PTM** | **Avg..Mass** | **Description** |
| --- | --- | --- | --- | --- | --- | --- | --- | --- | --- | --- | --- | --- | --- |
| P52732 | 1 | 3 | P52732 | 379.15 | 49 | 49 | 524350000 | 69 | 69 | 102 | Carbamidomethylation; Deamidation (NQ); Oxidation (M); Dehydration; Pyro-glu from E; 3 more | 119159 | Kinesin-like protein KIF11 OS=Homo sapiens OX=9606 GN=KIF11 PE=1 SV=2 |
| Q13885 | 11 | 12 | Q13885 | 301.95 | 49 | 49 | 122580 | 23 | 3 | 51 | Carbamidomethylation; Deamidation (NQ); Oxidation (M); Acetylation (N-term); Carbamidomethylation (DHKE X@N-term); 2 more | 49907 | Tubulin beta-2A chain OS=Homo sapiens OX=9606 GN=TUBB2A PE=1 SV=1 |
| P60709 | 9 | 16 | P60709 | 295.98 | 65 | 65 | 1335300 | 26 | 1 | 53 | Carbamidomethylation; Deamidation (NQ); Oxidation (M); Acetylation (Protein N-term); Acetylation (N-term); 6 more | 41737 | Actin cytoplasmic 1 OS=Homo sapiens OX=9606 GN=ACTB PE=1 SV=1 |
| Q71U36 | 17 | 20 | Q71U36 | 271.05 | 42 | 42 | 2034900 | 17 | 1 | 34 | Carbamidomethylation; Deamidation (NQ); Oxidation (M); Pyro-glu from Q; Dethiomethyl | 50136 | Tubulin alpha-1A chain OS=Homo sapiens OX=9606 GN=TUBA1A PE=1 SV=1 |
| Q13509 | 15 | 21 | Q13509 | 261.61 | 38 | 38 | 1988100 | 18 | 2 | 37 | Carbamidomethylation; Deamidation (NQ); Oxidation (M); Dehydration; Dethiomethyl | 50433 | Tubulin beta-3 chain OS=Homo sapiens OX=9606 GN=TUBB3 PE=1 SV=2 |
| P68366 | 20 | 31 | P68366 | 241.98 | 33 | 33 | 0 | 15 | 1 | 30 | Carbamidomethylation; Deamidation (NQ); Oxidation (M); Pyro-glu from Q; Dethiomethyl | 49924 | Tubulin alpha-4A chain OS=Homo sapiens OX=9606 GN=TUBA4A PE=1 SV=1 |
| P67809 | 76 | 45 | P67809 | 217.58 | 60 | 60 | 15063000 | 9 | 9 | 9 | Deamidation (NQ); Oxidation (M); Acetylation (Protein N-term) | 35924 | Y-box-binding protein 1 OS=Homo sapiens OX=9606 GN=YBX1 PE=1 SV=3 |
| P68032 | 18 | 53 | P68032 | 214.02 | 32 | 32 | 3215500 | 16 | 1 | 32 | Deamidation (NQ); Oxidation (M); Acetylation (N-term); Sodium adduct; Pyridylacetyl; Tryptophan oxidation to kynurenin | 42019 | Actin alpha cardiac muscle 1 OS=Homo sapiens OX=9606 GN=ACTC1 PE=1 SV=1 |
| Q08211 | 34 | 26 | Q08211 | 202.56 | 13 | 13 | 20552000 | 14 | 14 | 17 | Carbamidomethylation | 140958 | ATP-dependent RNA helicase A OS=Homo sapiens OX=9606 GN=DHX9 PE=1 SV=4 |
| Q7L2E3 | 47 | 41 | Q7L2E3 | 201.82 | 13 | 13 | 16510000 | 14 | 14 | 14 | Carbamidomethylation; Oxidation (M) | 133938 | ATP-dependent RNA helicase DHX30 OS=Homo sapiens OX=9606 GN=DHX30 PE=1 SV=1 |
| O00159 | 48 | 43 | O00159 | 189.57 | 14 | 14 | 6648800 | 11 | 10 | 14 | Carbamidomethylation; Deamidation (NQ) | 121682 | Unconventional myosin-Ic OS=Homo sapiens OX=9606 GN=MYO1C PE=1 SV=4 |
| P52597 | 62 | 64 | P52597 | 188.51 | 29 | 29 | 31088000 | 9 | 7 | 10 | Oxidation (M); Acetylation (Protein N-term) | 45672 | Heterogeneous nuclear ribonucleoprotein F OS=Homo sapiens OX=9606 GN=HNRNPF PE=1 SV=3 |
| Q9UM54 | 36 | 38 | Q9UM54 | 187.44 | 15 | 15 | 22549000 | 15 | 15 | 16 | Carbamidomethylation | 149691 | Unconventional myosin-VI OS=Homo sapiens OX=9606 GN=MYO6 PE=1 SV=4 |
| P08865 | 86 | 88 | P08865 | 180.25 | 33 | 33 | 11030000 | 6 | 6 | 8 | Carbamidomethylation | 32854 | Small ribosomal subunit protein uS2 OS=Homo sapiens OX=9606 GN=RPSA PE=1 SV=4 |
| P05787 | 37 | 67 | P05787 | 178.12 | 22 | 22 | 11513000 | 14 | 6 | 16 |  | 53704 | Keratin type II cytoskeletal 8 OS=Homo sapiens OX=9606 GN=KRT8 PE=1 SV=7 |
| Q92900 | 39 | 56 | Q92900 | 171.84 | 17 | 17 | 13111000 | 14 | 14 | 16 | Carbamidomethylation | 124345 | Regulator of nonsense transcripts 1 OS=Homo sapiens OX=9606 GN=UPF1 PE=1 SV=2 |
| P08727 | 45 | 65 | P08727 | 171.79 | 27 | 27 | 6093600 | 13 | 5 | 15 |  | 44106 | Keratin type I cytoskeletal 19 OS=Homo sapiens OX=9606 GN=KRT19 PE=1 SV=4 |
| Q9HCY8 | 139 | 118 | Q9HCY8 | 164.26 | 67 | 67 | 17319000 | 5 | 5 | 5 | Carbamidomethylation | 11662 | Protein S100-A14 OS=Homo sapiens OX=9606 GN=S100A14 PE=1 SV=1 |
| O43795 | 43 | 84 | O43795 | 163.47 | 14 | 14 | 9284100 | 13 | 12 | 15 | Carbamidomethylation | 131985 | Unconventional myosin-Ib OS=Homo sapiens OX=9606 GN=MYO1B PE=1 SV=3 |
| P07195 | 105 | 89 | P07195 | 162.1 | 21 | 21 | 9693900 | 6 | 5 | 6 | Carbamidomethylation | 36639 | L-lactate dehydrogenase B chain OS=Homo sapiens OX=9606 GN=LDHB PE=1 SV=2 |
| P09211 | 161 | 145 | P09211 | 161.59 | 27 | 27 | 14104000 | 4 | 4 | 4 | Carbamidomethylation | 23356 | Glutathione S-transferase P OS=Homo sapiens OX=9606 GN=GSTP1 PE=1 SV=2 |
| P60842 | 82 | 95 | P60842 | 159.32 | 19 | 19 | 7599300 | 7 | 6 | 8 | Carbamidomethylation | 46154 | Eukaryotic initiation factor 4A-I OS=Homo sapiens OX=9606 GN=EIF4A1 PE=1 SV=1 |
| P05783 | 57 | 75 | P05783 | 157.49 | 23 | 23 | 9043400 | 9 | 6 | 12 |  | 48058 | Keratin type I cytoskeletal 18 OS=Homo sapiens OX=9606 GN=KRT18 PE=1 SV=2 |
| Q15293 | 128 | 111 | Q15293 | 151.41 | 21 | 21 | 9579200 | 5 | 5 | 5 |  | 38890 | Reticulocalbin-1 OS=Homo sapiens OX=9606 GN=RCN1 PE=1 SV=1 |
| P07355 | 95 | 116 | P07355 | 150.49 | 20 | 20 | 10605000 | 6 | 6 | 7 | Carbamidomethylation; Acetylation (Protein N-term) | 38604 | Annexin A2 OS=Homo sapiens OX=9606 GN=ANXA2 PE=1 SV=2 |
| P06576 | 96 | 106 | P06576 | 149.81 | 18 | 18 | 31234000 | 6 | 6 | 7 | Oxidation (M); Glycidamide adduct | 56560 | ATP synthase subunit beta mitochondrial OS=Homo sapiens OX=9606 GN=ATP5F1B PE=1 SV=3 |
| Q00839 | 107 | 113 | Q00839 | 148.98 | 10 | 10 | 5748100 | 5 | 5 | 6 | Deamidation (NQ) | 90585 | Heterogeneous nuclear ribonucleoprotein U OS=Homo sapiens OX=9606 GN=HNRNPU PE=1 SV=6 |
| P19338 | 103 | 107 | P19338 | 147.45 | 12 | 12 | 7964900 | 6 | 6 | 6 |  | 76615 | Nucleolin OS=Homo sapiens OX=9606 GN=NCL PE=1 SV=3 |
| Q12905 | 133 | 129 | Q12905 | 147.39 | 19 | 19 | 6376300 | 5 | 5 | 5 |  | 43062 | Interleukin enhancer-binding factor 2 OS=Homo sapiens OX=9606 GN=ILF2 PE=1 SV=2 |
| O43852 | 78 | 128 | O43852 | 145.6 | 24 | 24 | 44222000 | 6 | 6 | 9 | Oxidation (M); Dehydration | 37107 | Calumenin OS=Homo sapiens OX=9606 GN=CALU PE=1 SV=2 |
| Q15393 | 117 | 119 | Q15393 | 141.97 | 5 | 5 | 5834100 | 5 | 5 | 5 |  | 135577 | Splicing factor 3B subunit 3 OS=Homo sapiens OX=9606 GN=SF3B3 PE=1 SV=4 |
| P52565 | 115 | 181 | P52565 | 141.6 | 40 | 40 | 5766300 | 5 | 5 | 6 | Deamidation (NQ); Acetylation (Protein N-term) | 23207 | Rho GDP-dissociation inhibitor 1 OS=Homo sapiens OX=9606 GN=ARHGDIA PE=1 SV=3 |
| Q02543 | 162 | 192 | Q02543 | 140.61 | 20 | 20 | 7127100 | 4 | 4 | 4 | Carbamidomethylation | 20762 | Large ribosomal subunit protein eL20 OS=Homo sapiens OX=9606 GN=RPL18A PE=1 SV=2 |
| P62826 | 112 | 124 | P62826 | 140.56 | 27 | 27 | 11932000 | 6 | 6 | 6 | Acetylation (Protein N-term) | 24423 | GTP-binding nuclear protein Ran OS=Homo sapiens OX=9606 GN=RAN PE=1 SV=3 |
| P04792 | 165 | 162 | P04792 | 139.74 | 26 | 26 | 8239500 | 4 | 4 | 4 |  | 22783 | Heat shock protein beta-1 OS=Homo sapiens OX=9606 GN=HSPB1 PE=1 SV=2 |
| P13010 | 90 | 93 | P13010 | 139.48 | 9 | 9 | 9325700 | 7 | 7 | 7 | Carbamidomethylation | 82705 | X-ray repair cross-complementing protein 5 OS=Homo sapiens OX=9606 GN=XRCC5 PE=1 SV=3 |
| P35222 | 122 | 115 | P35222 | 135.67 | 7 | 7 | 2256900 | 5 | 5 | 5 | Carbamidomethylation | 85497 | Catenin beta-1 OS=Homo sapiens OX=9606 GN=CTNNB1 PE=1 SV=1 |
| P61247 | 106 | 126 | P61247 | 135.14 | 23 | 23 | 5570500 | 6 | 6 | 6 | Carbamidomethylation | 29945 | Small ribosomal subunit protein eS1 OS=Homo sapiens OX=9606 GN=RPS3A PE=1 SV=2 |
| P12268 | 73 | 148 | P12268 | 132.22 | 12 | 12 | 9146100 | 7 | 7 | 9 |  | 55805 | Inosine-5'-monophosphate dehydrogenase 2 OS=Homo sapiens OX=9606 GN=IMPDH2 PE=1 SV=2 |
| P11021 | 74 | 185 | P11021 | 132.08 | 6 | 6 | 3328400 | 4 | 2 | 9 | Chlorination of tyrosine residues | 72333 | Endoplasmic reticulum chaperone BiP OS=Homo sapiens OX=9606 GN=HSPA5 PE=1 SV=2 |
| P51149 | 151 | 160 | P51149 | 131.98 | 23 | 23 | 2010300 | 4 | 4 | 4 |  | 23490 | Ras-related protein Rab-7a OS=Homo sapiens OX=9606 GN=RAB7A PE=1 SV=1 |
| P62241 | 136 | 163 | P62241 | 131.7 | 25 | 25 | 3362000 | 4 | 4 | 5 | Carbamidomethylation; Pyro-glu from Q | 24205 | Small ribosomal subunit protein eS8 OS=Homo sapiens OX=9606 GN=RPS8 PE=1 SV=2 |
| P32119 | 98 | 152 | P32119 | 131.07 | 34 | 34 | 13164000 | 6 | 4 | 7 | Pyro-glu from Q | 21892 | Peroxiredoxin-2 OS=Homo sapiens OX=9606 GN=PRDX2 PE=1 SV=5 |
| P46781 | 85 | 149 | P46781 | 130.67 | 32 | 32 | 16969000 | 8 | 8 | 8 |  | 22591 | Small ribosomal subunit protein uS4 OS=Homo sapiens OX=9606 GN=RPS9 PE=1 SV=3 |
| P63244 | 130 | 171 | P63244 | 128.93 | 15 | 15 | 2587300 | 5 | 5 | 5 |  | 35077 | Small ribosomal subunit protein RACK1 OS=Homo sapiens OX=9606 GN=RACK1 PE=1 SV=3 |
| P22392 | 97 | 168 | P22392 | 127.4 | 32 | 32 | 15679000 | 4 | 4 | 7 | Carbamidomethylation; Oxidation (M) | 17298 | Nucleoside diphosphate kinase B OS=Homo sapiens OX=9606 GN=NME2 PE=1 SV=1 |
| Q5T6F2 | 168 | 178 | Q5T6F2 | 127.27 | 5 | 5 | 2016000 | 4 | 4 | 4 |  | 117115 | Ubiquitin-associated protein 2 OS=Homo sapiens OX=9606 GN=UBAP2 PE=1 SV=1 |
| P40926 | 113 | 108 | P40926 | 126.78 | 23 | 23 | 7629500 | 6 | 6 | 6 |  | 35503 | Malate dehydrogenase mitochondrial OS=Homo sapiens OX=9606 GN=MDH2 PE=1 SV=3 |
| Q07020 | 213 | 197 | Q07020 | 126.64 | 20 | 20 | 10718000 | 3 | 3 | 3 | Carbamidomethylation | 21634 | Large ribosomal subunit protein eL18 OS=Homo sapiens OX=9606 GN=RPL18 PE=1 SV=2 |
| P46782 | 171 | 219 | P46782 | 125.3 | 20 | 20 | 9242300 | 4 | 4 | 4 | Carbamidomethylation; Acetylation (Protein N-term) | 22876 | Small ribosomal subunit protein uS7 OS=Homo sapiens OX=9606 GN=RPS5 PE=1 SV=4 |
| P53597 | 89 | 487 | P53597 | 125.1 | 7 | 7 | 92751000 | 4 | 4 | 8 | Deamidation (NQ); Dihydroxy; Glycidamide adduct | 36250 | Succinate--CoA ligase [ADP/GDP-forming] subunit alpha mitochondrial OS=Homo sapiens OX=9606 GN=SUCLG1 PE=1 SV=4 |
| Q15365 | 140 | 136 | Q15365 | 124.44 | 19 | 19 | 2793600 | 5 | 3 | 5 | Carbamidomethylation; Oxidation (M) | 37498 | Poly(rC)-binding protein 1 OS=Homo sapiens OX=9606 GN=PCBP1 PE=1 SV=2 |
| P62906 | 100 | 193 | P62906 | 124.36 | 30 | 30 | 13620000 | 6 | 6 | 7 | Carbamidomethylation; Oxidation (M) | 24831 | Large ribosomal subunit protein uL1 OS=Homo sapiens OX=9606 GN=RPL10A PE=1 SV=2 |
| Q15366 | 141 | 133 | Q15366 | 123.61 | 19 | 19 | 5640300 | 5 | 3 | 5 | Carbamidomethylation; Oxidation (M) | 38580 | Poly(rC)-binding protein 2 OS=Homo sapiens OX=9606 GN=PCBP2 PE=1 SV=1 |
| P33993 | 129 | 137 | P33993 | 123.49 | 8 | 8 | 2560700 | 5 | 5 | 5 |  | 81308 | DNA replication licensing factor MCM7 OS=Homo sapiens OX=9606 GN=MCM7 PE=1 SV=4 |
| O00299 | 217 | 218 | O00299 | 120.51 | 16 | 16 | 1777100 | 3 | 3 | 3 |  | 26923 | Chloride intracellular channel protein 1 OS=Homo sapiens OX=9606 GN=CLIC1 PE=1 SV=4 |
| O43143 | 149 | 179 | O43143 | 119.85 | 5 | 5 | 4224300 | 4 | 3 | 4 |  | 90933 | ATP-dependent RNA helicase DHX15 OS=Homo sapiens OX=9606 GN=DHX15 PE=1 SV=2 |
| P07737 | 218 | 266 | P07737 | 119.81 | 33 | 33 | 3549100 | 3 | 3 | 3 | Oxidation (M) | 15054 | Profilin-1 OS=Homo sapiens OX=9606 GN=PFN1 PE=1 SV=2 |
| Q9NZB2 | 126 | 213 | Q9NZB2 | 119.31 | 7 | 7 | 2080300 | 5 | 5 | 5 | Carbamidomethylation | 121888 | Constitutive coactivator of PPAR-gamma-like protein 1 OS=Homo sapiens OX=9606 GN=FAM120A PE=1 SV=2 |
| P15880 | 158 | 174 | P15880 | 118.7 | 14 | 14 | 8978800 | 4 | 4 | 4 |  | 31324 | Small ribosomal subunit protein uS5 OS=Homo sapiens OX=9606 GN=RPS2 PE=1 SV=2 |
| P27348 | 159 | 189 | P27348 | 117.97 | 21 | 21 | 1196800 | 4 | 2 | 4 | Oxidation (M) | 27764 | 14-3-3 protein theta OS=Homo sapiens OX=9606 GN=YWHAQ PE=1 SV=1 |
| P30050 | 214 | 263 | P30050 | 117.01 | 24 | 24 | 6814700 | 3 | 3 | 3 | Carbamidomethylation | 17819 | Large ribosomal subunit protein uL11 OS=Homo sapiens OX=9606 GN=RPL12 PE=1 SV=1 |
| O00571 | 178 | 184 | O00571 | 115.86 | 6 | 6 | 2151500 | 3 | 3 | 3 | Carbamidomethylation | 73244 | ATP-dependent RNA helicase DDX3X OS=Homo sapiens OX=9606 GN=DDX3X PE=1 SV=3 |
| Q14103 | 143 | 236 | Q14103 | 114.31 | 9 | 9 | 3826400 | 3 | 2 | 5 | Carbamidomethylation | 38434 | Heterogeneous nuclear ribonucleoprotein D0 OS=Homo sapiens OX=9606 GN=HNRNPD PE=1 SV=1 |
| P62820 | 155 | 250 | P62820 | 111.83 | 21 | 21 | 3560400 | 4 | 3 | 4 | Carbamidomethylation | 22678 | Ras-related protein Rab-1A OS=Homo sapiens OX=9606 GN=RAB1A PE=1 SV=3 |
| P84103 | 163 | 247 | P84103 | 111.41 | 29 | 29 | 4846300 | 3 | 3 | 4 | Deamidation (NQ) | 19330 | Serine/arginine-rich splicing factor 3 OS=Homo sapiens OX=9606 GN=SRSF3 PE=1 SV=1 |
| P62917 | 160 | 202 | P62917 | 111.4 | 15 | 15 | 5272700 | 3 | 3 | 4 |  | 28025 | Large ribosomal subunit protein uL2 OS=Homo sapiens OX=9606 GN=RPL8 PE=1 SV=2 |
| Q15907 | 138 | 187 | Q15907 | 110.67 | 21 | 21 | 3920700 | 5 | 5 | 5 |  | 24488 | Ras-related protein Rab-11B OS=Homo sapiens OX=9606 GN=RAB11B PE=1 SV=4 |
| P62857 | 216 | 359 | P62857 | 110.09 | 38 | 38 | 1831500 | 3 | 3 | 3 | Carbamidomethylation | 7841 | Small ribosomal subunit protein eS28 OS=Homo sapiens OX=9606 GN=RPS28 PE=1 SV=1 |
| P62258 | 142 | 151 | P62258 | 109.91 | 21 | 21 | 4836300 | 5 | 3 | 5 | Oxidation (M); Acetylation (Protein N-term) | 29174 | 14-3-3 protein epsilon OS=Homo sapiens OX=9606 GN=YWHAE PE=1 SV=1 |
| P60866 | 135 | 287 | P60866 | 107.56 | 29 | 29 | 8669600 | 4 | 4 | 5 | Carbamidomethylation | 13373 | Small ribosomal subunit protein uS10 OS=Homo sapiens OX=9606 GN=RPS20 PE=1 SV=1 |
| P61106 | 198 | 357 | P61106 | 106.96 | 12 | 12 | 2101000 | 3 | 2 | 3 |  | 23897 | Ras-related protein Rab-14 OS=Homo sapiens OX=9606 GN=RAB14 PE=1 SV=4 |
| P61981 | 192 | 205 | P61981 | 106.76 | 13 | 13 | 1173500 | 3 | 1 | 3 | Oxidation (M) | 28303 | 14-3-3 protein gamma OS=Homo sapiens OX=9606 GN=YWHAG PE=1 SV=2 |
| O43809 | 189 | 272 | O43809 | 106.03 | 23 | 23 | 2959200 | 3 | 3 | 3 |  | 26227 | Cleavage and polyadenylation specificity factor subunit 5 OS=Homo sapiens OX=9606 GN=NUDT21 PE=1 SV=1 |
| P26373 | 124 | 190 | P26373 | 105.77 | 23 | 23 | 8620600 | 5 | 5 | 5 |  | 24261 | Large ribosomal subunit protein eL13 OS=Homo sapiens OX=9606 GN=RPL13 PE=1 SV=4 |
| P12004 | 237 | 298 | P12004 | 104.94 | 10 | 10 | 1355200 | 2 | 2 | 2 |  | 28769 | Proliferating cell nuclear antigen OS=Homo sapiens OX=9606 GN=PCNA PE=1 SV=1 |
| Q14444 | 156 | 227 | Q14444 | 104.52 | 5 | 5 | 2464700 | 4 | 4 | 4 |  | 78366 | Caprin-1 OS=Homo sapiens OX=9606 GN=CAPRIN1 PE=1 SV=2 |
| Q01813 | 119 | 226 | Q01813 | 104.07 | 5 | 5 | 6582300 | 4 | 4 | 5 | Oxidation (M); Acetylation (Protein N-term) | 85596 | ATP-dependent 6-phosphofructokinase platelet type OS=Homo sapiens OX=9606 GN=PFKP PE=1 SV=2 |
| P50914 | 205 | 256 | P50914 | 103.89 | 16 | 16 | 9119600 | 3 | 2 | 3 |  | 23432 | Large ribosomal subunit protein eL14 OS=Homo sapiens OX=9606 GN=RPL14 PE=1 SV=4 |
| O00267 | 123 | 232 | O00267 | 102.47 | 6 | 6 | 1671800 | 4 | 4 | 5 | Carbamidomethylation; Deamidation (NQ) | 121000 | Transcription elongation factor SPT5 OS=Homo sapiens OX=9606 GN=SUPT5H PE=1 SV=1 |
| P48047 | 127 | 238 | P48047 | 101.71 | 16 | 16 | 3411800 | 3 | 3 | 5 |  | 23277 | ATP synthase subunit O mitochondrial OS=Homo sapiens OX=9606 GN=ATP5PO PE=1 SV=1 |
| P39687 | 87 | 255 | P39687 | 100.81 | 15 | 15 | 4310300 | 4 | 3 | 8 |  | 28585 | Acidic leucine-rich nuclear phosphoprotein 32 family member A OS=Homo sapiens OX=9606 GN=ANP32A PE=1 SV=1 |
| P24539 | 196 | 270 | P24539 | 100.37 | 13 | 13 | 3422000 | 3 | 3 | 3 |  | 28909 | ATP synthase F(0) complex subunit B1 mitochondrial OS=Homo sapiens OX=9606 GN=ATP5PB PE=1 SV=2 |
| P62750 | 206 | 283 | P62750 | 98.61 | 22 | 22 | 8559300 | 3 | 3 | 3 |  | 17695 | Large ribosomal subunit protein uL23 OS=Homo sapiens OX=9606 GN=RPL23A PE=1 SV=1 |
| Q13162 | 137 | 282 | Q13162 | 97.65 | 12 | 12 | 212090 | 4 | 1 | 5 |  | 30540 | Peroxiredoxin-4 OS=Homo sapiens OX=9606 GN=PRDX4 PE=1 SV=1 |
| P50991 | 121 | 198 | P50991 | 96.86 | 9 | 9 | 1632800 | 4 | 4 | 5 | Carbamidomethylation | 57924 | T-complex protein 1 subunit delta OS=Homo sapiens OX=9606 GN=CCT4 PE=1 SV=4 |
| Q8WWM7 | 185 | 262 | Q8WWM7 | 96.67 | 4 | 4 | 3109200 | 3 | 3 | 3 |  | 113374 | Ataxin-2-like protein OS=Homo sapiens OX=9606 GN=ATXN2L PE=1 SV=2 |
| P25787 | 201 | 331 | P25787 | 94.72 | 14 | 14 | 271280 | 3 | 3 | 3 |  | 25899 | Proteasome subunit alpha type-2 OS=Homo sapiens OX=9606 GN=PSMA2 PE=1 SV=2 |
| P50990 | 190 | 206 | P50990 | 94.69 | 7 | 7 | 2150700 | 3 | 3 | 3 |  | 59621 | T-complex protein 1 subunit theta OS=Homo sapiens OX=9606 GN=CCT8 PE=1 SV=4 |
| P62249 | 152 | 210 | P62249 | 94.66 | 21 | 21 | 4074600 | 4 | 4 | 4 |  | 16445 | Small ribosomal subunit protein uS9 OS=Homo sapiens OX=9606 GN=RPS16 PE=1 SV=2 |
| Q00610 | 144 | 230 | Q00610 | 93.69 | 4 | 4 | 1823600 | 4 | 4 | 4 |  | 191613 | Clathrin heavy chain 1 OS=Homo sapiens OX=9606 GN=CLTC PE=1 SV=5 |
| Q12906 | 194 | 237 | Q12906 | 93.68 | 4 | 4 | 1922000 | 3 | 3 | 3 |  | 95339 | Interleukin enhancer-binding factor 3 OS=Homo sapiens OX=9606 GN=ILF3 PE=1 SV=3 |
| Q13435 | 184 | 209 | Q13435 | 93.04 | 5 | 5 | 3886300 | 3 | 3 | 3 |  | 100228 | Splicing factor 3B subunit 2 OS=Homo sapiens OX=9606 GN=SF3B2 PE=1 SV=2 |
| Q01844 | 260 | 348 | Q01844 | 93.04 | 4 | 4 | 4210800 | 2 | 2 | 2 |  | 68478 | RNA-binding protein EWS OS=Homo sapiens OX=9606 GN=EWSR1 PE=1 SV=1 |
| P49368 | 176 | 261 | P49368 | 92.98 | 6 | 6 | 2550200 | 3 | 3 | 3 |  | 60534 | T-complex protein 1 subunit gamma OS=Homo sapiens OX=9606 GN=CCT3 PE=1 SV=4 |
| Q99497 | 242 | 300 | Q99497 | 91.46 | 22 | 22 | 871140 | 2 | 2 | 2 | Carbamidomethylation | 19891 | Parkinson disease protein 7 OS=Homo sapiens OX=9606 GN=PARK7 PE=1 SV=2 |
| Q8N163 | 219 | 315 | Q8N163 | 90.94 | 3 | 3 | 1919200 | 2 | 2 | 2 |  | 102902 | Cell cycle and apoptosis regulator protein 2 OS=Homo sapiens OX=9606 GN=CCAR2 PE=1 SV=2 |
| P28074 | 230 | 291 | P28074 | 90.92 | 10 | 10 | 2334900 | 2 | 2 | 2 |  | 28480 | Proteasome subunit beta type-5 OS=Homo sapiens OX=9606 GN=PSMB5 PE=1 SV=3 |
| P51991 | 303 | 403 | P51991 | 89.55 | 6 | 6 | 641950 | 1 | 1 | 1 |  | 39595 | Heterogeneous nuclear ribonucleoprotein A3 OS=Homo sapiens OX=9606 GN=HNRNPA3 PE=1 SV=2 |
| P33992 | 146 | 249 | P33992 | 87.87 | 5 | 5 | 1364100 | 3 | 3 | 4 | Deamidation (NQ) | 82286 | DNA replication licensing factor MCM5 OS=Homo sapiens OX=9606 GN=MCM5 PE=1 SV=5 |
| P07741 | 215 | 275 | P07741 | 86.55 | 19 | 19 | 959240 | 3 | 3 | 3 | Acetylation (Protein N-term) | 19608 | Adenine phosphoribosyltransferase OS=Homo sapiens OX=9606 GN=APRT PE=1 SV=2 |
| P18621 | 173 | 241 | P18621 | 86.12 | 16 | 16 | 4718800 | 3 | 3 | 4 |  | 21397 | Large ribosomal subunit protein uL22 OS=Homo sapiens OX=9606 GN=RPL17 PE=1 SV=3 |
| P31947 | 199 | 212 | P31947 | 85.71 | 12 | 12 | 1328200 | 3 | 1 | 3 | Oxidation (M) | 27774 | 14-3-3 protein sigma OS=Homo sapiens OX=9606 GN=SFN PE=1 SV=1 |
| P17987 | 183 | 196 | P17987 | 83.92 | 6 | 6 | 1669300 | 3 | 3 | 3 |  | 60344 | T-complex protein 1 subunit alpha OS=Homo sapiens OX=9606 GN=TCP1 PE=1 SV=1 |
| P30041 | 200 | 274 | P30041 | 83.76 | 12 | 12 | 2675600 | 3 | 3 | 3 |  | 25035 | Peroxiredoxin-6 OS=Homo sapiens OX=9606 GN=PRDX6 PE=1 SV=3 |
| P08574 | 239 | 365 | P08574 | 82.66 | 9 | 9 | 1498600 | 2 | 2 | 2 |  | 35422 | Cytochrome c1 heme protein mitochondrial OS=Homo sapiens OX=9606 GN=CYC1 PE=1 SV=3 |
| Q04837 | 263 | 361 | Q04837 | 81.75 | 16 | 16 | 986650 | 2 | 2 | 2 |  | 17260 | Single-stranded DNA-binding protein mitochondrial OS=Homo sapiens OX=9606 GN=SSBP1 PE=1 SV=1 |
| Q9Y617 | 235 | 329 | Q9Y617 | 80.53 | 6 | 6 | 619600 | 2 | 2 | 2 |  | 40423 | Phosphoserine aminotransferase OS=Homo sapiens OX=9606 GN=PSAT1 PE=1 SV=2 |
| P31689 | 234 | 326 | P31689 | 79.31 | 7 | 7 | 2197200 | 2 | 2 | 2 |  | 44868 | DnaJ homolog subfamily A member 1 OS=Homo sapiens OX=9606 GN=DNAJA1 PE=1 SV=2 |
| Q6PKG0 | 182 | 273 | Q6PKG0 | 79.07 | 3 | 3 | 1410300 | 3 | 3 | 3 |  | 123510 | La-related protein 1 OS=Homo sapiens OX=9606 GN=LARP1 PE=1 SV=2 |
| P35232 | 195 | 254 | P35232 | 78.57 | 11 | 11 | 1866800 | 3 | 3 | 3 |  | 29804 | Prohibitin 1 OS=Homo sapiens OX=9606 GN=PHB1 PE=1 SV=1 |
| Q92688 | 180 | 293 | Q92688 | 78.33 | 8 | 8 | 3075700 | 2 | 1 | 3 | Carbamidomethylation | 28788 | Acidic leucine-rich nuclear phosphoprotein 32 family member B OS=Homo sapiens OX=9606 GN=ANP32B PE=1 SV=1 |
| Q00266 | 255 | 486 | Q00266 | 78.08 | 6 | 6 | 3326900 | 2 | 1 | 2 | Deoxy | 43648 | S-adenosylmethionine synthase isoform type-1 OS=Homo sapiens OX=9606 GN=MAT1A PE=1 SV=2 |
| Q6P5R6 | 203 | 347 | Q6P5R6 | 77.85 | 20 | 20 | 1257300 | 2 | 2 | 3 | 2-amino-3-oxo-butanoic_acid | 14607 | Ribosomal protein eL22-like OS=Homo sapiens OX=9606 GN=RPL22L1 PE=1 SV=2 |
| P18124 | 223 | 295 | P18124 | 77.41 | 10 | 10 | 2457700 | 2 | 2 | 2 |  | 29226 | Large ribosomal subunit protein uL30 OS=Homo sapiens OX=9606 GN=RPL7 PE=1 SV=1 |
| Q16531 | 197 | 342 | Q16531 | 76.69 | 3 | 3 | 558800 | 2 | 2 | 3 | Carbamidomethylation | 126968 | DNA damage-binding protein 1 OS=Homo sapiens OX=9606 GN=DDB1 PE=1 SV=1 |
| Q9HCE1 | 188 | 265 | Q9HCE1 | 76.3 | 4 | 4 | 1005800 | 3 | 3 | 3 |  | 113671 | Helicase MOV-10 OS=Homo sapiens OX=9606 GN=MOV10 PE=1 SV=2 |
| Q8WX93 | 177 | 288 | Q8WX93 | 75.76 | 2 | 2 | 1887100 | 2 | 2 | 3 | Deamidation (NQ) | 150564 | Palladin OS=Homo sapiens OX=9606 GN=PALLD PE=1 SV=3 |
| P32969 | 240 | 398 | P32969 | 75.73 | 6 | 6 | 7901300 | 2 | 2 | 2 |  | 21863 | Large ribosomal subunit protein uL6 OS=Homo sapiens OX=9606 GN=RPL9P9 PE=1 SV=1 |
| Q99729 | 175 | 411 | Q99729 | 75.28 | 5 | 5 | 298430 | 2 | 1 | 4 | Carbamidomethylation | 36225 | Heterogeneous nuclear ribonucleoprotein A/B OS=Homo sapiens OX=9606 GN=HNRNPAB PE=1 SV=2 |
| P49720 | 341 | 476 | P49720 | 73.93 | 8 | 8 | 437120 | 1 | 1 | 1 |  | 22949 | Proteasome subunit beta type-3 OS=Homo sapiens OX=9606 GN=PSMB3 PE=1 SV=2 |
| P53621 | 220 | 330 | P53621 | 73.13 | 2 | 2 | 664460 | 2 | 2 | 2 |  | 138345 | Coatomer subunit alpha OS=Homo sapiens OX=9606 GN=COPA PE=1 SV=2 |
| P83731 | 244 | 409 | P83731 | 72.74 | 8 | 8 | 3093400 | 2 | 2 | 2 |  | 17779 | Large ribosomal subunit protein eL24 OS=Homo sapiens OX=9606 GN=RPL24 PE=1 SV=1 |
| P60900 | 245 | 294 | P60900 | 72.64 | 9 | 9 | 1419100 | 2 | 2 | 2 |  | 27399 | Proteasome subunit alpha type-6 OS=Homo sapiens OX=9606 GN=PSMA6 PE=1 SV=1 |
| P05388 | 289 | 304 | P05388 | 72.12 | 4 | 4 | 616790 | 1 | 1 | 1 |  | 34274 | Large ribosomal subunit protein uL10 OS=Homo sapiens OX=9606 GN=RPLP0 PE=1 SV=1 |
| P62888 | 336 | 400 | P62888 | 72.1 | 14 | 14 | 753860 | 1 | 1 | 1 | Carbamidomethylation | 12784 | Large ribosomal subunit protein eL30 OS=Homo sapiens OX=9606 GN=RPL30 PE=1 SV=2 |
| P61604 | 247 | 364 | P61604 | 70.61 | 19 | 19 | 1999600 | 2 | 2 | 2 |  | 10932 | 10 kDa heat shock protein mitochondrial OS=Homo sapiens OX=9606 GN=HSPE1 PE=1 SV=2 |
| P00403 | 261 | 565 | P00403 | 70.41 | 7 | 7 | 993840 | 2 | 2 | 2 |  | 25565 | Cytochrome c oxidase subunit 2 OS=Homo sapiens OX=9606 GN=MT-CO2 PE=1 SV=1 |
| P30048 | 210 | 284 | P30048 | 70.3 | 14 | 14 | 1983800 | 3 | 3 | 3 | Deamidation (NQ) | 27693 | Thioredoxin-dependent peroxide reductase mitochondrial OS=Homo sapiens OX=9606 GN=PRDX3 PE=1 SV=3 |
| P62277 | 208 | 336 | P62277 | 69.98 | 15 | 15 | 1480100 | 2 | 2 | 3 |  | 17222 | Small ribosomal subunit protein uS15 OS=Homo sapiens OX=9606 GN=RPS13 PE=1 SV=2 |
| P46777 | 164 | 308 | P46777 | 68.54 | 9 | 9 | 1097100 | 2 | 2 | 4 | Carbamidomethylation | 34363 | Large ribosomal subunit protein uL18 OS=Homo sapiens OX=9606 GN=RPL5 PE=1 SV=3 |
| Q99623 | 207 | 285 | Q99623 | 68.48 | 9 | 9 | 913720 | 3 | 3 | 3 |  | 33296 | Prohibitin-2 OS=Homo sapiens OX=9606 GN=PHB2 PE=1 SV=2 |
| Q9ULX3 | 252 | 307 | Q9ULX3 | 68.02 | 7 | 7 | 2833000 | 2 | 2 | 2 |  | 46675 | RNA-binding protein NOB1 OS=Homo sapiens OX=9606 GN=NOB1 PE=1 SV=1 |
| O00303 | 249 | 468 | O00303 | 67.09 | 5 | 5 | 1063700 | 1 | 1 | 2 |  | 37564 | Eukaryotic translation initiation factor 3 subunit F OS=Homo sapiens OX=9606 GN=EIF3F PE=1 SV=1 |
| Q9NR30 | 225 | 366 | Q9NR30 | 65.94 | 3 | 3 | 1054400 | 2 | 2 | 2 |  | 87344 | Nucleolar RNA helicase 2 OS=Homo sapiens OX=9606 GN=DDX21 PE=1 SV=5 |
| Q15417 | 312 | 457 | Q15417 | 65.74 | 5 | 5 | 1091400 | 1 | 1 | 1 | Acetylation (Protein N-term) | 36414 | Calponin-3 OS=Homo sapiens OX=9606 GN=CNN3 PE=1 SV=1 |
| O14818 | 221 | 319 | O14818 | 63.8 | 10 | 10 | 1530600 | 2 | 2 | 2 |  | 27887 | Proteasome subunit alpha type-7 OS=Homo sapiens OX=9606 GN=PSMA7 PE=1 SV=1 |
| P51398 | 256 | 379 | P51398 | 62.98 | 6 | 6 | 368410 | 2 | 2 | 2 |  | 45566 | Small ribosomal subunit protein mS29 OS=Homo sapiens OX=9606 GN=DAP3 PE=1 SV=1 |
| P43243 | 229 | 305 | P43243 | 61.61 | 3 | 3 | 778710 | 2 | 2 | 2 |  | 94623 | Matrin-3 OS=Homo sapiens OX=9606 GN=MATR3 PE=1 SV=2 |
| P09651 | 265 | 377 | P09651 | 60.1 | 8 | 8 | 319450 | 2 | 2 | 2 |  | 38747 | Heterogeneous nuclear ribonucleoprotein A1 OS=Homo sapiens OX=9606 GN=HNRNPA1 PE=1 SV=5 |
| P23284 | 211 | 320 | P23284 | 59.68 | 10 | 10 | 1756200 | 2 | 2 | 3 |  | 23743 | Peptidyl-prolyl cis-trans isomerase B OS=Homo sapiens OX=9606 GN=PPIB PE=1 SV=2 |
| O75607 | 331 | 483 | O75607 | 59.57 | 9 | 9 | 47342 | 1 | 1 | 1 | Acetylation (Protein N-term) | 19344 | Nucleoplasmin-3 OS=Homo sapiens OX=9606 GN=NPM3 PE=1 SV=3 |
| Q14694 | 233 | 297 | Q14694 | 59.24 | 3 | 3 | 349090 | 2 | 2 | 2 |  | 87134 | Ubiquitin carboxyl-terminal hydrolase 10 OS=Homo sapiens OX=9606 GN=USP10 PE=1 SV=2 |
| P62081 | 246 | 343 | P62081 | 57.73 | 8 | 8 | 6611800 | 2 | 2 | 2 |  | 22127 | Small ribosomal subunit protein eS7 OS=Homo sapiens OX=9606 GN=RPS7 PE=1 SV=1 |
| P06396 | 231 | 303 | P06396 | 57.59 | 3 | 3 | 999690 | 2 | 2 | 2 |  | 85697 | Gelsolin OS=Homo sapiens OX=9606 GN=GSN PE=1 SV=1 |
| P35268 | 330 | 469 | P35268 | 57.42 | 10 | 10 | 1573500 | 1 | 1 | 1 |  | 14787 | Large ribosomal subunit protein eL22 OS=Homo sapiens OX=9606 GN=RPL22 PE=1 SV=2 |
| P27635 | 181 | 301 | P27635 | 57.22 | 7 | 7 | 2344900 | 1 | 1 | 3 | Oxidation (M) | 24577 | Large ribosomal subunit protein uL16 OS=Homo sapiens OX=9606 GN=RPL10 PE=1 SV=5 |
| P55884 | 276 | 387 | P55884 | 57.21 | 2 | 2 | 249150 | 1 | 1 | 1 |  | 92482 | Eukaryotic translation initiation factor 3 subunit B OS=Homo sapiens OX=9606 GN=EIF3B PE=1 SV=3 |
| P46776 | 343 | 488 | P46776 | 56.85 | 7 | 7 | 4667300 | 1 | 1 | 1 |  | 16561 | Large ribosomal subunit protein uL15 OS=Homo sapiens OX=9606 GN=RPL27A PE=1 SV=2 |
| P60660 | 338 | 406 | P60660 | 56.49 | 9 | 9 | 483370 | 1 | 1 | 1 |  | 16930 | Myosin light polypeptide 6 OS=Homo sapiens OX=9606 GN=MYL6 PE=1 SV=2 |
| P25786 | 297 | 380 | P25786 | 55.96 | 4 | 4 | 1812400 | 1 | 1 | 1 |  | 29556 | Proteasome subunit alpha type-1 OS=Homo sapiens OX=9606 GN=PSMA1 PE=1 SV=1 |
| P50454 | 226 | 309 | P50454 | 55.86 | 6 | 6 | 871160 | 2 | 2 | 2 |  | 46441 | Serpin H1 OS=Homo sapiens OX=9606 GN=SERPINH1 PE=1 SV=2 |
| O00148 | 264 | 367 | O00148 | 55.76 | 4 | 4 | 855910 | 2 | 2 | 2 |  | 49130 | ATP-dependent RNA helicase DDX39A OS=Homo sapiens OX=9606 GN=DDX39A PE=1 SV=2 |
| Q14764 | 224 | 337 | Q14764 | 54.77 | 3 | 3 | 545190 | 2 | 2 | 2 | Carbamidomethylation | 99327 | Major vault protein OS=Homo sapiens OX=9606 GN=MVP PE=1 SV=4 |
| P61353 | 337 | 405 | P61353 | 54.58 | 7 | 7 | 1869900 | 1 | 1 | 1 |  | 15798 | Large ribosomal subunit protein eL27 OS=Homo sapiens OX=9606 GN=RPL27 PE=1 SV=2 |
| P04632 | 394 | 602 | P04632 | 54.22 | 6 | 6 | 681250 | 1 | 1 | 1 |  | 28316 | Calpain small subunit 1 OS=Homo sapiens OX=9606 GN=CAPNS1 PE=1 SV=1 |
| A6NHT5 | 258 | 607 | A6NHT5 | 53.86 | 4 | 4 | 624850 | 2 | 1 | 2 |  | 37825 | Homeobox protein HMX3 OS=Homo sapiens OX=9606 GN=HMX3 PE=1 SV=1 |
| P62280 | 228 | 299 | P62280 | 53.71 | 18 | 18 | 1221300 | 2 | 2 | 2 | Carbamidomethylation | 18431 | Small ribosomal subunit protein uS17 OS=Homo sapiens OX=9606 GN=RPS11 PE=1 SV=3 |
| P25788 | 280 | 360 | P25788 | 52.57 | 5 | 5 | 639420 | 1 | 1 | 1 |  | 28433 | Proteasome subunit alpha type-3 OS=Homo sapiens OX=9606 GN=PSMA3 PE=1 SV=2 |
| P57678 | 374 | 576 | P57678 | 50.18 | 1 | 1 | 834820 | 1 | 1 | 1 | Deamidation (NQ) | 120037 | Gem-associated protein 4 OS=Homo sapiens OX=9606 GN=GEMIN4 PE=1 SV=2 |
| P11586 | 275 | 376 | P11586 | 48.85 | 1 | 1 | 302540 | 1 | 1 | 1 |  | 101531 | C-1-tetrahydrofolate synthase cytoplasmic OS=Homo sapiens OX=9606 GN=MTHFD1 PE=1 SV=4 |
| P62244 | 327 | 395 | P62244 | 48.16 | 8 | 8 | 1622600 | 1 | 1 | 1 |  | 14840 | Small ribosomal subunit protein uS8 OS=Homo sapiens OX=9606 GN=RPS15A PE=1 SV=2 |
| O75477 | 375 | 578 | O75477 | 47.81 | 3 | 3 | 119010 | 1 | 1 | 1 |  | 39171 | Erlin-1 OS=Homo sapiens OX=9606 GN=ERLIN1 PE=1 SV=2 |
| O43324 | 376 | 581 | O43324 | 47.09 | 6 | 6 | 223760 | 1 | 1 | 1 | Acetylation (Protein N-term) | 19811 | Eukaryotic translation elongation factor 1 epsilon-1 OS=Homo sapiens OX=9606 GN=EEF1E1 PE=1 SV=1 |
| O15372 | 362 | 579 | O15372 | 47.06 | 5 | 5 | 132360 | 1 | 1 | 1 |  | 39930 | Eukaryotic translation initiation factor 3 subunit H OS=Homo sapiens OX=9606 GN=EIF3H PE=1 SV=1 |
| O75533 | 273 | 314 | O75533 | 46.85 | 1 | 1 | 390070 | 1 | 1 | 1 |  | 145830 | Splicing factor 3B subunit 1 OS=Homo sapiens OX=9606 GN=SF3B1 PE=1 SV=3 |
| Q9UKK3 | 333 | 496 | Q9UKK3 | 46.84 | 1 | 1 | 45807 | 1 | 1 | 1 |  | 192593 | Protein mono-ADP-ribosyltransferase PARP4 OS=Homo sapiens OX=9606 GN=PARP4 PE=1 SV=3 |
| O43399 | 380 | 609 | O43399 | 45.9 | 7 | 7 | 219160 | 1 | 1 | 1 |  | 22238 | Tumor protein D54 OS=Homo sapiens OX=9606 GN=TPD52L2 PE=1 SV=2 |
| O00231 | 322 | 484 | O00231 | 45.19 | 3 | 3 | 54111 | 1 | 1 | 1 |  | 47464 | 26S proteasome non-ATPase regulatory subunit 11 OS=Homo sapiens OX=9606 GN=PSMD11 PE=1 SV=3 |
| P07237 | 329 | 456 | P07237 | 44.87 | 3 | 3 | 169420 | 1 | 1 | 1 |  | 57116 | Protein disulfide-isomerase OS=Homo sapiens OX=9606 GN=P4HB PE=1 SV=3 |
| P62318 | 357 | 528 | P62318 | 44.39 | 8 | 8 | 943130 | 1 | 1 | 1 |  | 13916 | Small nuclear ribonucleoprotein Sm D3 OS=Homo sapiens OX=9606 GN=SNRPD3 PE=1 SV=1 |
| P19404 | 377 | 583 | P19404 | 44.37 | 5 | 5 | 230270 | 1 | 1 | 1 |  | 27392 | NADH dehydrogenase [ubiquinone] flavoprotein 2 mitochondrial OS=Homo sapiens OX=9606 GN=NDUFV2 PE=1 SV=2 |
| Q9Y3D9 | 344 | 497 | Q9Y3D9 | 44.28 | 5 | 5 | 358230 | 1 | 1 | 1 |  | 21771 | Small ribosomal subunit protein mS23 OS=Homo sapiens OX=9606 GN=MRPS23 PE=1 SV=2 |
| Q96IU4 | 346 | 510 | Q96IU4 | 44.18 | 5 | 5 | 208510 | 1 | 1 | 1 |  | 22346 | Putative protein-lysine deacylase ABHD14B OS=Homo sapiens OX=9606 GN=ABHD14B PE=1 SV=1 |
| Q13126 | 328 | 454 | Q13126 | 44.14 | 6 | 6 | 946180 | 1 | 1 | 1 |  | 31236 | S-methyl-5'-thioadenosine phosphorylase OS=Homo sapiens OX=9606 GN=MTAP PE=1 SV=2 |
| Q02878 | 290 | 306 | Q02878 | 44.03 | 3 | 3 | 891130 | 1 | 1 | 1 |  | 32728 | Large ribosomal subunit protein eL6 OS=Homo sapiens OX=9606 GN=RPL6 PE=1 SV=3 |
| Q2TAY7 | 321 | 416 | Q2TAY7 | 43.52 | 2 | 2 | 284540 | 1 | 1 | 1 |  | 57544 | WD40 repeat-containing protein SMU1 OS=Homo sapiens OX=9606 GN=SMU1 PE=1 SV=2 |
| P57088 | 323 | 502 | P57088 | 43.21 | 5 | 5 | 507200 | 1 | 1 | 1 |  | 27978 | Transmembrane protein 33 OS=Homo sapiens OX=9606 GN=TMEM33 PE=1 SV=2 |
| P25398 | 378 | 586 | P25398 | 43.01 | 8 | 8 | 0 | 1 | 1 | 1 |  | 14515 | Small ribosomal subunit protein eS12 OS=Homo sapiens OX=9606 GN=RPS12 PE=1 SV=3 |
| P12814 | 299 | 470 | P12814 | 42.97 | 1 | 1 | 106420 | 1 | 1 | 1 |  | 103058 | Alpha-actinin-1 OS=Homo sapiens OX=9606 GN=ACTN1 PE=1 SV=2 |
| P39023 | 283 | 381 | P39023 | 42.78 | 3 | 3 | 661190 | 1 | 1 | 1 |  | 46109 | Large ribosomal subunit protein uL3 OS=Homo sapiens OX=9606 GN=RPL3 PE=1 SV=2 |
| P12429 | 291 | 382 | P12429 | 42.4 | 5 | 5 | 559570 | 1 | 1 | 1 |  | 36375 | Annexin A3 OS=Homo sapiens OX=9606 GN=ANXA3 PE=1 SV=3 |
| P52292 | 349 | 589 | P52292 | 41.99 | 3 | 3 | 257780 | 1 | 1 | 1 |  | 57862 | Importin subunit alpha-1 OS=Homo sapiens OX=9606 GN=KPNA2 PE=1 SV=1 |
| P62829 | 355 | 504 | P62829 | 41.79 | 6 | 6 | 898680 | 1 | 1 | 1 |  | 14865 | Large ribosomal subunit protein uL14 OS=Homo sapiens OX=9606 GN=RPL23 PE=1 SV=1 |
| P62314 | 381 | 616 | P62314 | 41.43 | 11 | 11 | 801420 | 1 | 1 | 1 |  | 13282 | Small nuclear ribonucleoprotein Sm D1 OS=Homo sapiens OX=9606 GN=SNRPD1 PE=1 SV=1 |
| P46940 | 279 | 474 | P46940 | 41.34 | 1 | 1 | 347600 | 1 | 1 | 1 |  | 189251 | Ras GTPase-activating-like protein IQGAP1 OS=Homo sapiens OX=9606 GN=IQGAP1 PE=1 SV=1 |
| O00483 | 395 | 621 | O00483 | 41.31 | 12 | 12 | 87937 | 1 | 1 | 1 |  | 9370 | Cytochrome c oxidase subunit NDUFA4 OS=Homo sapiens OX=9606 GN=NDUFA4 PE=1 SV=1 |
| Q12965 | 332 | 493 | Q12965 | 41.21 | 1 | 1 | 442670 | 1 | 1 | 1 |  | 127062 | Unconventional myosin-Ie OS=Homo sapiens OX=9606 GN=MYO1E PE=1 SV=2 |
| P35221 | 286 | 481 | P35221 | 41.11 | 1 | 1 | 119990 | 1 | 1 | 1 |  | 100071 | Catenin alpha-1 OS=Homo sapiens OX=9606 GN=CTNNA1 PE=1 SV=1 |
| P56537 | 367 | 596 | P56537 | 40.3 | 7 | 7 | 642710 | 1 | 1 | 1 |  | 26599 | Eukaryotic translation initiation factor 6 OS=Homo sapiens OX=9606 GN=EIF6 PE=1 SV=1 |
| P09874 | 285 | 479 | P09874 | 40.28 | 1 | 1 | 0 | 1 | 1 | 1 |  | 113084 | Poly [ADP-ribose] polymerase 1 OS=Homo sapiens OX=9606 GN=PARP1 PE=1 SV=4 |
| P28070 | 361 | 567 | P28070 | 40.24 | 4 | 4 | 438540 | 1 | 1 | 1 |  | 29204 | Proteasome subunit beta type-4 OS=Homo sapiens OX=9606 GN=PSMB4 PE=1 SV=4 |
| P67870 | 356 | 507 | P67870 | 40.15 | 5 | 5 | 243790 | 1 | 1 | 1 | Carbamidomethylation | 24942 | Casein kinase II subunit beta OS=Homo sapiens OX=9606 GN=CSNK2B PE=1 SV=1 |
| Q14257 | 366 | 592 | Q14257 | 39.77 | 4 | 4 | 7208300 | 1 | 1 | 1 |  | 36876 | Reticulocalbin-2 OS=Homo sapiens OX=9606 GN=RCN2 PE=1 SV=1 |
| P39656 | 314 | 499 | P39656 | 39.65 | 2 | 2 | 516130 | 1 | 1 | 1 |  | 50801 | Dolichyl-diphosphooligosaccharide--protein glycosyltransferase 48 kDa subunit OS=Homo sapiens OX=9606 GN=DDOST PE=1 SV=4 |
| Q9UKK9 | 379 | 593 | Q9UKK9 | 39.55 | 7 | 7 | 0 | 1 | 1 | 1 |  | 24328 | ADP-sugar pyrophosphatase OS=Homo sapiens OX=9606 GN=NUDT5 PE=1 SV=1 |
| P40925 | 345 | 500 | P40925 | 39.08 | 4 | 4 | 709370 | 1 | 1 | 1 |  | 36426 | Malate dehydrogenase cytoplasmic OS=Homo sapiens OX=9606 GN=MDH1 PE=1 SV=4 |
| P05165 | 238 | 363 | P05165 | 39.06 | 1 | 1 | 6877200 | 1 | 1 | 2 | Oxidation (M) | 80059 | Propionyl-CoA carboxylase alpha chain mitochondrial OS=Homo sapiens OX=9606 GN=PCCA PE=1 SV=4 |
| Q9Y295 | 320 | 388 | Q9Y295 | 38.67 | 3 | 3 | 411510 | 1 | 1 | 1 |  | 40542 | Developmentally-regulated GTP-binding protein 1 OS=Homo sapiens OX=9606 GN=DRG1 PE=1 SV=1 |
| P05386 | 396 | 634 | P05386 | 38.61 | 14 | 14 | 0 | 1 | 1 | 1 |  | 11514 | Large ribosomal subunit protein P1 OS=Homo sapiens OX=9606 GN=RPLP1 PE=1 SV=1 |
| P82933 | 304 | 410 | P82933 | 38.59 | 4 | 4 | 229780 | 1 | 1 | 1 |  | 45835 | Small ribosomal subunit protein uS9m OS=Homo sapiens OX=9606 GN=MRPS9 PE=1 SV=2 |
| Q96DV4 | 358 | 534 | Q96DV4 | 38.32 | 2 | 2 | 122240 | 1 | 1 | 1 |  | 44597 | Large ribosomal subunit protein mL38 OS=Homo sapiens OX=9606 GN=MRPL38 PE=1 SV=2 |
| O75340 | 368 | 605 | O75340 | 38.14 | 6 | 6 | 138440 | 1 | 1 | 1 |  | 21868 | Programmed cell death protein 6 OS=Homo sapiens OX=9606 GN=PDCD6 PE=1 SV=1 |
| Q02790 | 259 | 613 | Q02790 | 38.04 | 2 | 2 | 143220 | 1 | 1 | 2 | Carbamidomethylation | 51805 | Peptidyl-prolyl cis-trans isomerase FKBP4 OS=Homo sapiens OX=9606 GN=FKBP4 PE=1 SV=3 |
| Q92616 | 272 | 509 | Q92616 | 37.53 | 0 | 0 | 2108800 | 1 | 1 | 1 |  | 292708 | Stalled ribosome sensor GCN1 OS=Homo sapiens OX=9606 GN=GCN1 PE=1 SV=7 |
| Q9BUJ2 | 313 | 482 | Q9BUJ2 | 37.41 | 1 | 1 | 455120 | 1 | 1 | 1 |  | 95739 | Heterogeneous nuclear ribonucleoprotein U-like protein 1 OS=Homo sapiens OX=9606 GN=HNRNPUL1 PE=1 SV=2 |
| P08758 | 292 | 424 | P08758 | 37.25 | 3 | 3 | 861750 | 1 | 1 | 1 |  | 35937 | Annexin A5 OS=Homo sapiens OX=9606 GN=ANXA5 PE=1 SV=2 |
| Q92552 | 293 | 425 | Q92552 | 37.22 | 3 | 3 | 258480 | 1 | 1 | 1 |  | 47611 | Small ribosomal subunit protein mS27 OS=Homo sapiens OX=9606 GN=MRPS27 PE=1 SV=3 |
| P23258 | 339 | 429 | P23258 | 37.14 | 3 | 3 | 524210 | 1 | 1 | 1 |  | 51170 | Tubulin gamma-1 chain OS=Homo sapiens OX=9606 GN=TUBG1 PE=1 SV=2 |
| P62805 | 347 | 521 | P62805 | 37.13 | 10 | 10 | 515480 | 1 | 1 | 1 |  | 11367 | Histone H4 OS=Homo sapiens OX=9606 GN=H4C16 PE=1 SV=2 |
| Q15631 | 311 | 431 | Q15631 | 37.08 | 7 | 7 | 460740 | 1 | 1 | 1 |  | 26183 | Translin OS=Homo sapiens OX=9606 GN=TSN PE=1 SV=1 |
| P49736 | 308 | 511 | P49736 | 37.05 | 1 | 1 | 156490 | 1 | 1 | 1 |  | 101896 | DNA replication licensing factor MCM2 OS=Homo sapiens OX=9606 GN=MCM2 PE=1 SV=4 |
| P36551 | 315 | 516 | P36551 | 36.89 | 2 | 2 | 406550 | 1 | 1 | 1 |  | 50152 | Oxygen-dependent coproporphyrinogen-III oxidase mitochondrial OS=Homo sapiens OX=9606 GN=CPOX PE=1 SV=3 |
| Q99613 | 324 | 526 | Q99613 | 36.87 | 1 | 1 | 596350 | 1 | 1 | 1 |  | 105344 | Eukaryotic translation initiation factor 3 subunit C OS=Homo sapiens OX=9606 GN=EIF3C PE=1 SV=1 |
| Q7Z333 | 309 | 517 | Q7Z333 | 36.59 | 0 | 0 | 47214000 | 1 | 1 | 1 |  | 302879 | Probable helicase senataxin OS=Homo sapiens OX=9606 GN=SETX PE=1 SV=4 |
| P04183 | 398 | 661 | P04183 | 36.47 | 9 | 9 | 69100 | 1 | 1 | 1 | Carbamidomethylation | 25469 | Thymidine kinase cytosolic OS=Homo sapiens OX=9606 GN=TK1 PE=1 SV=2 |
| P17931 | 301 | 533 | P17931 | 36.45 | 4 | 4 | 324910 | 1 | 1 | 1 |  | 26152 | Galectin-3 OS=Homo sapiens OX=9606 GN=LGALS3 PE=1 SV=5 |
| P61088 | 382 | 617 | P61088 | 36.31 | 7 | 7 | 224790 | 1 | 1 | 1 |  | 17138 | Ubiquitin-conjugating enzyme E2 N OS=Homo sapiens OX=9606 GN=UBE2N PE=1 SV=1 |

**Table*. S5.*** Primer sequences Used in This Study.

| **Target** | **Species** | **Forward/Reverse primer** | **Sequences** |
| --- | --- | --- | --- |
| ACTB | Human | Forward | CTGGAACGGTGAAGGTGACA |
|  |  | Revers | AAGGGACTTCCTGTAACAATGCA |
|  | Mouse | Forward | CACTGTCGAGTCGCGTCCA |
|  |  | Revers | CATCCATGGCGAACTGGTGG |
| S100A14 | Human | Forward | CCATCTCATGCCGAGCAACTG |
|  |  | Revers | CTTCACACTCTTGGCCGCTT |
|  | Mouse | Forward | AAGGAAACACTGACCCCTGC |
|  |  | Revers | CTAACCCACAGTTGCTCGGC |
| UPF1 | Human | Forward | CTCCGAGTTCGAGTTCACCG |
|  |  | Revers | GCCCAACCTGCGCGT |
|  | Mouse | Forward | GCTCGCAAACACTCACCTTC |
|  |  | Revers | CTCTGGTCCAACTTGTGCGT |
| MAP3K14 | Human | Forward | GGTCCAAACAGTACAGCCAGT |
|  |  | Revers | CTGCATGAGCCAGGGACTTT |
|  | Mouse | Forward | CCCGCTTTGTCTCAAGATTGC |
|  |  | Revers | CTTGCCCACTTTCCTCCGAA |
| CHUK | Human | Forward | GTCTGTACCAGCATCGGGAA |
|  |  | Revers | TGGTTTGTTGAGCAGCTTTCG |
|  | Mouse | Forward | CATTGCAGTATTTGGCCCCAG |
|  |  | Revers | AAAGGCTGTTTGGCTGAGGT |
| NFKB2 | Human | Forward | TAACTACGAGGGACCAGCCA |
|  |  | Revers | CCCAGGTTGTTAAATTGGGCA |
|  | Mouse | Forward | CCCATCCATGACAGCAAGTCT |
|  |  | Revers | TGTGGGAGAGAAGTCCCCAA |
| RELB | Human | Forward | ACAAGGTGCAGAAAGAGGACATA |
|  |  | Revers | CTGTCACGGGCTCGACAATC |
|  | Mouse | Forward | GACAAGGTGCAAAAAGAGGACAT |
|  |  | Revers | CGTAGGGTGGCGTTTTGAAC |
