# Supplementary figures and images for: S100A14 promotes colorectal cancer progression and anti-PD-1 resistance via UPF1-mediated activation of the non-canonical NF-κB signaling
Source: Cell Death Dis. 2026 Jun 25;17(1):597. doi: 10.1038/s41419-026-09032-1 (PMC13303824; doi:10.1038/s41419-026-09032-1)

**Full scans of uncropped blots presented in Figures of the paper**

**
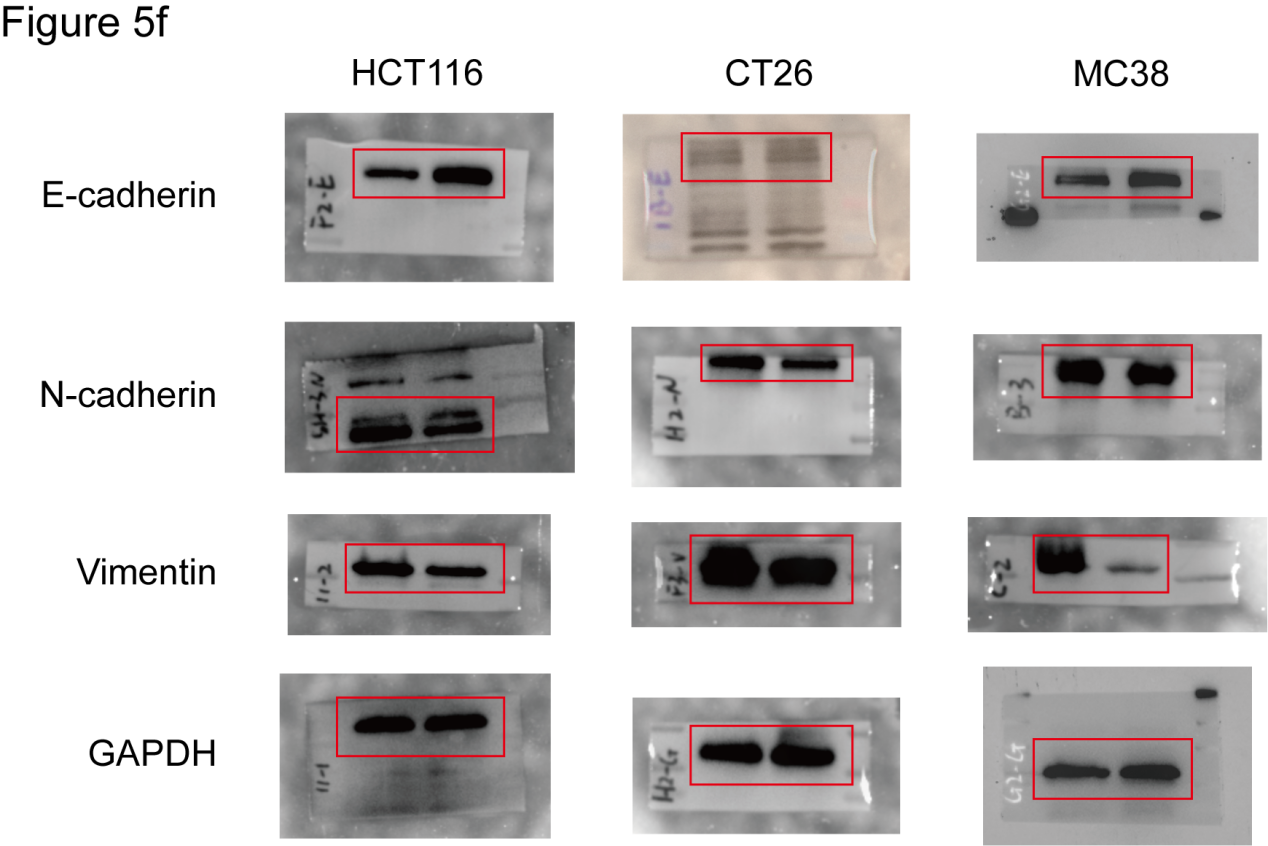

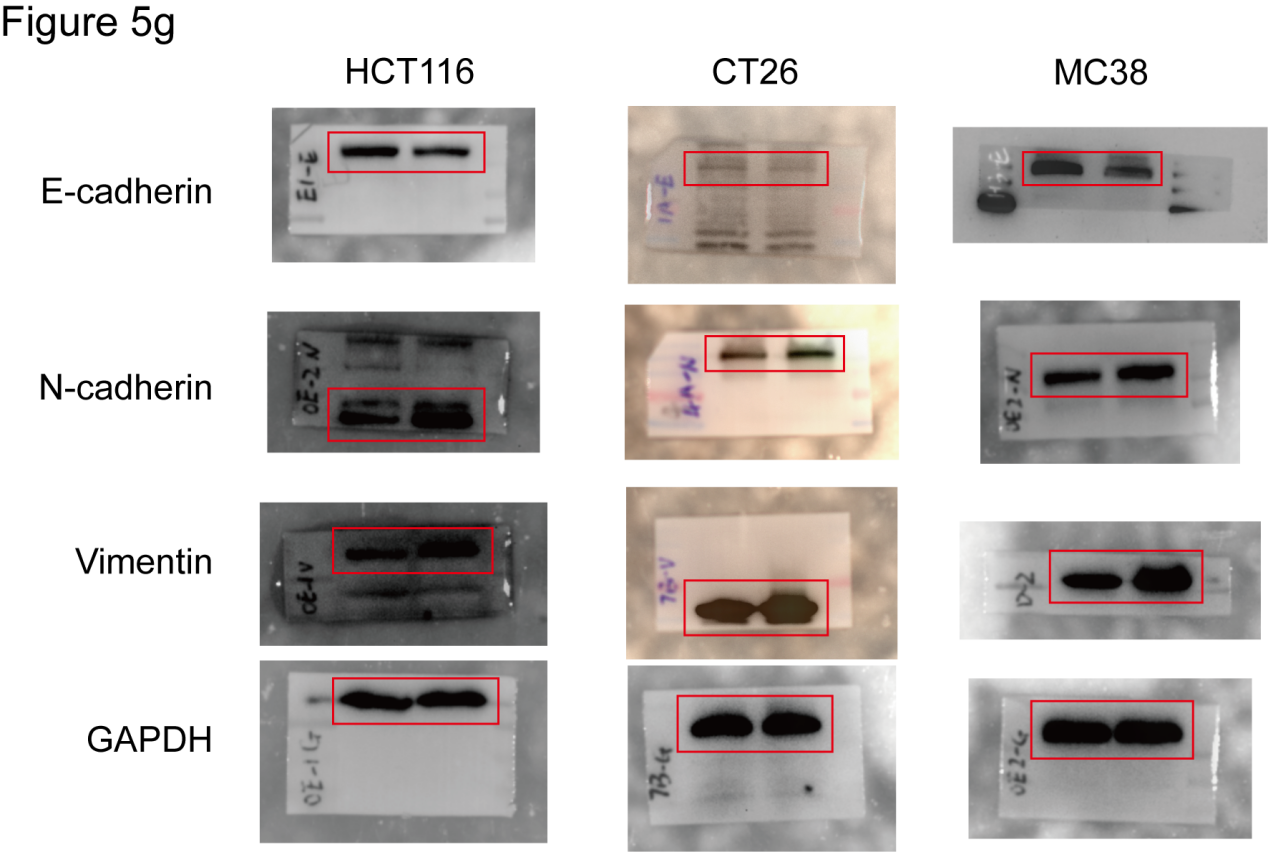

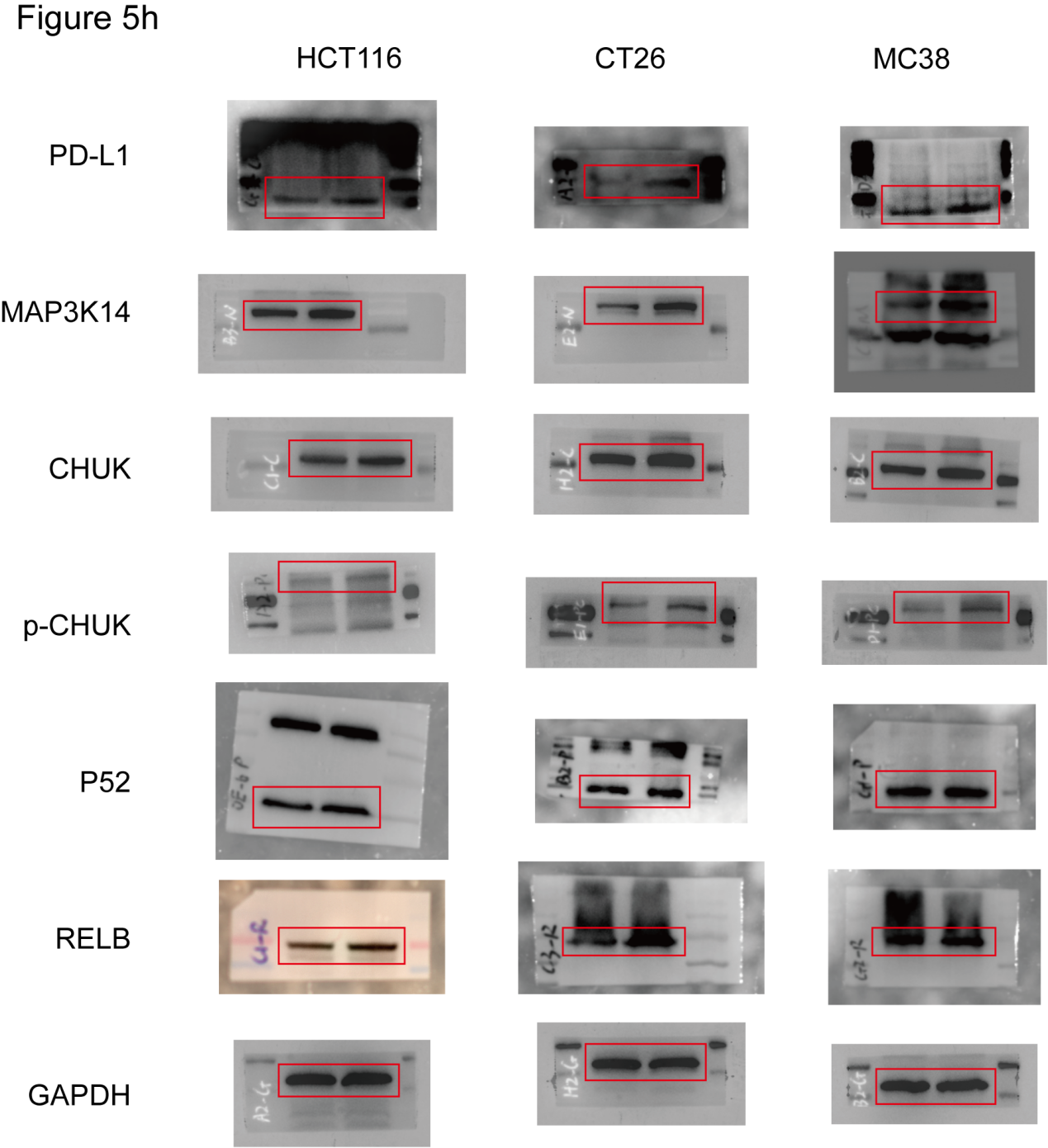

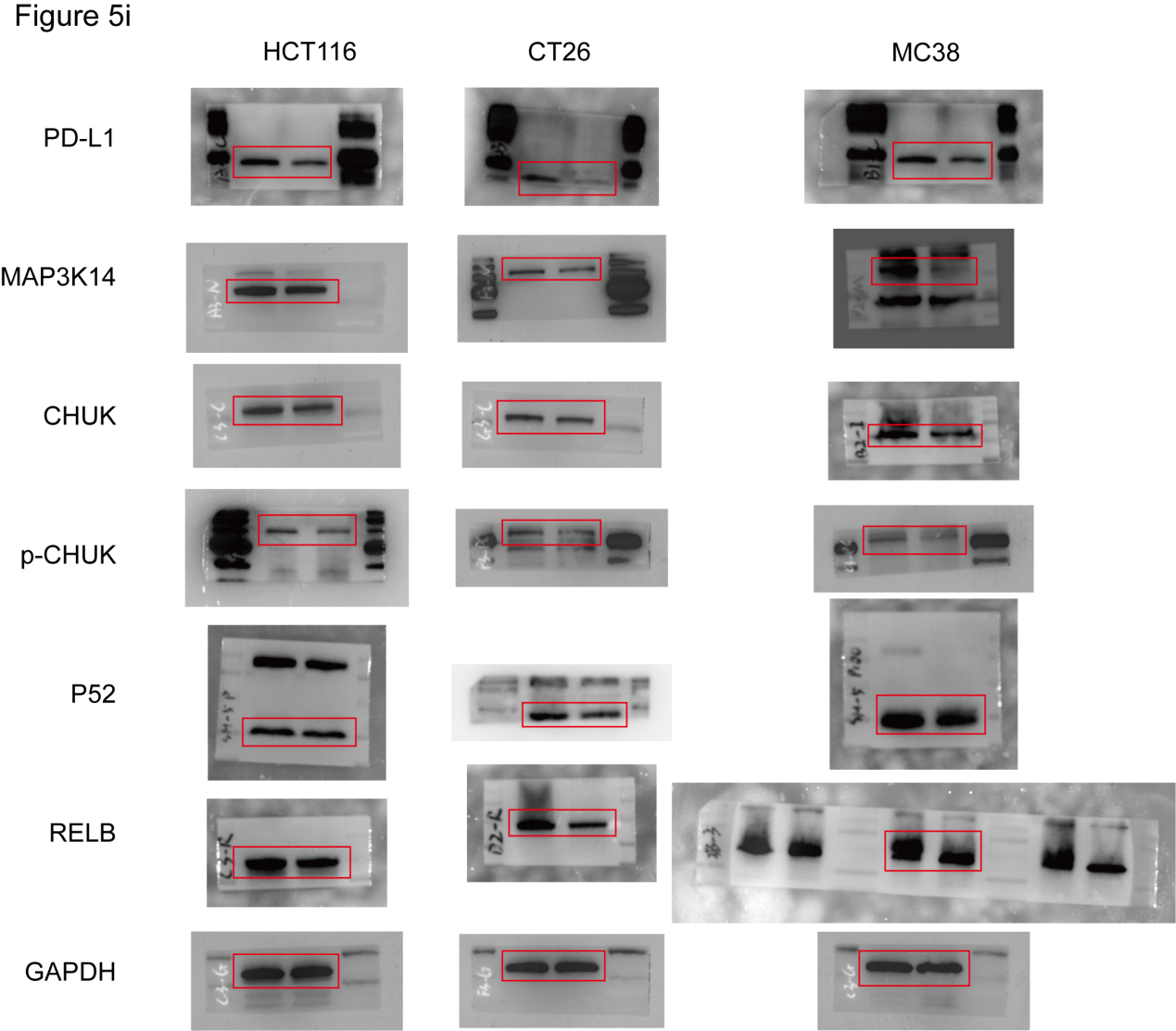

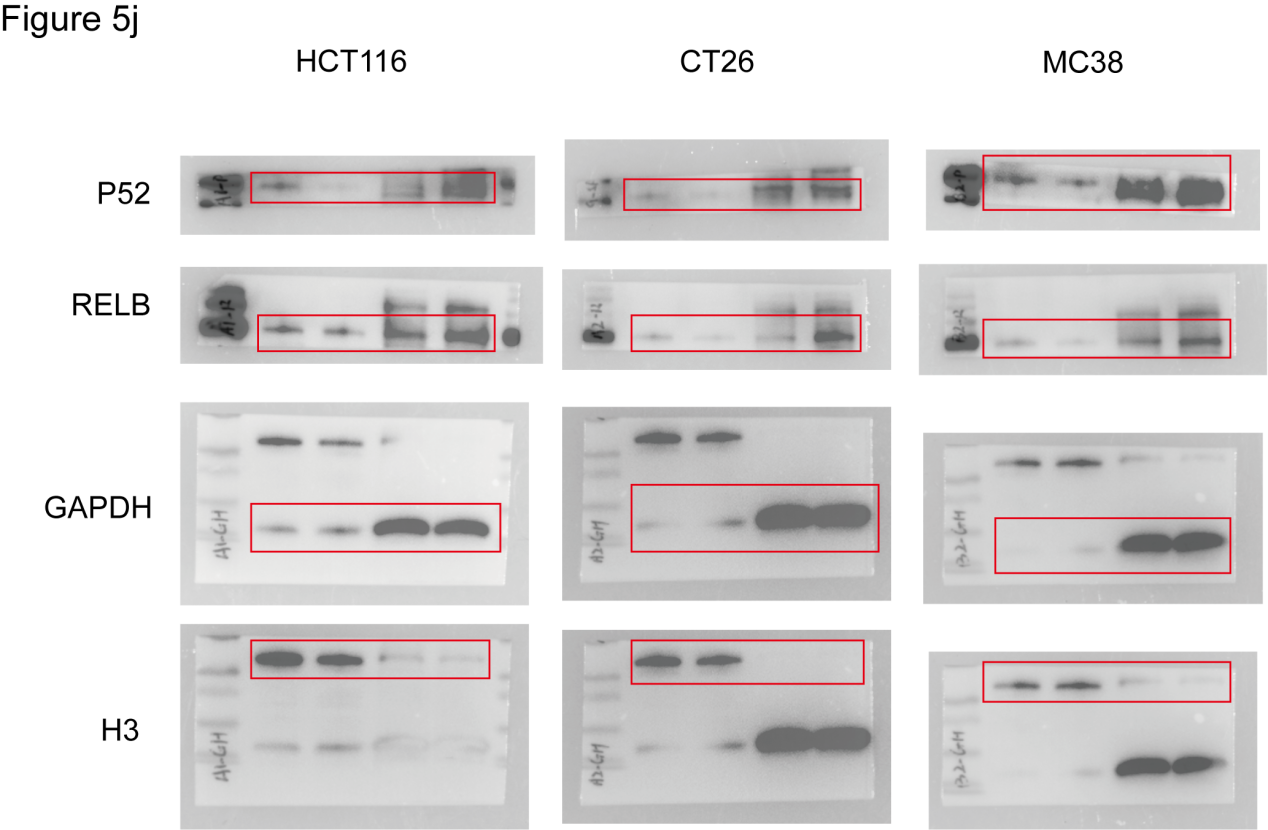

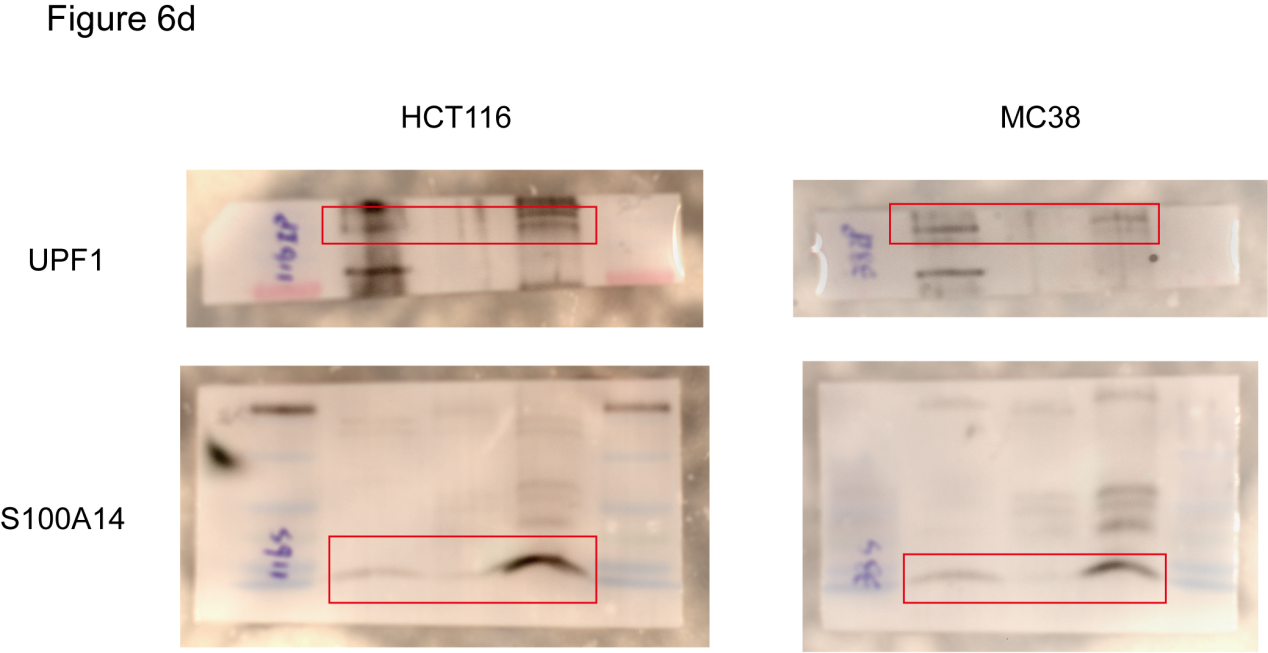

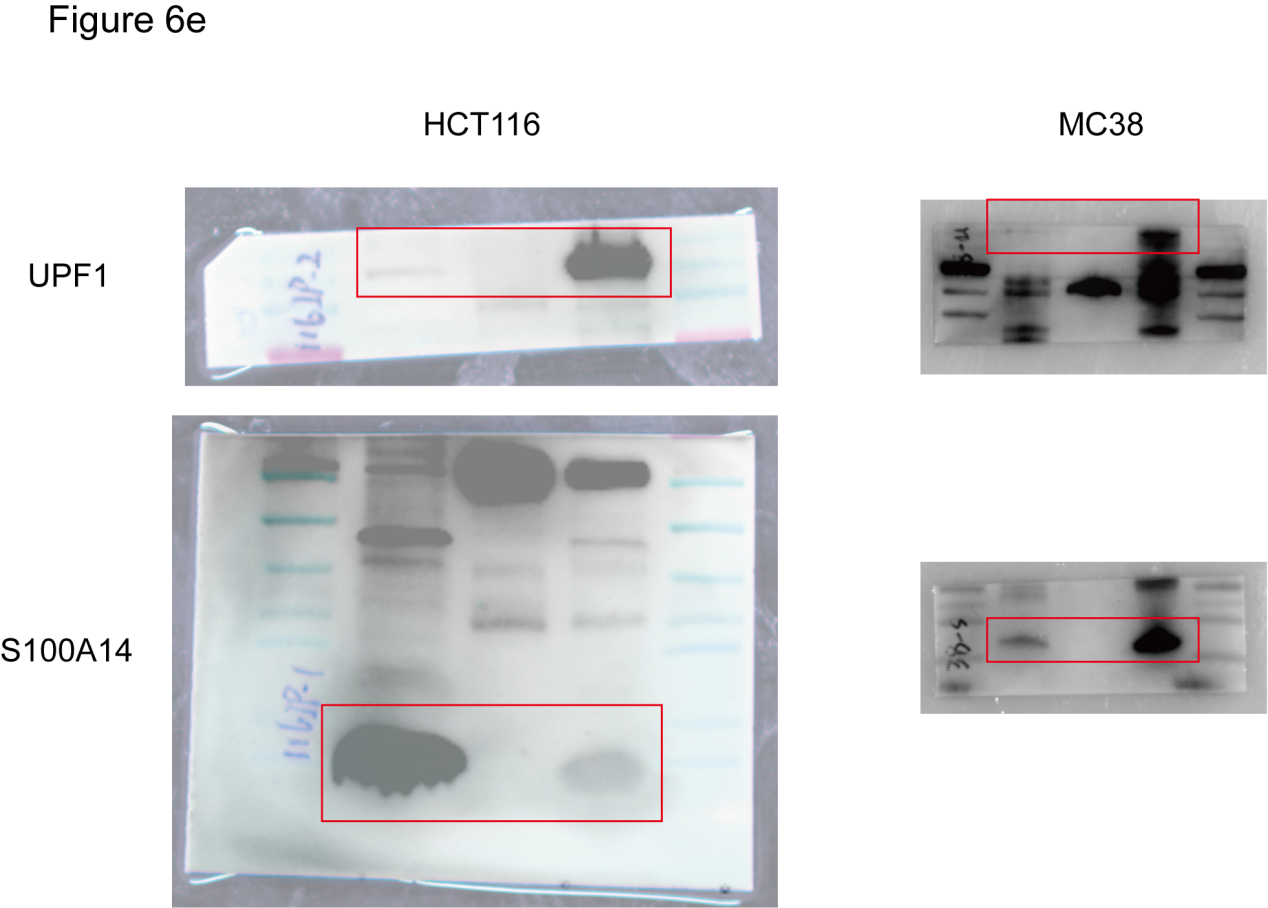

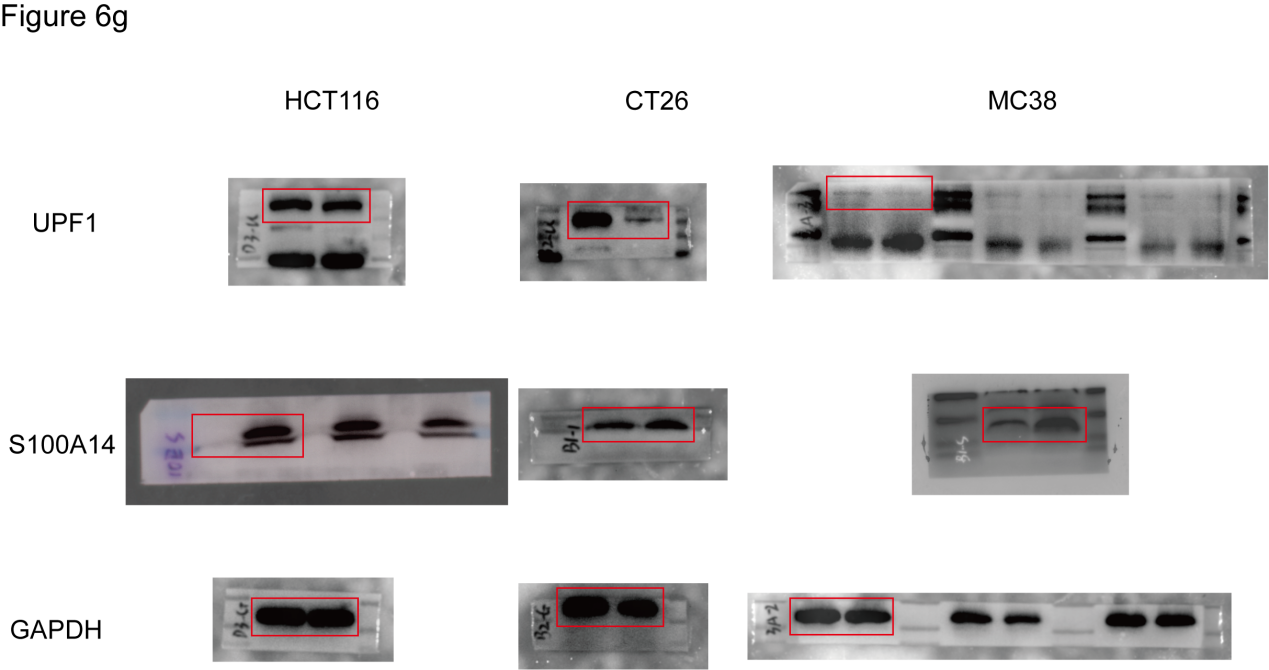

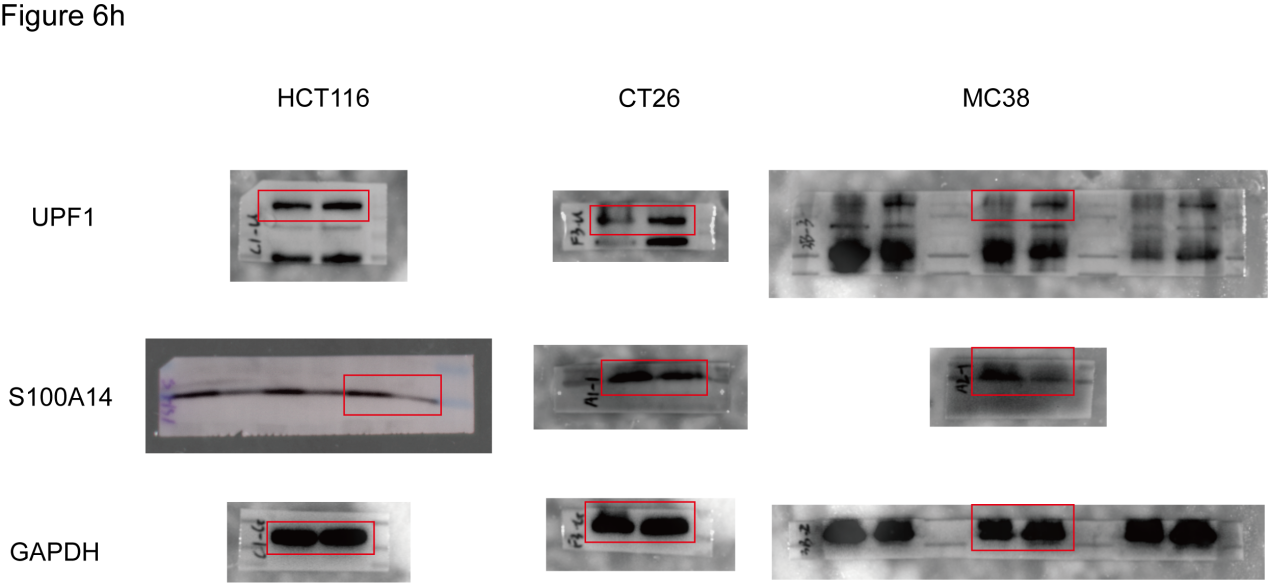

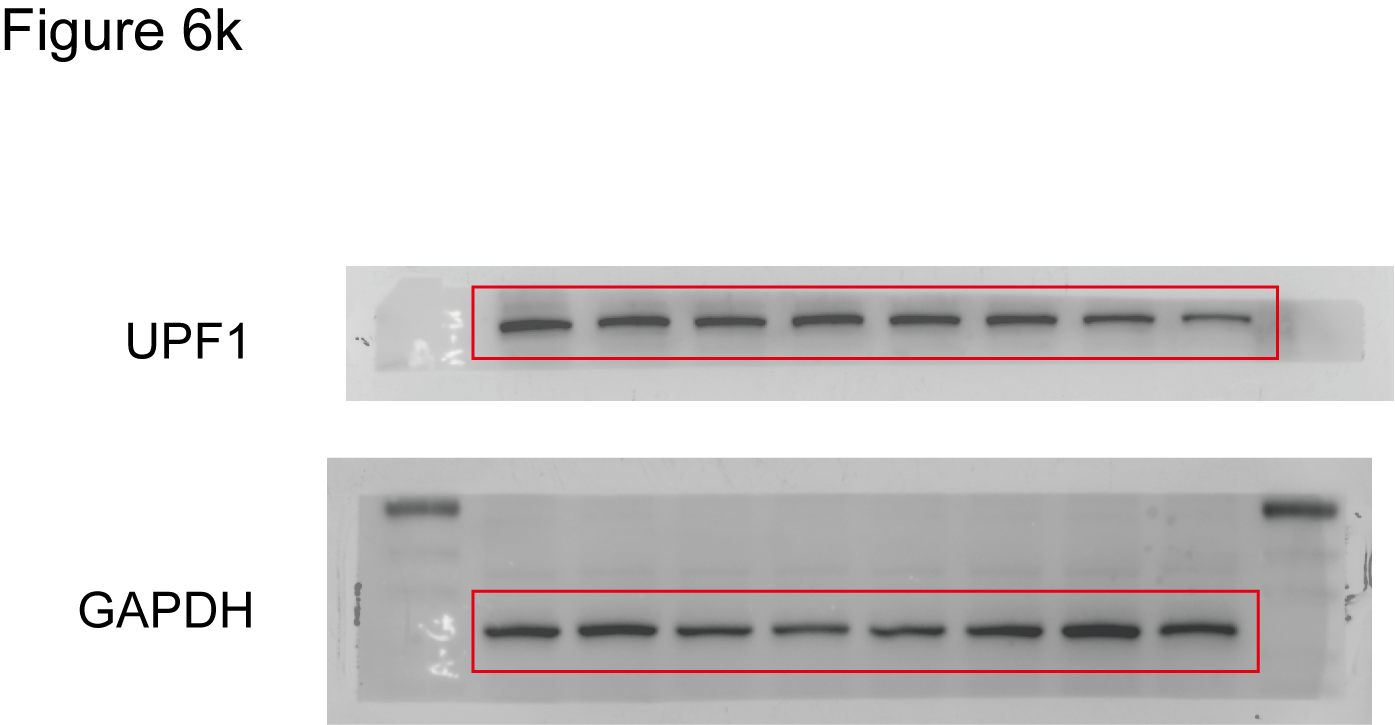

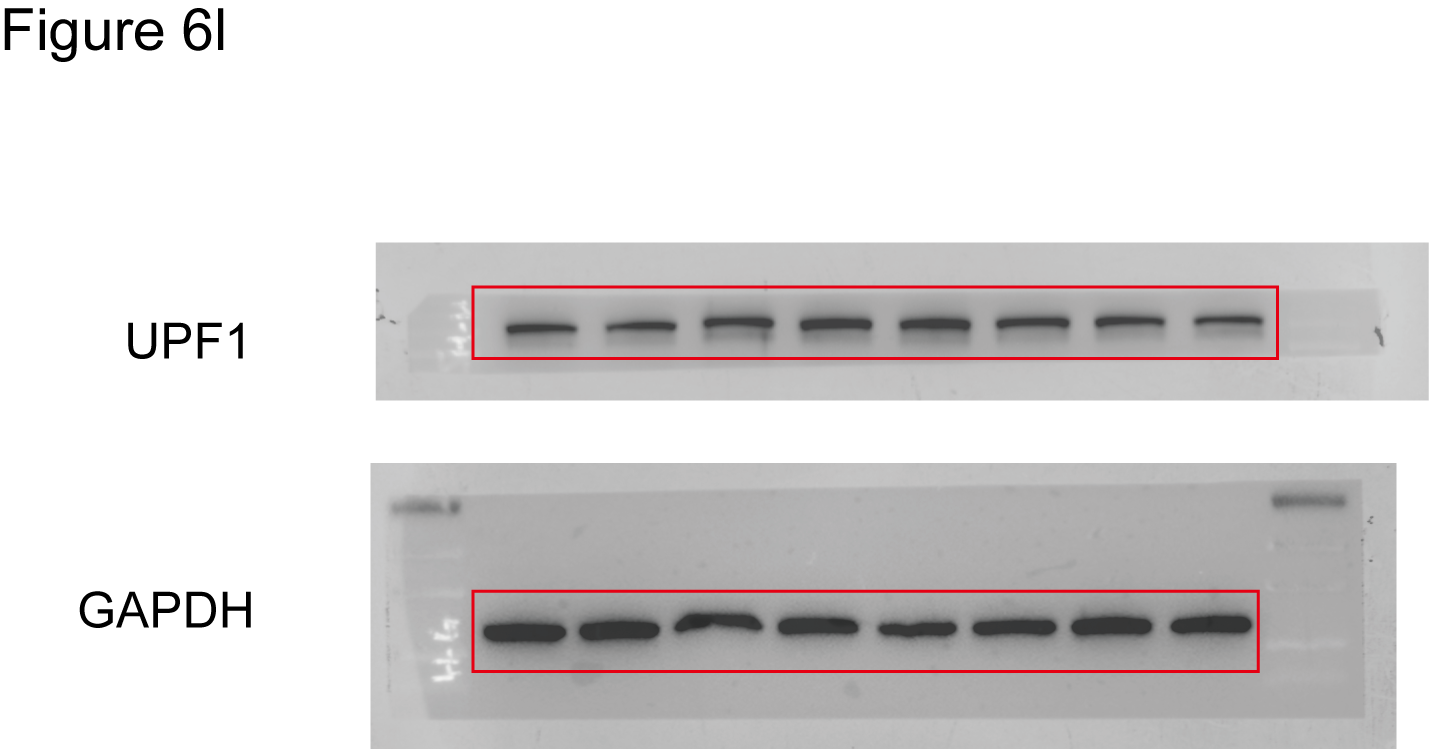

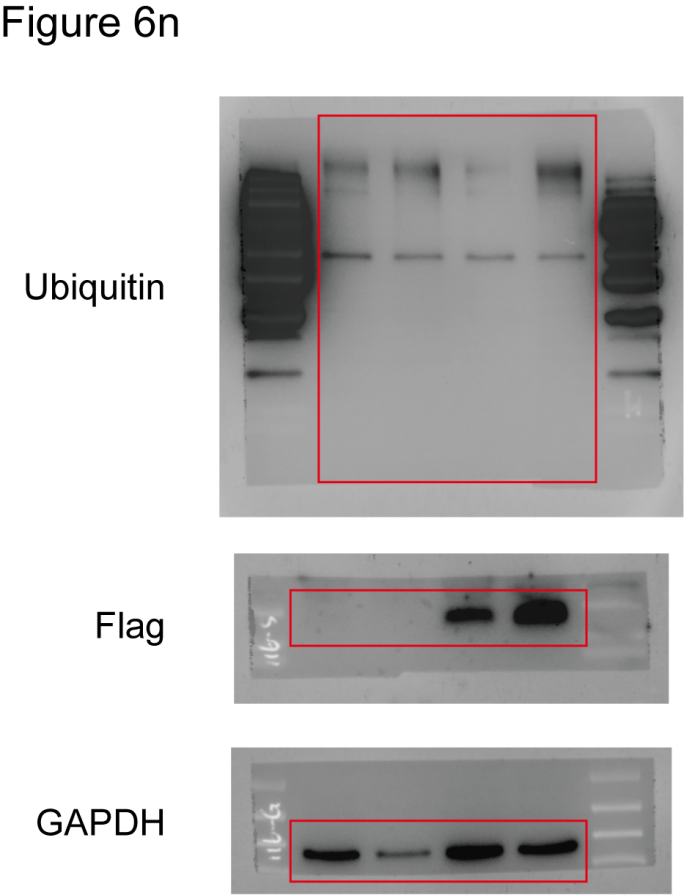

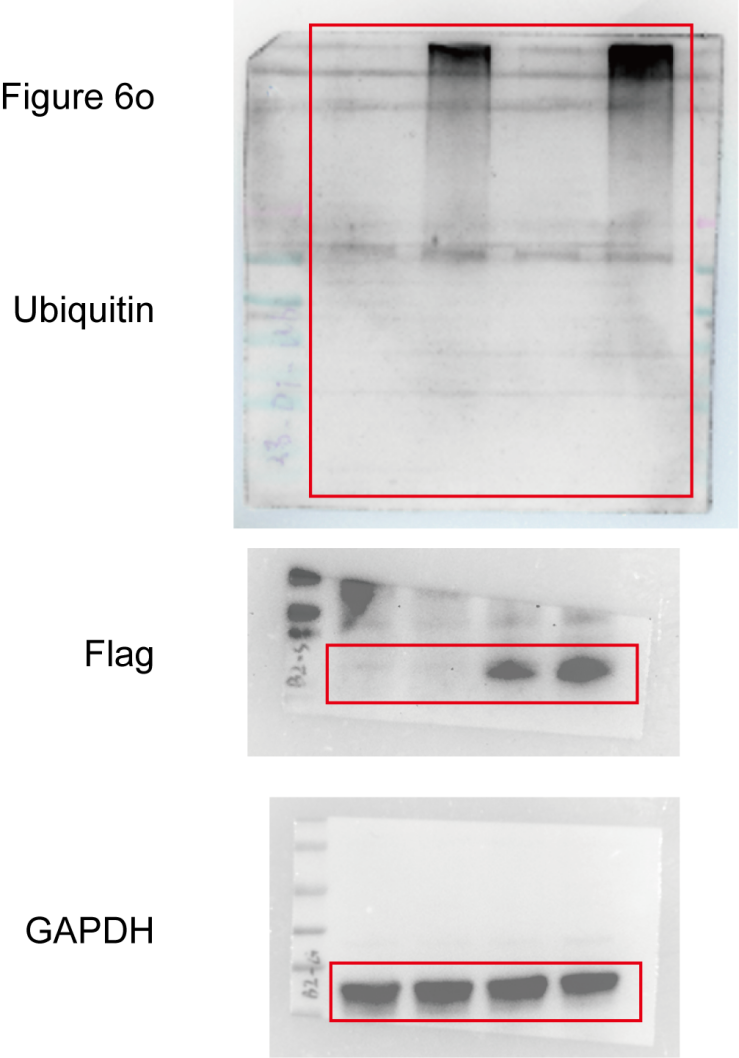

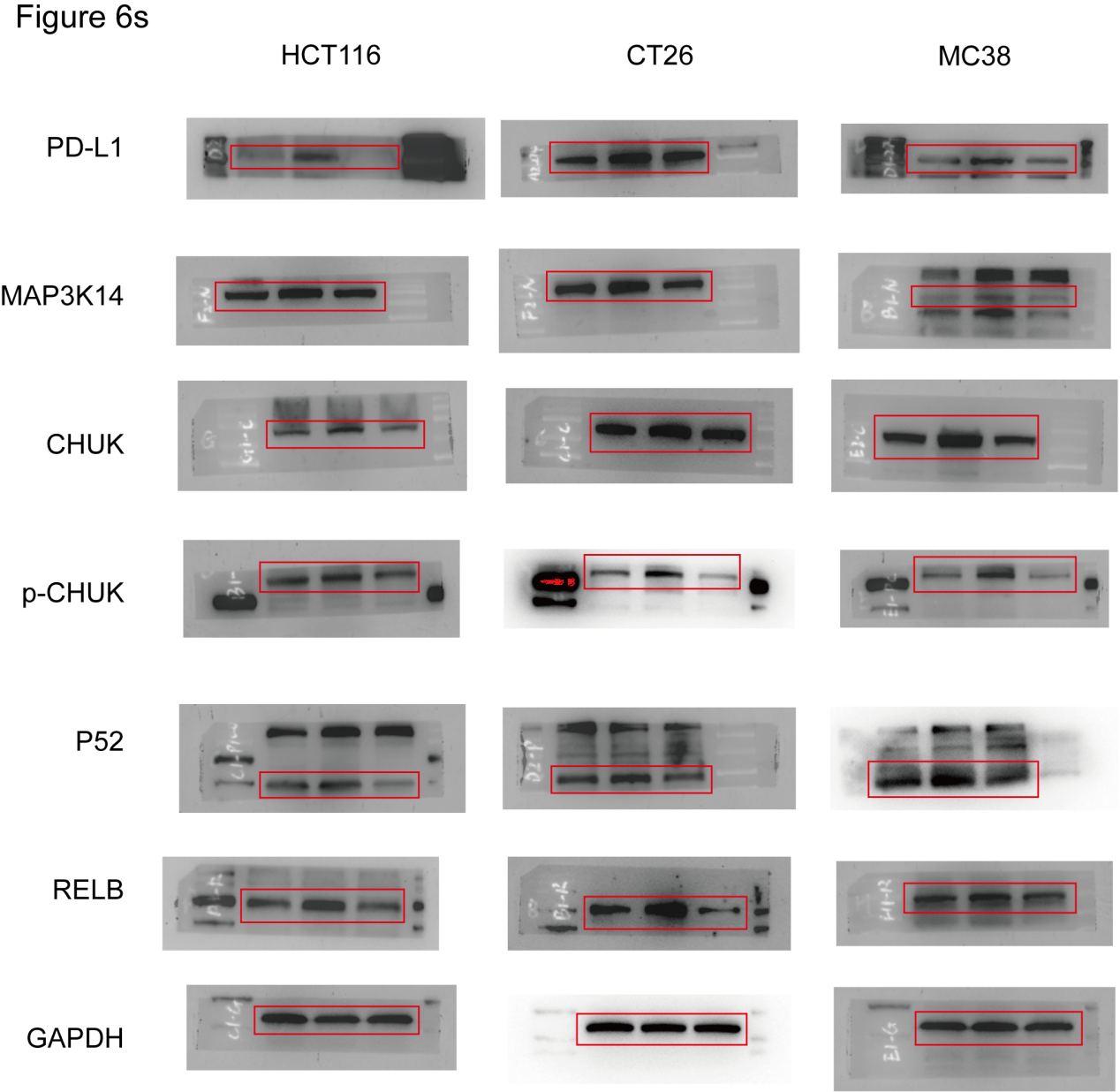

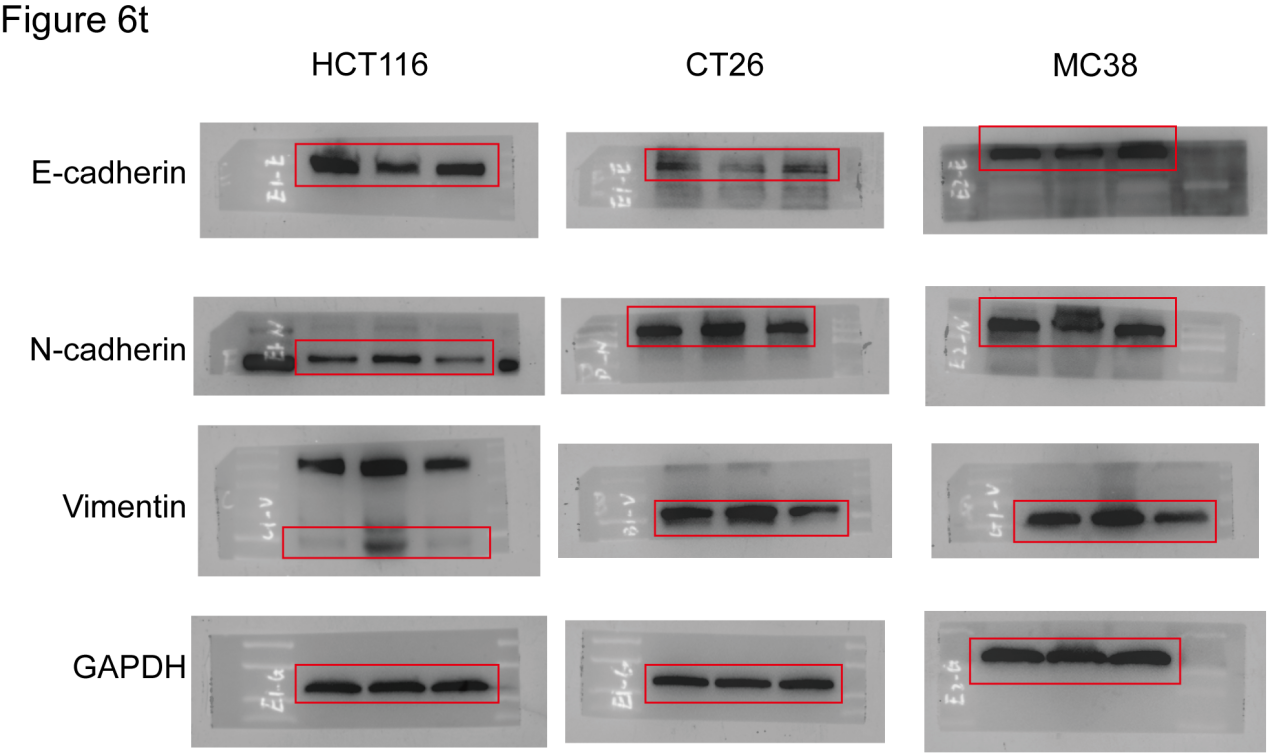
**

Supplement: Supplementary file 2 — Uncropped Western Blots [file 41419_2026_9032_MOESM2_ESM.docx]
